# Insecticidal Activity of Monoterpenoids Against *Sitophilus zeamais* Motschulsky and *Tribolium castaneum* Herbst: Preliminary Structure–Activity Relationship Study

**DOI:** 10.3390/ijms26073407

**Published:** 2025-04-05

**Authors:** Andrés G. Sierra-Quitian, Juliet A. Prieto-Rodríguez, Oscar J. Patiño-Ladino

**Affiliations:** 1 Departamento de Química, Facultad de Ciencias, Universidad Nacional de Colombia (UNAL), Sede Bogotá, Bogotá 111321, Colombia; asierraq@unal.edu.co; 2 Departamento de Química, Facultad de Ciencias, Pontifica Universidad Javeriana (PUJ), Bogotá 110231, Colombia; juliet.prieto@javeriana.edu.co

**Keywords:** *Minthostachys mollis*, *Tagetes zypaquirensis*, *Anethum graveolens*, *Satureja viminea*, fumigant toxicity, contact toxicity, monoterpenoids, structure–activity relationship

## Abstract

To contribute to the search for effective substances in pest control, this study describes the fumigant and contact toxicity against *Tribolium castaneum* and *Sitophilus zeamais* of four essential oils (EOs) and some of their major chemical constituents. The EOs from *Tagetes zypaquirensis*, *Anethum graveolens*, *Satureja viminea* and *Minthostachys mollis* were obtained by steam distillation and chemically characterized using GC–MS. In the development of research, some monoterpenoids were isolated from the EOs, others were purchased commercially, and some were synthesized from the most active monoterpenoids present in EOs. The main components in the EOs were dill ether (28.56%), α-phellandrene (25.78%) and carvone (23.67%) for *A. graveolens*, piperitone oxide (30.40%) and pulegone (25.91%) in *M. mollis*, pulegone (37.40%) and p-menth-3-en-8-ol (11.83%) for *S. viminea*, and dihydrotagetone (32.13%), myrcene epoxide (19.64%) and β-myrcene (5.30%) for *T. zypaquirensis*. The results highlight the fumigant action (LC_50_) and contact toxicity (LD_50_) of EO from *M. mollis* against *T. castaneum* (LC_50_ of 4.8 µL/L air and LD_50_ of 6.5 µg/insect) and *S. zeamais* (LC_50_ of 7.0 µL/L air and LD_50_ of 5.81 µg/insect). Among the chemical constituents evaluated, R-carvone **2,** piperitone oxide **5** and R-pulegone **6** stand out for their insecticidal potential against *S. zeamais* (LC_50_ between 3.0 and 42.4 µL/L, while LD_50_ between 14.9 and 24.6 µg/insect) and *T. castaneum* (LC_50_ between 2.2 and 4.8 µL/L, while LD_50_ between 4.8 and 13.1 µg/insect). Preliminary structure–activity analysis suggests that the presence of the carbonyl group with conjugated double bonds in cyclic monoterpenes is important for the insecticidal potential exhibited.

## 1. Introduction

Cereals play an important role in global food security, are an excellent source of nutrients, and can be stored for long periods of time [1,2,3]. However, during this storage period, cereals are usually affected by various types of pests that include insects, rodents and microorganisms [4,5]. *Sitophilus zeamais* Motschulsky (Coleoptera: Curculionidae), known as the “corn weevil”, is a cosmopolitan insect that attacks stored cereals, mainly maize, rice and wheat. This insect is considered a primary pest due to its ability to perforate grains, and its infestation in cereals tends to deteriorate the organoleptic properties of the grains, promoting the appearance of microorganisms and other pest insects. [6,7,8,9]. *Tribolium castaneum* Herbst (Coleoptera: Tenebrionidae), also known as the “red flour beetle”, is a secondary infestation pest that feeds mainly on perforated cereal grains and milling products. Infestation by this insect can also cause serious damage to the quality of grains and flour, highlighting the persistence of unpleasant odors and changes in color and flavor due to the benzoquinones secreted by these insects [10,11]. The control of this type of insect is usually carried out with insecticides of synthetic origin due to their effectiveness; however, some products are highly toxic to the environment and human health [12,13]

In the search for effective and safe insecticides to control stored grain pests, essential oils (EOs) are emerging as a promising alternative of natural origin due to their physicochemical properties, ecological roles and reported biological properties [14,15,16,17,18,19,20,21]. Many of these EOs are characterized as safe for humans, have low persistence under field conditions, have low toxicity to non-target organisms, and have been shown to leave minimal or no residues in food products [14,15]. In addition, it has been reported that because EOs consist of mixtures of volatile metabolites with different modes of action, the possibility of resistance development in insect pests is minimized [16,17]. A large body of research has shown that EOs from a variety of plant families could be used as a source of compounds to control *S. zeamais* and *T. castaneum* [18,19]. Research in this field has shown that EOs rich in monoterpenoids are characterized by their fumigant activity, while EOs rich in phenylpropanoids are characterized by their contact toxic activity [20,21].

Among the EOs with productive potential in this field are those from *Anethum graveolens L.* (Apiaceae), *Minthostachys mollis* (Benth.) Griseb. (Lamiaceae), *Satureja viminea* L. (Lamiaceae) and *Tagetes zypaquirensis* Humb. and Bonpl. (Asteraceae) due to their high extraction yields and their insecticidal properties [21,22,23,24,25,26,27,28,29,30,31,32,33,34,35,36,37,38]. *A. graveolens*, popularly known as dill, is a species native to Eastern Europe and the Mediterranean that is characterized by the production of an EO with high phellandrene and dill ether content [22,23,24]. Contact and fumigant toxicity and repellent activity against *S. zeamais* and fumigant, antifouling and repellent activity against *T. castaneum* have been reported for EOs from *A. graveolens* [25,26,27]. *M. mollis*, popularly known as muña, is a species native to South America that produces EOs with varied chemical composition in oxygenated monoterpenes depending on the collection site [28,29,30]. The insecticidal activity of these EOs against various organisms has been reported [31,32,33,34]. However, studies against pests of interest are preliminary; only contact toxicity has been reported against *S. zeamais* [35]. *S. viminea* is an aromatic species popularly known as mint or hibiscus mint, native to America and characterized by the presence of an EO rich in pulegone, p-menth-3-en-8-ol and β-caryophyllene [36,37]. Contact and fumigant toxicity and repellent activity against *S. zeamais* have been reported for EOs from *S. viminea* [21], but the chemical constituents responsible for the activity have not been determined, and the insecticidal activity against *T. castaneum* has not been reported. *T. zypaquirensis* is a species native to Ecuador and Colombia, popularly known as rudón, characterized by the production of an EO rich in acyclic monoterpenoids such as tagetone, dihydrotagetone and myrcene [34,38]. The insecticidal activity of *T. zypaquirensis* against *S. zeamais* and *T. castaneum* has not been reported, but there are reports in the literature of its use in the control of other insects [34]. The present study contributes to the characterization of the insecticidal potential of EOs from *A. graveolens*, *M. mollis*, *S. viminea* and *T. zypaquirensis* against *S. zeamais* and *T. castaneum*, as well as some of their main chemical constituents. In addition, some preliminary structure–activity relationships were established from the isolated, synthesized and commercially acquired monoterpenoids.

## 2. Results and Discussion

### 2.1. Chemical Composition and Insecticidal Action of EOs

GC–MS analysis of the four EOs and comparison with the data reported in the literature led to the tentative identification of 54 compounds, representing from 86 to 99% of the total composition (Table 1). The reported chemical composition allowed us to establish that the main compounds present in the EOs are monoterpenoids (61–99%), followed by sesquiterpenoids (0–33%).

The chemical composition of the EO from *A. graveolens* suggests that the major constituents are α-phellandrene (25.78%), Limonene (13.77%), dill ether (28.56%) and carvone (23.67%), which, although maintaining some typical components reported in the species, is significantly different from what has been described in other studies [42]. In this study, piperitone oxide (30.40%), pulegone (25.91%) and β-caryophyllene (8.17%) were determined as major components in the EO of *M. mollis*. Differences in some major components were observed with what was described in the literature; for example, the EO of *M. mollis* from Peru was mainly characterized by the presence of menthone (32.9%) and eucalyptol (28.0%) [43], while in the EO from Argentina, pulegone (76.3%) was reported as the main component [44]. In the EO of *S. viminea*, pulegone (37.40%), β-caryophyllene (11.33%) and p-menth-3-en-8-ol (11.83%) were determined as major constituents, which differs from what has been previously reported in other investigations for this species [45]. Finally, the main components determined in the EO of *T. zypaquirensis* are dihydrotagetone (32.13%) and 6,7-epoxymyrcene (19.64%), which agrees with previous studies reported for the genus and species, where the presence of this type of acyclic monoterpenes is common in their EOs [46].

Table 2 shows the results of contact and fumigant toxicity of the EOs of the four aromatic species against *S. zeamais* and *T. castaneum*. All EOs exhibited fumigant and contact toxicity against the two insects, with the fumigant activity (LC_50_) ranging from 23.1 to 4.8 µL/L air for *T. castaneum* and from 104.4 to 7.0 µL/L air for *S. zeamais*, while the contact toxicity (LD_50_) ranged from 86.1 to 6.5 µg/insect for *T. castaneum* and from 140.3 to 15.8 µg/insect for *S. zeamais*. The EOs of *M. mollis* and *S. viminea* were the most promising for the control of both insects. Their chemical compositions suggest the presence of some components common to both EOs that may be responsible for their insecticidal properties (Limonene, menthone, pulegone, piperitone, β-caryophyllene and humulene). This study highlights the first report on the insecticidal activity of *A. graveolens* (contact), *M. mollis* and *S. viminea* (contact and fumigant) EOs against *T. castaneum*, as well as the fumigant toxicity of *M. mollis* on *S. zeamais* and the insecticidal activity of *T. zypaquirensis* EO against the two insects studied.

The fumigant toxicity of *A. graveolens* EO against *S. zeamais* (LC_50_ 0.316 µL/cm^2^) and *T. castaneum* (LC_50_ 5.56 µL/L air) has been previously reported in the literature. However, these values differ from the results of this study (40.1 and 15.5 µL/L air, respectively), which is likely due to variations in the chemical composition of the EOs, as the previous report did not provide a chemical profile and the evaluation methods used were different [27,47]. In a previous study, we reported the insecticidal and repellent activity of *S. viminea* EO against *S. zeamais*, where p-menth-3-en-8-ol (45%) and pulegone (38%) were the main components [21]. In the present study, the insecticidal action against *S. zeamais* (LC_50_ 20.6 μL/L air) is confirmed, and the toxic effect on *T. castaneum* is reported for an essential oil of *S. viminea* containing p-menth-3-en-8-ol (11.8%), pulegone (37.4%) and β-caryophyllene (11.3%) as its major components. Comparing the results of both studies reveals that the insecticidal effect of *S. viminea* is related to the presence of pulegone; however, the greater insecticidal effect might be attributed to the lower proportion of p-menth-3-en-8-ol, suggesting that this compound may exert an antagonistic effect [21]. The toxic action by contact against *S. zeamais* for the EO of *M. mollis* has been reported in a previous study (LD_50_ of 4.15 μg/insect) [35]; however, these results differ from those obtained in this research due to changes in the bioassay used and the variation in the composition of some constituents of the EOs. The present study contributes to the chemical characterization and determination of the insecticidal properties of the EOs obtained from *A. graveolens*, *M. mollis*, *S. viminea* and *T. zypaquirensis*, highlighting their high content of monoterpenoids.

### 2.2. Determination of Insecticidal Action of Some Chemical Constituents Present in the EOs

**Figure 1:**
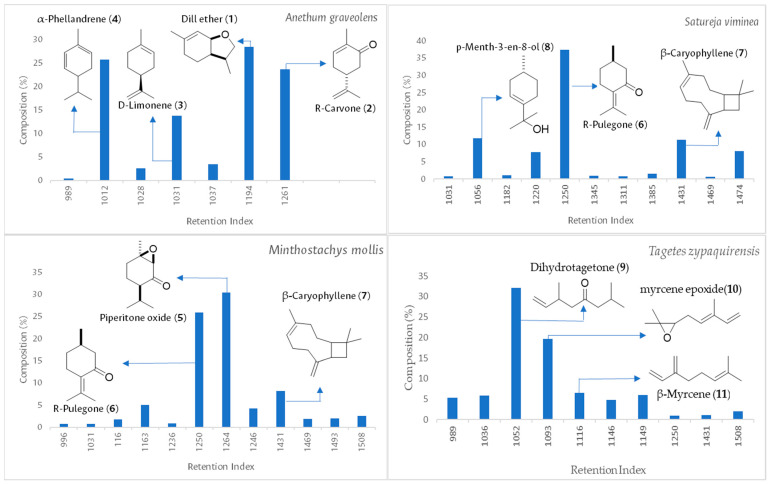
Major components present in the EOs of the four aromatic species (**1**–**11**): The compounds are arranged according to the linear retention index (retention index) versus their composition percentage (composition%).

To evaluate the fumigant and contact toxicity of certain chemical constituents present in the bioactive EOs, some compounds were isolated using chromatographic techniques, while others were purchased commercially. Dill ether (**1**) was isolated from the EO of *A. graveolens*, piperitone oxide (**5**) from *M. mollis*, and p-menth-3-en-8-ol (**8**) from the EO of *S. viminea*. In addition, dihydrotagetone (**9**) and myrcene epoxide (**10**) were obtained from the EO of *T. zypaquirensis*. The compounds R-carvone (**2**), D-limonene (**3**), α-phellandrene (**4**), R-pulegone (**6**), β-caryophyllene (**7**), and β-myrcene (**11**) were commercially purchased. Figure 1 illustrates the chemical structure of some of the major components present in the EOs used in the insecticidal tests, organized by linear retention index.

The results of the insecticidal activity of the main compounds present in the EOs, tested against *S. zeamais* and *T. castaneum*, are presented in Table 3 and are expressed as median lethal concentration (LC_50_) and median lethal dose (LD_50_). Overall, nine of the eleven compounds evaluated showed fumigant activity against both insects (**1**–**6** and **9**–**11**). Of these compounds, six showed contact activity against *S. zeamais* (**1**, **2**, **5**, **6**, **8** and **10**), while nine showed contact activity against *T. castaneum* (**1**–**6** and **8**–**10**). The LC_50_ and LD_50_ values ranged from 2 to 75 µL/L air and 9.7 to 88.1 µg/insect for *T. castaneum* and from 3 to 180 µL/L air and 14.9 to 75.6 µg/insect for *S. zeamais*. Among the compounds evaluated, R-carvone (**2**), piperitone oxide (**5**) and R-pulegone (**6**) stood out for their remarkable insecticidal potential. The results obtained for R-pulegone (**6**) are consistent with previous studies reporting an LD_50_ of 11.11 µg/insect for *T. castaneum* and an LC_50_ of 0.62 µL/L air for *S. zeamais* [48,49]. The data for compound **3** are also consistent with the literature regarding its fumigant toxicity against *S. zeamais* [48], while the results for **4** are consistent with previous studies on fumigant assays for *S. zeamais* and *T. castaneum* [21,48,49]. This study provides, for the first time, data on the insecticidal activity of **1**, **5**, **8**, **9** and **11** against *S. zeamais* and *T. castaneum*, which may be valuable information for the potential use of these compounds as insecticides.

It is important to highlight that those compounds (from **1** to **4**) exhibited moderate to high insecticidal activity against both insects, suggesting they contribute to the insecticidal effect of *A. graveolens* EO, with R-carvone **2** being the most active. However, **3** and **4** do not significantly contribute to the contact toxicity effect of *A. graveolens* EO against *S. zeamais*. On the other hand, piperitone oxide **5** and R-pulegone **6** showed insecticidal activity comparable to that of *M. mollis* EO, indicating that these compounds play a key role in the insecticidal effect of this oil. Regarding the major constituents of *S. viminea* EO, R-pulegone **6** exhibited high fumigant and contact activity against both insects, whereas compound **8** demonstrated only moderate contact toxicity, and compound **7** showed no insecticidal effect. These results confirm the critical role of R-pulegone **6** in the insecticidal activity of *S. viminea* EO. Among the major constituents evaluated from *T. zypaquirensis*, 6,7-epoxymyrcene **10** stands out as a significant contributor to the fumigant and contact activity of the EO against both insects.

The insecticidal activity results of the compounds also provide insights into preliminary structure–activity relationships. For example, the open-chain monoterpenoids (**9** and **11**) were the least active against both insect species in both fumigation and contact assays. However, the presence of an epoxide ring in an open-chain compound significantly enhances insecticidal activity. This is evident when comparing 6,7-epoxymyrcene (**10**) with myrcene (**11**), where the former was found to be 2 to 4 times more potent in both fumigation and contact toxicity assays. Among the cyclic monoterpenoids, those containing a carbonyl group within the six-membered ring (**2**, **5**, and **6**) exhibited the highest insecticidal activity against both insect species. These findings align with previous studies emphasizing the crucial role of the carbonyl group in insecticidal activity [49,50,51,52]. In addition, it was observed that an α,β-unsaturated carbonyl system with an exocyclic double bond significantly enhances insecticidal activity compared to a similar system with an endocyclic double bond, as evidenced by the differences in activity between compounds **6** and **2**. It is also observed that the position of the double bonds in monoterpenes **3** and **4** has no significant effect on the insecticidal activity against *S. zeamais*. However, against *T. castaneum*, the fumigant activity is enhanced when the double bonds are isolated (see compound **3**), while the contact toxicity is higher when the double bonds are conjugated within the six-membered ring (see **4**). Based on the previous results, compounds **2**, **5** and **6** were selected as candidates for the preparation of a series of derivatives with the aim of complementing the information from structure–activity relationship approaches against *S. zeamais* and *T. castaneum.*

### 2.3. Preliminary Structure–Activity Relationship Study of Monoterpenoids Against S. zeamais and T. castaneum

**Scheme 1:**
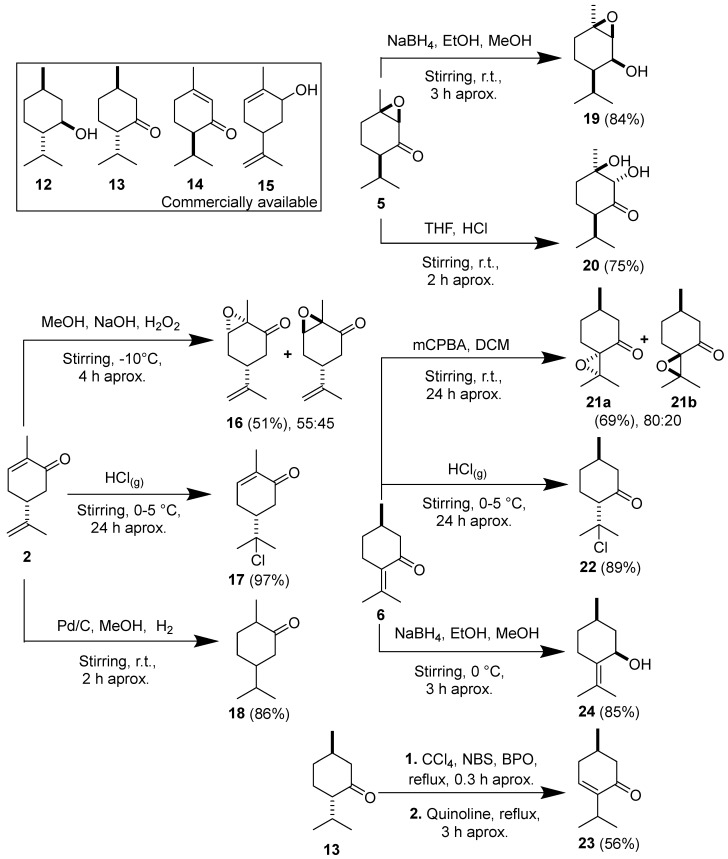
Synthesis of derivatives **16** to **24** from **2**, **5**, **6** and **13**.

To establish preliminary structure–activity relationships from the monoterpenoids with the greatest insecticidal potential (R-carvone **2**, piperitone oxide **5** and R-pulegone **6**), some commercial compounds with a similar structure were acquired, and the synthesis of some derivatives from **2**, **5** and **6** was carried out. In this way, some commercially available monocyclic monoterpenoids with oxygenated groups in different positions were acquired (L-menthol **12**, L-menthone **13**, piperitone **14** and carveol **15**). The synthesis of compounds **16** to **24** was carried out from **2**, **5** and **6**, applying typical reactions of epoxidation, hydrohalogenation, hydrogenation, isomerization, reduction and/or dihydroxylation (Scheme 1).

Diastereosiomers **16** were synthesized by reaction epoxidation in the presence of methanol and hydrogen peroxide in basic medium, starting from **2** [53], while epoxides **19** were obtained by typical reaction in the presence of metachloroperbenzoic acid (mCPBA), starting from **6** [54]. The diastereomeric ratio was determined by the integration in ^1^H-NMR for the signal from the methyl group in the alpha position to the carbonyl group. The absolute stereochemistry was determined by a NOESY experiment, considering that one of the chiral centers is known in the starting material, computational studies, optical rotations, and comparisons with literature data. Compounds **17** and **22** were obtained with good yields using typical hydrohalogenation conditions in an acid medium, starting from **2** and **6**, respectively [55]. The hydrogenation reaction was carried out in the presence of a hydrogen atmosphere and using palladium supported on carbon (Pd/C) as a catalyst, for the synthesis with good yields of **18** from **2** [56]. Compound **23** was obtained from **13** by a synthesis described in the literature that included two steps: an allylic bromination using N-bromosuccinimide and benzoyl peroxide, and then an elimination in the presence of quinoline [57]. Compounds **19** and **24** were obtained in good yields using typical conditions for the reduction of the carbonyl group in the presence of sodium borohydride (NaBH_4_), starting from **5** and **6**, respectively [58,59]. Compound **20** was obtained with a yield of 75% using acidic media (HCl 1.0 M) in tetrahydrofuran (THF) for the ring opening of epoxide, starting from **5** [60]. The synthesis of **17**, **19** and **20** with the methodologies used is reported for the first time. The NMR spectra obtained for the synthesized compounds are presented in the Supplementary Material (Figures S19–S36). The compounds **16** to **24** have been previously reported [53,54,55,57,59,61,62,63].

The insecticidal activity of the derivatives and commercial compounds was evaluated using contact and fumigation methods against *S. zeamais* and *T. castaneum*. The results, presented in Table 4, indicate that the LC_50_ and LD_50_ values ranged from 1.1 to 35.2 µL/L air and 1.0 to 85.5 µg/insect for *T. castaneum*, while for *S. zeamais* the values varied from 25.4 to 92.9 µL/L air and 14.2 to 263.0 µg/insect. Compound **12** also has reports of insecticidal activity against *T. castaneum* and *S. zeamais*, with LC_50_ values greater than 100 mg/L, which coincides with the results of this study, as no significant activity was observed [64,65]. Compound **13** has been previously reported to have insecticidal activity against *T. castaneum* using fumigation methodology, with a LC_50_ value of 26.03 µL/L of air, a result like that obtained in this study [66]. Regarding compound **14**, its insecticidal activity against *S. zeamais* and *T. castaneum* has been previously reported. However, in this study, it showed no activity against *S. zeamais*, making a direct comparison unfeasible. Additionally, its effectiveness against *T. castaneum* was significantly lower than previously reported values, with a LC_50_ of 500 µL/L of air [49,67]. For compound **15**, the results reported in the literature agree with those of this study against *T. castaneum* via fumigant action [68]. Finally, it is important to highlight that this study is the first to report the insecticidal activity of compounds **16** to **24** against *S. zeamais* and *T. castaneum* using both contact and fumigation methodologies.

The insecticidal activity of the derived and commercial compounds was evaluated through contact and fumigation toxicity bioassays against *S. zeamais* and *T. castaneum*. The LC_50_ and LD_50_ values (Table 4) ranged from 1.1 to 35.2 µL/L of air and 1.0 to 85.5 µg/insect for *T. castaneum* and from 25.4 to 92.9 µL/L of air and 14.2 to 263.0 µg/insect for *S. zeamais*. The comparison of the insecticidal activity of the evaluated compounds (**12**–**24**) with their precursors (**2**, **3**, **5**, and **6**) allowed the establishment of preliminary structure–activity relationships, considering the presence or absence of specific functional groups. Overall, the results demonstrate that cyclic monoterpene ketones exhibit high insecticidal potential against both target insects, in agreement with previous studies [51,69]. In particular, it was observed that menthone-type compounds (**5**, **6**, **13**, **14**, **21**–**23**) were approximately four times more active than carvomenthone-type compounds (**2**, **16**–**18**) against both insects using contact and insects using fumigation methods. The comparison between compounds **6** and **24**, as well as between **2** and **15**, revealed that the substitution of the carbonyl group with a hydroxyl group in monoterpene ketones significantly reduced toxicity by contact and inhalation in both insects, highlighting the structural importance of the carbonyl group in insecticidal activity.

Additionally, the comparison between compounds **2** and **18**, as well as between **6** and **13**, demonstrated that carbonyl conjugation with a double bond increased activity against *S. zeamais* by 3 to 42 times, whereas in *T. castaneum*, the effect was less pronounced. These results support previous literature reports on the significance of α,β-unsaturated ketones in insecticidal activity against *S. zeamais* and other insects, such as *Aedes aegypti* [51,70]. However, this study is the first to provide evidence of the positive influence of an α,β-unsaturated carbonyl group on insecticidal activity against *T. castaneum*. The results also indicate that the position of the double bond conjugated with the ketone influences insecticidal activity, both by contact in *T. castaneum* and by contact and inhalation in *S. zeamais*. The comparison between monoterpenoids with endocyclic and exocyclic α,β-unsaturated carbonyl groups suggests that an exocyclic double bond enhances fumigant activity against *S. zeamais*, as compound **6** was found to be 3 to 30 times more active than compounds **14** and **23**, respectively. A similar trend was observed in contact toxicity against both insects, as compound **6** was more active than compounds **14** and **23**.

**Figure 2:**
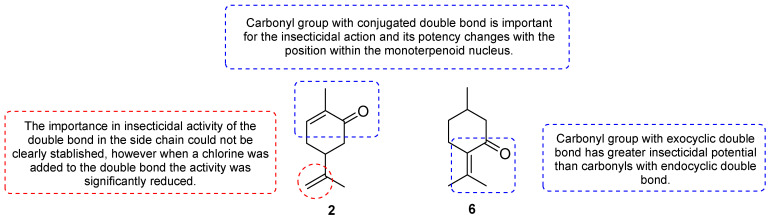
Preliminary structure–activity relationships against *T. castaneum* and *S. zeamais*.

Furthermore, the presence of vicinal epoxide groups with the carbonyl group generally tended to reduce insecticidal activity compared to molecules with α,β-unsaturated carbonyls (**5**, **6**, and **21**). In this regard, the insecticidal effect was less pronounced when the epoxide was part of a six-membered ring (**5**) than when it was located in the side chain of the cyclic monoterpenoid (**21**). However, in *T. castaneum*, compound **21** represented an exception to this observed trend, as it exhibited greater fumigant activity than its precursor, compound **6**. These results align with previously reported findings for an insecticidal study against *Leptinotarsa decemlineata* [71]. Finally, the presence of a halogen substituent, such as chlorine, in the side chain and without a double bond significantly reduced insecticidal activity against both insects, as evidenced by the comparison between compounds **6** and **22** and between **2** and **17**. This study contributes to the establishment of preliminary structure–activity relationships, illustrated in Figure 2, highlighting the importance of α,β-unsaturated carbonyl groups in cyclic monoterpenoids, particularly when the double bond is in an exocyclic position.

## 3. Materials and Methods

### 3.1. General Experimental Procedures

The GC–MS analysis was performed on a Shimadzu GC 2010 Plus gas chromatograph, which was coupled to a GCMS-TQ 8040 mass spectrometer (Shimadzu^©^, Kyoto, Japan) in electron impact (EI) mode, operating at 70 eV with a quadrupole analyzer in full scan mode at 4.57 s^−¹^. Mass spectra were acquired in the range of 40 to 400 *m*/*z*. Two different analyses were performed for the EOs using two orthogonal polarity columns: a DB-5MS column ((5%)-phenyl-methylpolysiloxane, 60 m × 0.25 mm × 0.25 μm) and a HP-INNOWax column (polyethylene glycol (PEG), 60 m × 0.25 mm × 0.25 μm) (Agilent Technologies, Santa Clara, CA, USA). Linear retention indices (LRIs) were calculated using a standard alkane solution (C_7_–C_40_) at 1000 ppm (Sigma-Aldrich^©^, Saint Louis, MO, USA).

The isolation and purification of the chemical compounds from the EOs and compounds obtained by synthetic modifications was carried out by Flash Chromatography (FC) on SiliaFlash^®^ P_60_ silica gel with a size of 25–40 μm (SiliCycle^®^ Inc., Quebec, QC, Canada). Chromatographic studies, fractionation monitoring and purifications were performed by thin-layer chromatography (TLC) on SiliaPlate^TM^ aluminum plates coated with silica gel P_60_ F_254_ of size 5–20 μm (SiliCycle^®^ Inc., Quebec, QC, Canada) using UV light (254 and 365 nm) and iodine vapors as developers. A Heidolph Hei-VAP rotary evaporator (Heidolph Instruments GmbH & Co. KG, Schwabach, Germany) was used in the solvent removal and recovery processes. The solvents used in chromatographic separations were commercially procured and of technical grade and were distilled and dried prior to use, while the other reagents used in this study were acquired commercially and were used without prior purification. Isolated compounds were characterized through H-NMR and APT experiments and compared with literature data. NMR measurements were conducted on a Bruker Advance AC-400 spectrometer (Bruker^®^, Hamburg, Germany) at 400 MHz for ^1^H and 100 MHz for ^13^C (APT) using deuterated chloroform (CDCl_3_) as the solvent at 25 °C. Chemical shifts (δ) are expressed in parts per million (ppm) and coupling constants (*J*) in Hertz (Hz). The optical rotations were measured using an Atago™ Polax-2L polarimeter (Atago Co., Ltd., Tokyo, Japan) at 25 °C with a sodium lamp at a wavelength of 589 nm.

### 3.2. Collection of Plant Material and Extraction EOs

#### 3.2.1. Plant Material

The aerial parts of the plant species were collected in some municipalities of the department of Cundinamarca (Colombia). *M. mollis* (Benth.) Griseb. (COL 631651), was collected in the rural area of San Antonio del Tequendama, while *S. viminea* L. (COL 631645) was collected on the Silvania-Fusagasugá road, and *T. zypaquirensis* Humb. and Bonpl. (COL 631650) was collected near the municipality of Tenjo. *A. graveolens* L. was commercially acquired from Suagá Organic Herbs S.A., located in the rural area of Úbate. The species were identified by biologist A. Casas, and reference specimens were deposited in the Herbario Nacional Colombiano belonging to the Instituto de Ciencias Naturales of the Universidad Nacional de Colombia. The collection of plant species was carried out under the contract of access to genetic resources and derived products No. 121 (01/22/2016), with OTROSI No. 21 celebrated between Ministerio de Medio Ambiente y Desarrollo Sostenible and Universidad Nacional de Colombia.

#### 3.2.2. Extraction of EOs

The fresh aerial parts of the four species collected were subjected to steam extraction for approximately 3 h. The EOs were recovered via condensation using a Clevenger-type apparatus, decanted and followed by drying with anhydrous sodium sulfate and storage in a refrigerator at 4 °C until use.

### 3.3. Chemical Characterization of Essential Oils

#### 3.3.1. Sample Preparation

For the sample preparation, a volume of 25 μL of each EO was taken and brought to a final volume of 1.0 mL with n-hexane. The standard hydrocarbon solution was prepared by dissolving 25 μL of a homologous hydrocarbon solution (C_8_–C_26_) to a final volume of 1.0 mL with n-hexane.

#### 3.3.2. Analysis by GC–MS

The chromatographic analysis started using the DB-5MS column with an injection volume in each analysis of 1 μL with a split injection (20:1) at an injection temperature of 280 °C. The carrier gas was helium (99.9995%) with a linear velocity of 25.5 cm/s and a constant flow rate of 1 mL/min. The temperature ramp started at 40 °C for 2 min, then increased to 123 °C (4 °C/min) and was held constant for 2 min. Subsequently, it was raised to 160 °C (4 °C/min) and maintained for an additional 5 min. It was further increased to 220 °C (5 °C/min) and held for 8 min, and finally rose to 280 °C (5 °C/min) and kept constant for 4 min, with a total run time of 75 min. In the second analysis, a HP-INNOWax column with an injection volume in each analysis of 1 μL with a split injection (20:1) at an injection temperature of 280 °C was analyzed. The carrier gas was helium (99.9995%) with a linear velocity of 25.5 cm/s and a constant flow rate of 1 mL/min. The temperature ramp started at 45 °C for 4 min, then increased to 120 °C (3 °C/min) and held for 2 min, and was finally raised to 250 °C (4 °C/min) and maintained for 8 min, with a total run time of 71.5 min.

#### 3.3.3. Tentative Identification of the Chemical Composition of EOs

The chemical constituents were determined tentatively by comparison of their mass spectral pattern and lineal retention indices (LRI) with those obtained from the NIST 14.L, Wiley 8.1 and Pherobase databases, as well as those published by Adams [39,40,72]. The LRI was calculated using paraffins and eluted under the same operational conditions described for EOs [39].

### 3.4. Obtaining of the Major Chemical Constituents Present in the EOs (**1**–**11**)

*Obtaining chemical constituents present in the EO from A. graveolens:* The EO of *A. graveolens* (1.0 g) was subjected to FC with hexane:DCM:EtOAc (90:6:4), which led to obtaining 5 fractions. Fractions 3 and 4 (384.2 mg) were combined and purified by FC with a mixture of hexane:DCM (70:30), which allowed for the obtaining of a colorless oil (**1**, 140.0 mg). Some chemical components present in the EO of *A. graveolens*, such as R-carvone (**2**, purity 85%), D-limonene (**3**, 95%) and α-phellandrene (**4**, 98%) were commercially acquired (Sigma-Aldrich^©^, Saint Louis, MO, USA).

*Dill ether (**1**):* Colorless oil, (140.0 mg), ${\left[\alpha \right]}_{D}^{25}$ = +27.7 (c 1.0, CHCl_3_), ^1^H-NMR (400 MHz, CDCl_3_): δ_H_ (ppm) 5.52 (dd, *J* = 3.0, 1.4 Hz, 1H, H-2), 4.25 (s, 1H, H-3), 4.07 (dd, *J* = 8.3, 7.3 Hz, 1H, H-9a), 3.31 (dd, *J* = 8.3, 7.0 Hz, 1H, H-9b), 2.08–1.98 (m, 1H, H-8), 1.96–1.84 (m, 2H, H-6), 1.76–1.64 (m, 2H, H-4 y H-5), 1.71 (s, 3H, H-7), 1.55–1.46 (m, 1H, H-5), 1.05 (d, *J* = 6.8 Hz, 3H, H-10). ^13^C-NMR (APT) (100 MHz, CDCl_3_): δ_C_ (ppm) 139.0 (C-1), 121.0 (C-2), 75.2 (C-3), 74.1 (C-9), 44.0 (C-4), 38.1 (C-8), 28.3 (C-6), 24.2 (C-5), 23.9 (C-7), 17.8 (C-10). The spectroscopic data matched those reported in the literature for dill ether [73], and the spectra can be consulted in Figures S9 and S10 of supplementary Materials.

*Obtaining chemical constituents present in the EO from M. mollis:* The EO of *M. mollis* (1.0 g) was subjected to FC with hexane:DCM (80:20), which led to obtaining five fractions. Fractions 3 and 4 (570.3 mg) were combined and purified by FC with hexane:DCM (75:25), which allowed for the obtaining a colorless oil (**5**, 400.7 mg). Some chemical components present in the EO of *M. mollis*, such as R-pulegone (**6**, 95%) and β-caryophyllene (**7**, 90%), were commercially acquired (Sigma-Aldrich^®^, Saint Louis, MO, USA).

*Piperitone oxide (**5**):* Colorless oil, (400.7 mg), ${\left[\alpha \right]}_{D}^{25}$ = −120.0 (c 1.0, CHCl_3_), ^1^H-NMR (400 MHz, CDCl_3_): δ_H_ (ppm) 3.06 (s, 1H, H-2), 2.39–2.30 (m, 1H, H-8), 2.17–2.11 (m, 1H, H-6), 1.88–1.69 (m, 4H, H-4, H-5), 1.42 (s, 3H, H-7), 0.90 (d, *J* = 7.0 Hz, 3H, H-9), 0.81 (d, *J* = 6.9 Hz, 3H, H-10). ^13^C-NMR (APT) (100 MHz, CDCl_3_): δ_C_ (ppm) 208.8 (C-1), 62.6 (C-2), 61.7 (C-3), 52.2 (C-6), 29.0 (C-8), 28.6 (C-4), 22.0 (C-7), 20.2 (C-9), 18.3 (C-10), 16.9 (C-5). The spectroscopic data matched those reported in the literature for piperitone oxide [74], and the spectra can be consulted in Figures S11 and S12 of Supplementary Materials.

*Obtaining chemical constituents present in the EO from S. viminea:* The EO of *S. viminea* (1.0 g) was subjected to FC with hexane:DCM (85:15), resulting in 6 fractions. Fractions 4 and 5 (642.8 mg) were combined and purified by FC with a mixture of hexane:DCM (70:30), which led to obtaining a colorless oil (**8**, 400.0 mg). β-Caryophyllene (**7**, 90%) was commercially acquired (Sigma-Aldrich^®^, Saint Louis, MO, USA).

*p-Menth-3-en-8-ol (**8**):* Colorless oil, (400.0 mg), ${\left[\alpha \right]}_{D}^{25}$ = +2.1 (c 1.0, CHCl_3_), ^1^H-NMR (400 MHz, CDCl_3_): δ_H_ (ppm) 5.73–5.65 (m, 1H, H-3), 2.12–2.04 (m, 2H, H-5), 1.77–1.69 (m, 2H, H-2), 1.62–1.51 (m, 2H, H-6), 1.30 (s, 3H, H-9), 1.29 (s, 3H, H-10), 1.22–1.16 (m, 1H, H-1), 0.93 (d, *J* = 6.3 Hz, 3H, H-7). ^13^C-NMR (APT) (100 MHz, CDCl_3_): δ_C_ (ppm) 143.6 (C-4), 118.6 (C-3), 73.0 (C-8), 33.9 (C-2), 31.5 (C-6), 29.0 (C-9 y C-10), 28.4 (C-1), 24.6 (C-5), 21.8 (C-7). The spectroscopic data matched those reported in the literature for p-menth-3-en-8-ol [75], and the spectra can be consulted in Figures S13 and S14 of Supplementary Materials.

*Obtaining chemical constituents present in EO from T. zypaquirensis:* The EO of *T. zypaquirensis* (1.0 g) was fractionated by FC with a mixture of hexane:DCM (80:20), resulting in 6 fractions. Fractions 1 and 2 (590.6 mg) were combined and subjected to FC purification with hexane:DCM (85:15), which led to obtaining a pale yellow oil (**9**, 500.0 mg). Fraction 4 (230.0 mg) was purified by FC with hexane:DCM (70:30), obtaining colorless oil (**10**, 150.0 mg). β-Myrcene (**11**, 90%) was commercially acquired (Aaron Chemicals LLC^®^, San Diego, CA, USA).

*Dihydrotagetone (**9**):* Pale yellow oil, (500.0 mg), ${\left[\alpha \right]}_{D}^{25}$ = +3.0 (c 1.0, CHCl_3_), ^1^H-NMR (400 MHz, CDCl_3_): δ_H_ (ppm) 5.80–5.68 (m, 1H, H-7), 5.03–4.88 (m, 2H, H-8), 2.78–2.66 (m, 1H, H-6), 2.47–2.27 (m, 2H, H-5), 2.25 (d, *J* = 6.9 Hz, 2H, H-3), 2.19–2.06 (m, 1H, H-2), 1.00 (d, *J* = 6.8 Hz, 3H, H-9), 0.90 (d, *J* = 1.1 Hz, 3H, H-1), 0.89 (d, *J* = 1.0 Hz, 3H, H-10). ^13^C-NMR (APT) (100 MHz, CDCl_3_): δ_C_ (ppm) 210.0 (C-4), 143.0 (C-7), 113.0 (C-8), 52.5 (C-3), 49.9 (C-5), 33.2 (C-6), 24.5 (C-2), 22.6 (C-1), 22.6 (C-10), 19.8 (C-9). The spectroscopic data matched those reported in the literature for dihydrotagetone [76], and the spectra can be consulted in Figures S15 and S16 of Supplementary Materials.

*Myrcene epoxide (**10**):* Colorless oil, (150.0 mg), ${\left[\alpha \right]}_{D}^{25}$ = 20.0 (c 1.0, CHCl_3_), ^1^H-NMR (400 MHz, CDCl_3_): δ_H_ (ppm) 6.37 (dd, *J* = 17.6, 10.8 Hz, 1H, H-2), 5.23 (d, *J* = 17.6 Hz, 1H, H-1), 5.07 (d, *J* = 11.0 Hz, 1H, H-1), 5.04 (s, 1H, H-9), 5.02 (s, 1H, H-9), 2.75 (t, *J* = 6.3 Hz, 1H, H-6), 2.47–2.38 (m, 1H, H-5), 2.35–2.26 (m, 1H, H-5), 1.76–1.68 (m, 2H, H-4), 1.30 (s, 3H, H-10), 1.25 (s, 3H, H-8). ^13^C-NMR (APT) (100 MHz, CDCl_3_): δ_C_ (ppm) 145.5 (C-3), 138.7 (C-2), 116.3 (C-9), 113.6 (C-1), 64.2 (C-6), 58.6 (C-7), 28.2 (C-4), 27.7 (C-5), 25.0 (C-10), 18.9 (C-8). The spectroscopic data matched those reported in the literature for myrcene epoxide [77], and the spectra can be consulted in Figures S17 and S18 of Supplementary Materials.

### 3.5. Obtention of Chemical Compounds **12** to **24**

Some compounds as L-menthol (**12**, 94%), L-menthone (**13**, 98%), piperitone (**14**, 90%) and carveol (**15**, 92%) were acquired commercially (Sigma-Aldrich^®^, Saint Louis, MO, USA) due to their structural similarity to monoterpenoids with greater insecticidal potential (**2**, **5** and **6**). Compounds **16** to **18** were synthesized from **2**, while **19** and **20** were synthesized starting with **5**, and compounds **21** to **24** were obtained from **6**.

*Carvone epoxide* (**16**): The epoxidation reaction of **2** was carried out by adapting the procedure described in the literature for the synthesis of **16** [53]. In a typical experiment, 300 mg of **2** (1.99 mmol) were dissolved in 10 mL of MeOH, and the resulting solution was cooled to −10 °C. Subsequently, 166 µL of NaOH (4.0 M, 1.33 mmol) and 560 µL of H_2_O_2_ (35%, 6.40 mmol) were added. The mixture was stirred to −10 °C and continuously monitored by TLC until the disappearance of **2** (approximately 4 h). The reaction was quenched by adding 0.5 mL of 4.0 M HCl, followed by 10.0 mL of a saturated solution of Na_2_S_2_O_3_. A liquid–liquid extraction was then performed with DCM (3 × 10.0 mL), and the organic phases were combined and dried over anhydrous Na_2_SO_4_, filtered, and the solvent was evaporated under reduced pressure. The resulting residue was purified by FC using a hexane:DCM (90:10) mixture, yielding **16** as a colorless oil corresponding to a mixture of diastereoisomers (168 mg, 51%, 55:45).

Compounds **16a** and **16b** (55:45): Colorless oil. ${\left[\alpha \right]}_{D}^{25}$ = +30.9 (c 1.0, CHCl_3_), ^1^H-NMR (CDCl_3_, 400 MHz): δ_H_ (ppm) 4.78 (t, *J* = 1.5 Hz, 1H, H-9), 4.72–4.69 (m, 1H, H-9), 3.43 (dd, *J* = 3.2, 1.2 Hz, 1H, H-5), 2.76–2.65 (m, 1H, H-3), 2.57 (ddd, *J* = 17.6, 4.7, 1.4 Hz, 1H, H-2), 2.42–2.31 (m, 1H, H-2), 2.01 (dd, *J* = 17.6, 11.6 Hz, 1H, H-4), 1.89 (ddd, *J* = 14.8, 11.1, 1.2 Hz, 1H, H-4), 1.70 (s, *J* = 1.1 Hz, 3H, H-10), 1.40 (s, 3H, H-7). ^13^C-NMR (APT) (CDCl_3_, 100 MHz): δ_C_ (ppm) 205.6 (C-1), 146.5 (C-8), 110.6 (C-9), 61.5 (C-5), 58.9 (C-6), 41.9 (C-2), 35.2 (C-3), 28.8 (C-4), 20.7 (C-10), 15.4 (C-7). The spectroscopic data matched those reported in the literature for carvone epoxide [53], and the spectra can be consulted in Figures S19 and S20 of Supplementary Materials.

*Carvone hydrochloride* (**17**): The hydrohalogenation reaction of compound **2** was performed following a previously described procedure for the hydrohalogenation of pulegone [78]. In a typical experiment, dry gaseous HCl was generated by adding 2.0 mL of 37% HCl to 4.0 mL of 98% H_2_SO_4_ in an ice bath. The produced gas was dried using CaCl_2_ and subsequently bubbled into a flask containing 400 mg of **2** (2.62 mmol), maintaining constant stirring and temperature between 0 and 5 °C. After complete addition of gas, the reaction mixture was stirred continuously at 0 °C until the disappearance of **2** was confirmed by TLC (approximately 24 h). The resulting crude product was purified by FC using a mixture of hexane:DCM (70:30), which led obtaining a yellow oil (**17**, 463 mg, 97%).

Compound **17**: Yellow oil. ${\left[\alpha \right]}_{D}^{25}$ = −27.5 (c 1.0, CHCl_3_), ^1^H-NMR (CDCl_3_, 400 MHz): δ_H_ (ppm) 6.77–6.73 (m, 1H, H-3), 2.70 (ddd, *J* = 16.0, 3.5, 1.8 Hz, 1H, H-4), 2.59–2.50 (m, 1H, H-4), 2.43–2.32 (m, 2H, H-6), 2.28–2.19 (m, 1H, H-5), 1.78 (bs, 3H, H-7), 1.59 (s, 3H, H-10), 1.57 (s, 3H, H-10). ^13^C-NMR (APT) (CDCl_3_, 100 MHz): δ_C_ (ppm) 199.4 (C-1), 144.5 (C-3), 135.4 (C-2), 72.2 (C-8), 47.4 (C-5), 40.2 (C-6), 30.7 (C-10), 30.4 (C-9), 28.0 (C-4), 15.7 (C-7). The spectroscopic data matched those reported in the literature carvone hydrochloride [78], and the spectra can be consulted in Figures S21 and S22 of Supplementary Materials.

*Carvomenthone* (**18**)*:* The synthesis of **18** from **2** was carried out under typical hydrogenation conditions in the presence of H_2_ and catalyzed by palladium on carbon (Pd/C) [79]. In a typical experiment, 200 mg of **2** (1.33 mmol) were dissolved in 13.0 mL of anhydrous methanol, and 13.0 mg of Pd/C (10%) were added. Subsequently, the reaction atmosphere was saturated with molecular hydrogen, and the mixture was stirred at room temperature until the disappearance of **2** was confirmed by TLC (approximately 2 h). The resulting mixture was filtered through quantitative Whatman paper, and the residue was washed with DCM (2 × 20 mL). The organic phase was dried over anhydrous Na_2_SO_4_ and concentrated under reduced pressure. The crude product was purified by FC using a hexane:DCM (90:10), which led obtaining a colorless oil (**18**, 143 mg, 86%).

Compound **18**: Colorless oil. ${\left[\alpha \right]}_{D}^{25}$ = 0 (c 1.0, CHCl_3_), ^1^H-NMR (CDCl_3_, 400 MHz): δ_H_ (ppm) 2.40–2.27 (m, 1H, H-6), 2.13–2.00 (m, 2H, H-2), 1.90–1.78 (m, 1H, H-3), 1.74–1.40 (m, 3H, H-5 y H-8), 1.38–1.26 (m, 2H, H-4), 1.00 (d, *J* = 6.5 Hz, 3H, H-7), 0.89 (d, *J* = 4.2 Hz, 3H, H-9), 0.87 (d, *J* = 4.4 Hz, 3H, H-10). ^13^C-NMR (APT) (CDCl_3_, 100 MHz): δ_C_ (ppm) 213.8 (C-1), 46.7 (C-6), 45.5 (C-3), 45.0 (C-2), 35.2 (C-5), 32.9 (C-8), 29.0 (C-4), 19.7 (C-9), 19.5 (C-10), 14.5 (C-7). The spectroscopic data matched those reported in the literature for carvomenthone [62], and the spectra can be consulted in Figures S23 and S24 of Supplementary Materials.

*6-Methyl-3-(1-methylethyl)-7-oxabicyclo[4.1.0]heptan-2-ol* (**19**): The synthesis of **19** from **5** was carried out by adapting typical carbonyl group reduction conditions using sodium borohydride (NaBH_4_) [59]. In a typical experiment, a solution of 98 mg of NaBH_4_ (2.60 mmol) in 4.7 mL of ethanol was added to a solution of **5** (300 mg, 2.39 mmol) in MeOH (3.5 mL) and water (0.9 mL) with constant stirring and 0 °C. The reaction mixture was stirred continuously, brought to room temperature and monitored by TLC until the disappearance of **5** (approximately 3 h). The resulting mixture was treated with a saturated NaCl solution (3 × 5.0 mL), and the aqueous layer was extracted with DCM (3 × 10.0 mL). The organic phases were combined, washed with water (3 × 5.0 mL), dried over anhydrous Na_2_SO_4_ and concentrated under reduced pressure. The crude product was purified by FC using a hexane:DCM (70:30), which led to obtaining a colorless oil (**19**, 463 mg, 97%).

Compound **19**: colorless oil. ${\left[\alpha \right]}_{D}^{25}$ = −45.8 (c 1.0, CHCl_3_), ^1^H-NMR (CDCl_3_, 400 MHz): δ_H_ (ppm) 4.19–4.11 (m, 1H, H-1), 3.23 (dd, *J* = 5.5, 1.0 Hz, 1H, H-6), 2.09–1.98 (m, 2H, H-8 y OH), 1.69–1.56 (m, 2H, H-4), 1.42–1.33 (m, 2H, H-2 y H-3), 1.34 (s, 3H, H-7), 1.19–1.06 (m, 1H, H-3), 0.98 (d, *J* = 6.6 Hz, H-9), 0.90 (d, *J* = 6.7 Hz, H-10). ^13^C-NMR (APT) (CDCl_3_, 100 MHz): δ_C_ (ppm) 64.7 (C-1), 62.3 (C-6), 61.5 (C-5), 47.0 (C-2), 31.2 (C-4), 27.9 (C-8), 23.0 (C-7), 21.0 (C-9 y C-10), 17.5 (C-3). The spectroscopic data were consistent with those reported in the literature for 6-methyl-3-(1-methylethyl)-7-oxabicyclo [4.1.0]heptan-2-ol [58], and the spectra can be consulted in Figures S2 and S26 of Supplementary Materials.

*2*,*3-dihydroxy-6-isopropyl-3-methylcyclohexan-1-one* (**20**): The synthesis of **20** from **5** was carried out by adapting the reaction conditions reported in the literature for the epoxide ring opening in acidic medium [80]. In a typical experiment, 1.5 mL of HCl (1.0 M, 1.5 mmol) was added dropwise to a solution of **5** (200 mg, 1.19 mmol) in THF (2.0 mL), under constant stirring and at room temperature. The reaction was monitored by TLC to verify the disappearance of **5** (approximately 2 h). Subsequently, 5.0 mL of brine was added and the mixture was extracted with DCM (3 × 10.0 mL), and the organic phases were combined and washed with water (2 × 10.0 mL). The organic layer was separated, dried over anhydrous Na_2_SO_4_ and concentrated under reduced pressure. The crude reaction product was purified by FC eluting with hexane:DCM (60:40), which led to obtaining **20** as a colorless oil (166 mg, 75%).

Compound **20**: colorless oil. ${\left[\alpha \right]}_{D}^{25}$ = −30.0 (c 1.0, CHCl_3_), ^1^H-NMR (CDCl_3_, 400 MHz): δ_H_ (ppm) 4.09 (s, 1H, H-6), 2.66–2.57 (m, 1H, H-2), 2.16–2.06 (m, 2H, H-4), 1.86–1.79 (m, 3H, H-3 y H-8), 1.38 (s, 3H, H-7), 0.95 (d, *J* = 4.7 Hz, 3H, H-9), 0.94 (d, *J* = 4.7 Hz, 3H, H-10). ^13^C-NMR (APT) (CDCl_3_, 100 MHz): δ_C_ (ppm) 205.2 (C-1), 76.9 (C-2), 68.8 (C-3), 51.5 (C-6), 32.7 (C-4), 26.6 (C-8), 25.6 (C-7), 22.8 (C-5), 21.1 (C-9), 19.4 (C-10). The spectroscopic data were consistent with those reported in the literature for 2,3-dihydroxy-6-isopropyl-3-methylcyclohexan-1-one [81], and the spectra can be consulted in Figures S27 and S28 of Supplementary Materials.

*Pulegone oxide* (**21**): The synthesis of **21** from **6** was carried out by adapting the typical conditions for the epoxidation of double bonds in the presence of m-chloroperoxybenzoic acid (mCPBA) [71]. In a typical experiment, **6** (300 mg, 1.97 mmol) was added to a solution of 50% mCPBA (883 mg, 2.56 mmol) in CHCl_3_ (15.5 mL). The resulting mixture was stirred at room temperature until the disappearance of **6**, which was monitored by TLC (approximately 24 h). Subsequently, 5.0 mL of a saturated NaHSO_3_ solution was added and the mixture was stirred for 30 min. The resulting mixture was extracted with CHCl_3_ (3 × 10.0 mL) and the organic phases were combined, dried over anhydrous Na_2_SO_4_, and the solvent was removed under vacuum. The obtained crude product was purified by FC using hexane:EtOAc (80:20), which led obtaining a colorless oil corresponding to a mixture of diastereoisomers (**21**, 230 mg, 69%, 8:2).

Compounds **21a**: colorless oil. ${\left[\alpha \right]}_{D}^{25}$ = −88.3 (c 1.0, CHCl_3_), ^1^H-NMR (CDCl_3_, 400 MHz): δ_H_ (ppm) 2.49–2.32 (m, 2H, H-6), 2.30–2.07 (m, 1H, H-5), 2.06–1.93 (m, 2H, H-4), 1.92–1.75 (m, 2H, H-3), 1.42 (s, 3H, H-9), 1.21 (d, *J* = 3.5 Hz, 3H, H-10), 1.06 (d, *J* = 6.0 Hz, 3H, H-7). ^13^C-NMR (APT) (CDCl_3_, 100 MHz): δ_C_ (ppm) 207.8 (C-1), 70.3 (C-2), 63.6 (C-8), 49.6 (C-6), 30.8 (C-5), 30.3 (C-4), 26.4 (C-3), 20.1 (C-7), 19.8 (C-9), 19.8 (C-10). The spectroscopic data was consistent with those reported in the literature for pulegone oxide [54], and the spectra can be consulted in Figures S29 and S30 of Supplementary Materials.

*Pulegone hydrochloride* (**22**)*:* The synthesis of **22** from **6** was carried out following a hydrohalogenation procedure like that used for the synthesis of **17** [55]. In a typical experiment, dry gaseous HCl was bubbled into a flask containing 400 mg of **6** (2.63 mmol), under continuous stirring and temperature between 0 and 5 °C. The resulting mixture was stirred continuously at 0 °C until the disappearance of **6**, which was monitored by TLC (approximately 24 h). The crude reaction product was purified by FC eluting with hexane:EtOAc (70:30), which led to the obtaining of **22** as a slightly yellow oil (439 mg, 89%).

Compound **22**: slightly yellow oil. ${\left[\alpha \right]}_{D}^{25}$ = −33.3 (c 1.0, CHCl_3_), ^1^H-NMR (CDCl_3_, 400 MHz): δ_H_ (ppm) 2.68 (ddd, *J* = 13.0, 4.6, 1.2 Hz, 1H, H-2), 2.55–2.47 (m, 1H, H-6), 2.27 (ddd, *J* = 12.3, 4.1, 2.2 Hz, 1H, H-6), 2.01 (td, *J* = 12.5, 1.2 Hz, 1H, H-5), 1.92–1.79 (m, 2H, H-3), 1.71 (s, 3H, H-10), 1.61 (s, 3H, H-9), 1.54 (td, *J* = 13.0, 3.2 Hz, 1H, H-4), 1.37 (tdd, *J* = 12.9, 11.3, 3.5 Hz, 1H, H-4), 0.98 (d, *J* = 6.3 Hz, 3H, H-7). ^13^C-NMR (APT) (CDCl_3_, 100 MHz): δ_C_ (ppm) 209.3 (C-1), 72.3 (C-8), 61.3 (C-2), 51.9 (C-6), 36.6 (C-5), 34.2 (C-4), 32.2 (C-9), 29.6 (C-3), 27.9 (C-10), 22.2 (C-7). The spectroscopic data were consistent with those reported in the literature for Pulegone hydrochloride [55], and the spectra can be consulted in Figures S31 and S32 of Supplementary Material.

*p-Menth-4-en-3-one* (**23**)*:* The synthesis of **23** from **13** was carried out by adapting the reaction conditions reported in the literature in presence of N-bromosuccinimide (NBS) and benzoyl peroxide (BPO) [57]. In a typical experiment, to a solution of **13** (300 mg, 1.95 mmol) in CCl_4_ (2.8 mL), NBS (50 mg, 0.28 mmol) and BPO (7 mg, 0.03 mmol) were added. The reaction mixture was heated at reflux temperature for 30 min, and after this time, it was cooled and filtered. Insoluble material was extracted with CCl_4_ (3 × 5.0 mL), and the organic phases were combined and washed with 10% Na_2_CO_3_ solution (3 × 10.0 mL), brine (3 × 10.0 mL) and water (3 × 10.0 mL). The organic phases were combined and dried over anhydrous Na_2_SO_4_, filtered and the solvent was removed by rotary evaporation. Distilled quinoline (660 mg, 5.11 mmol) was added to the resulting residue and heated at reflux until the disappearance of **13**, which was monitored by TLC (approximately 3 h). Finally, the crude product was purified by FC using hexane:DCM (95:5), which led to obtaining a colorless oil (**23**, 167 mg, 56%).

Compound **23**: Colorless oil. ^1^H-NMR (CDCl_3_, 400 MHz): δ_H_ (ppm) 6.62 (ddd, *J* = 5.7, 2.7, 1.1 Hz, 1H, H-3), 2.88–2.79 (m, 1H, H-8), 2.50–2.35 (m, 2H, H-6), 2.12–1.96 (m, 3H, H-5 and H-4), 1.01 (d, *J* = 6.2 Hz, 3H, H-7), 0.98 (d, *J* = 2.6 Hz, 3H, H-9), 0.97 (d, *J* = 2.6 Hz, 3H, H-10). ^13^C-NMR (APT) (CDCl_3_, 100 MHz): δ_C_ (ppm) 199.4 (C-1), 145.4 (C-2), 141.5 (C-3), 47.1 (C-6), 34.4 (C-4), 30.5 (C-5), 26.3 (C-8), 22.1 (C-9), 21.9 (C-10), 21.3 (C-7). The spectroscopic data were consistent with those reported in the literature for p-Menth-4-en-3-one [57], and the spectra can be consulted in Figures S33 and S34 of Supplementary Materials.

*Pulegol* (**24**): The synthesis of **24** from **6** was carried out using reduction conditions similar to those used in the preparation of **19**. In a typical experiment, a solution of NaBH_4_ (98 mg, 2.60 mmol) in ethanol (4.7 mL) was added to a solution of **6** (300 mg, 1.97 mmol) in MeOH (3.5 mL) and water (0.9 mL), with constant stirring and at a temperature of 0 °C. The reaction mixture was stirred continuously, brought to room temperature and monitored by TLC until the disappearance of **5** (approximately 3 h). The resulting mixture was treated with a saturated NaCl solution (3 × 5.0 mL), and the aqueous layer was extracted with DCM (3 × 10.0 mL). The organic phases were combined, washed with water (3 × 5.0 mL), dried over anhydrous Na_2_SO_4_ and concentrated under reduced pressure. The crude product was purified by FC using a hexane:DCM (70:30), which led obtaining a colorless oil (**24**, 143 mg, 85%).

Compound **24**: Colorless oil. ${\left[\alpha \right]}_{D}^{25}$ = −85.9 (c 1.0, CHCl_3_), ^1^H-NMR (CDCl_3_, 400 MHz): δ_H_ (ppm) 4.73 (t, *J* = 4.8 Hz, 1H, H-1), 2.35–2.19 (m, 2H, H-3), 1.80 (s, 3H, H-9), 1.70 (s, 3H, H-10), 1.66–1.53 (m, 3H, H-5 y H-6), 1.52–1.36 (m, 2H, H-4), 1.13 (d, *J* = 6.8 Hz, 3H, H-7). ^13^C-NMR (APT) (CDCl_3_, 100 MHz): δ_C_ (ppm) 132.7 (C-2), 126.7 (C-8), 68.3 (C-1), 39.5 (C-6), 31.9 (C-4), 26.7 (C-5), 22.2 (C-3), 21.6 (C-7), 20.6 (C-9), 19.9 (C-10). The spectroscopic data was consistent with those reported in the literature for pulegol [59], and the spectra can be consulted in Figures S35 and S36 of Supplementary Materials.

### 3.6. Assessment of Insecticidal Activity of EOs and Compounds

#### 3.6.1. Insect Rearing

Based on specimens characterized by the Instituto Colombiano Agropecuario (ICA) under report number R3823M0000425, colonies of *Sitophilus zeamais* Motschulsky and *Tribolium castaneum* Herbst were established. The insect breeding stock was maintained in a growth chamber under controlled conditions of darkness, humidity (65 ± 5% RH), and temperature (27 ± 1 °C). *S. zeamais* insects were kept in previously washed and dried porva corn, while *T. castaneum* was kept in thermally treated wheat flour. Adult insects aged between 6 and 10 days after emergence were used for the various activity tests [21,48].

#### 3.6.2. Fumigant Toxicity Assay

The volatile toxicity of *A. graveolens*, *M. mollis*, *S. viminea*, and *T. zypaquirensis* EOs, as well as the isolated and synthesized compounds, was evaluated against *S. zeamais* and *T. castaneum* using the vial-in-vial method [21,82]. Ten unsexed insects of *S. zeamais* or *T. castaneum* were placed in a 22 mL vial containing a 2 cm diameter filter paper disc at the top. Doses ranging from 2.2 to 0.025 μL were applied, resulting in final concentrations of 500–5 ppm for EOs and 200–1 ppm for chemical compounds, with *n*-hexane used as a diluent when necessary. To prevent direct contact with the EOs or compounds, a 15% PTFE solution was applied to the top of the vial for *S. zeamais*. Nuvan 50^®^ E.C. (dichlorvos—100 μL/L of air) was used as a positive control. Each assay was conducted with five replicates and two independent repetitions under controlled conditions (28 ± 1 °C and 70 ± 5% RH). Insect mortality was recorded after 24 h, and the percentage of mortality was calculated using Abbott’s formula. Mortalities obtained at different concentrations were analyzed through probit linear regression using SPSS software (SPSS Statistics 27, IBM^©^, Armonk, NY, USA) to estimate their lethal concentrations (LC_50_).

#### 3.6.3. Topical Contact Toxicity Assay

The contact toxicity of *A. graveolens*, *M. mollis*, *S. viminea*, and *T. zypaquirensis* EOs, as well as the isolated and synthesized compounds, were evaluated against *S. zeamais* and *T. castaneum* using the topic method [21,82]. Ten unsexed insects of *S. zeamais* or *T. castaneum* were immobilized by exposure to low temperatures (0 °C) in a Petri dish. Different amounts of EOs or compounds (0.025 to 0.20 μL) were applied directly to the insect’s prothorax using a microsyringe, with n-hexane as a diluent when necessary. The negative control consisted of the solvent used in the solutions, while the positive control was Hawker 25EC^®^ (Cypermethrin—0.2 μL/insect). After treatment, each insect was transferred to a 22 mL glass vial and placed in a culture chamber under controlled temperature and humidity conditions (darkness, 28 ± 1 °C, 70 ± 5% RH). Each assay was performed with five replicates and two independent repetitions under standardized conditions. Insect mortality was recorded 24 h post-treatment, and the percentage of mortality was calculated. Mortalities obtained at different concentrations were analyzed using probit linear regression with SPSS software (SPSS Statistics 27, IBM^©^, Armonk, NY, USA) to estimate their lethal doses (LD_50_).

#### 3.6.4. Data Analysis

The statistical treatment performed corresponds to an inferential analysis by means of a lineal regression probit, considering the test assumptions (normality, homogeneity of variances, independence, randomness and outliers) to determine if there were significant differences in the trials. These analyses were performed in the SPSS software (SPSS Statistics 27, IBM^©^, Armonk, NY, USA). All the results reported correspond to the mean of five replicates and their respective standard deviation, using a statistical significance of *p* < 0.05.

## 4. Conclusions

This study contributes to the chemical characterization and insecticidal activity of the essential oils (EOs) from *A. graveolens*, *M. mollis*, *S. viminea* and *T. zypaquirensis* against *S. zeamais* and *T. castaneum*. Notably, it provides the first report on the insecticidal activity of *A. graveolens* (contact toxicity), *M. mollis* and *S. viminea* (contact and fumigant toxicity) against *T. castaneum*, the fumigant toxicity of *M. mollis* against *S. zeamais*, and the insecticidal action of *T. zypaquirensis* EO on both insects. Furthermore, this study enhances the understanding of contact and fumigant toxicity of the major chemical constituents of these EOs, highlighting the insecticidal potential of the monoterpenoids R-carvone (**2**), piperitone oxide (**5**), and R-pulegone (**6**), as well as reporting for the first time the insecticidal activity (both contact and fumigant) of compounds **1**, **5**, **8**, **9**, and **10** against *S. zeamais* and *T. castaneum*. Additionally, this research contributes to the study of structure–activity relationships in stored-product insect pests, demonstrating the importance of the α,β-unsaturated carbonyl groups in cyclic monoterpenoids on the insecticidal action, particularly when the double bond is in an exocyclic position. These findings provide valuable insights for the development of plant-based insecticidal alternatives for the management of stored-product pests.

## Figures and Tables

**Table 1:** Chemical composition of EOs from *A. graveolens*, *M. mollis*, *S. viminea* and *T. zypaquirensis*.

Compound	DB-5MS		HP-INNOWax		% Relative			
	Exp LRI	Ref LRI	Exp LRI	Ref LRI	*A.G*	*M.M*	*S.V*	*T.Z*
α-Pinene	937	932–939	1028	1019–1030		0.25	0.18	
Isopropyl tiglate	966	959–976	-	-		0.20	0.21	0.27
Sabinene	979	960–980	-	-		0.13	0.16	0.35
β-Pinene	983	980–990	-	-		0.29	0.3	
**β-Myrcene**	989	986–994	1147	1145–1187	0.40			**5.3**
3-Octanol	996	991–995	1383	1368–1400		0.77		
**α-Phellandrene**	1012	1005–1032	-	-	**25.78**			
o-Cymene	1028	1026–1036	-	-	2.51			
**Limonene**	1031	1031–1039	-	-	**13.77**	0.75	0.8	0.2
(E)-β-Ocimene	1036	1037–1043	1270	1242–1270				5.83
β-Phellandrene	1037	1031–1053	1238	1189–1241	3.46			
Eucalyptol	1038	1031–1039	-	-				0.49
Dihydrotagetone	1052	1047–1082	1285	1268–1319		0.13		**35.39**
**p-Menth-3-en-8-ol**	1056	1147–1149	1613	1600–1621			**11.83**	
p-Mentha-3,8-diene	1075	1070–1076	-	-			0.3	
Ipsenone	1085	1083–1086	1440	1444				1.26
**6,7-Epoxymyrcene**	1093	1092–1096	1399	1398–1415				**19.64**
Linalool	1100	1096–1101	1580	1557–1581		0.12	0.33	0.29
4-t-Pentylcyclohexene	1116	1100–1128	1724	1720				6.49
3-Octanyl acetate	1116	1102–1123	1438	1424–1490		1.79		
(Z)-Epoxyocimene	1128	1120–1132	-	-				0.4
(E)-Tagetone	1146	1144–1149	1500	1501–1522				4.74
(Z)-Tagetone	1149	1147–1152	1501	1500–1517				6.00
Menthone	1163	1148–1164	-	-		5.10	0.23	
(Z)-Isocitral	1169	1164–1184	-	-				0.18
Cis-Linalool oxide	1172	1170–1174	-	-				0.37
Isopulegone	1182	1157–1179	1590	1582–1597			1.13	
**Dill ether**	1194	1183–1194	1489	1484–1529	**28.56**			
γ-Terpineol	1201	1195–1199	1685	1684–1695				0.44
trans-Pulegol	1220	1214–1221	-	-			7.79	
Perilla ketone	1236	1230–1248	-	-		0.88		
β-Citral	1241	1240–1242	-	-		0.41		
Piperitone	1246	1243–1247	-	-		4.63		
**Pulegone**	1250	1209–1237	1665	1661–1665		**25.91**	**37.4**	0.97
**Carvone**	1261	1242–1272	-	-	**23.67**			
**Piperitone oxide**	1264	1230–1251	1712	1700–1722		**31.70**		
Isopulegyl acetate	1267	1277–1309	1695	1581–1608		2.00		
(E)-Citral	1269	1267–1270	2039	1721–1737		0.62		
Carvacrol	1291	1286–1299	2217	2215–2219		0.43		
Dihydrocarveol acetate	1311	1307–1344 *	-	-			0.81	
Myrtenyl acetate	1327	1326–1332	-	-			0.21	
Piperitenone	1345	1340–1349	1710	1705–1739		2.03	0.95	
α-Copaene	1385	1372–1389	1504	1488–1520		0.19	1.51	
β-Bourbonene	1396	1387–1401	-	-		0.50		
**β-Caryophyllene**	1431	1418–1449	1627	1594–1657		**8.17**	**11.33**	1.09
α-Humulene	1469	1446–1464	1691	1660–1710		1.87	0.73	0.47
Alloaromadendrene	1474	1458–1478	1634	1616–1662			**8.14**	0.18
Germacrene D	1493	1485–1519	1722	1716–1724		2.07	2.87	0.40
Bicyclogermacrene	1508	1494–1517	1747	1736–1738		2.57	2.34	1.97
δ-Cadinene	1531	1523–1531	1769	1764–1772			2.76	0.25
14-Hydroxycaryophyllene	1662	1660–1667	-	-		0.29		
(E)-14-Hydroxy-9-epicaryophyllene	1668	1660–1670	1969	1966–1989			1.21	
Not identified	1724	-	-	-				1.55
Monoterpenoids					98.15	76.14	61.61	82.43
Sesquiterpenoids					0.00	17.66	32.72	4.36
Total					98.15	93.8	94.33	86.79

A.G = *A. graveolens*; M.M = *M. mollis*; S.V = *S. viminea*, T.Z = *T. zypaquirensis*; Exp LRI = experimental lineal retention index; Ref LRI = Reference lineal retention index; % Relative: Relative percentage of EO composition; (Ref: the NIST WebBook; Pherobase Kovats Index) [39,40].* Retention time of Diablo Analytical [41].

**Table 2:** Results of fumigant action (LC_50_) and contact toxicity (LD_50_) of the EOs against *S. zeamais* and *T. castaneum*.

EOs	*T. castaneum*		*S. zeamais*	
	Fumigant LC_50_ (µL/ L Air)	Contact LD_50_ (µg/Insect)	Fumigant LC_50_ (µL/L Air)	Contact LD_50_ (µg/Insect)
*T. zypaquirensis*	23.1 (12.7–33.3)	66.6 (49.8–87.7)	104.4 (74.4–143.2)	91.1 (71.7–111.4)
*S. viminea*	6.4 (3.9–9.7)	16.4 (10.9–24.9)	20.6 (12.2–31.9)	24.6 (12.8–38.3)
*M. mollis*	4.8 (3.3–6.8)	6.5 (4.6–9.3)	7.0 (6.1–8.0)	15.8 (9.3–24.2)
*A. graveolens*	15.5 (13.1–18.3)	86.1 (75.1–99.3)	40.1 (35.4–46.1)	140.3 (124.5–160.6)
Dichlorvos	2.1 (1.5–3.8)	N/A	1.0 (0.1–1.9)	N/A
Cypermethrin	N/A	1.0 (0.1–2.0)	N/A	10.5 (0.1–20.0)

The concentrations evaluated ranged from 2.0 to 200.0 µL/L of air for the fumigation assays and from 1.0 to 200.0 µg/insect for the contact assays. The results are expressed as the mean of five replicates (n = 5) and two repetitions, along with their respective confidence intervals. The annexes present the slope and intercept values, with a confidence interval and a *p*-value < 0.05. N/A: Not applicable; LC_50_: Median lethal concentration; LD_50_: Median lethal dose.

**Table 3:** Fumigant and contact toxicity of major components present in EOs against *T. castaneum* and *S. zeamais*.

Compound	*S. zeamais* LC_50_ and LD_50_ (Confidence Intervals 95%)		*T. castaneum* LC_50_ and LD_50_ (Confidence Intervals 95%)	
	µL/L Air	µg/Insect	µL/L air	µg/Insect
**1**	88.8 (81.9–95.0)	66.7 (54.0–87.7)	13.5 (12.1–13.0)	9.7 (8.2–18.0)
**2**	42.4 (28.7–63.3)	16.3 (14.4–18.2)	4.3 (1.3–7.6)	4.8 (2.9–6.7)
**3**	91.8 (87.0–99.7)	Not active	11.3 (9.0–14.1)	73.1 (58.8–87.4)
**4**	92.0 (84.9–107.8)	Not active	24.0 (14.2–31.1)	27.0 (12.7–44.8)
**5**	14.5 (12.9–16.1)	24.6 (14.4–37.4)	4.8 (3.4–6.7)	5.9 (5.1–6.8)
**6**	3.0 (3.3–2.7)	14.9 (12.1–18.7)	2.2 (2.0–2.4)	13.1 (10.3–15.9)
**7**	Not active			
**8**	Not active	75.6 (52.1–120.9)	Not active	78.6 (52.9–123.2)
**9**	180.4 (161.1–208.2)	Not active	42.1 (38.3–46.6)	88.1 (65.1–120.3)
**10**	37.1 (32.5–42.3)	50.8 (43.6–59.0)	16.9 (9.9–23.4)	49.0 (37.2–61.7)
**11**	104.0 (88.6–122.9)	Not active	75.1 (57.9–111.0)	105.2 (86.3–131.8)
Dichlorvos	2.1 (1.5–3.8)	N/A	1.04 (0.1–1.9)	N/A
Cypermethrin	N/A	1.0 (0.1–2.0)	N/A	10.5 (0.1–20.0)

The concentrations evaluated ranged from 2.0 to 200.0 µL/L of air for the fumigation assays and from 1.0 to 200.0 µg/insect for the contact assays. The results are expressed as the mean of five replicates (n = 5) and two repetitions, along with their respective confidence intervals. The annexes present the slope and intercept values, with a confidence interval and a *p*-value < 0.05. N/A: Not applicable; LC_50_: Median lethal concentration; LD_50_: Median lethal dose.

**Table 4:** Fumigant and contact toxicity of derivates of major components present in EOs against *T. castaneum* and *S. zeamais*.

Compound	*S. zeamais* LC_50_ and LD_50_ (Confidence Intervals 95%)		*T. castaneum* LC_50_ and LD_50_ (Confidence Intervals 95%)	
	µL/L Air	µg/Insect	µL/L Air	µg/Insect
**2**	42.4 (28.7–63.3)	16.3 (14.4–18.2)	4.3 (1.3–7.6)	4.8 (2.9–6.7)
**3**	91.8 (87.0–99.7)	Not active	11.3 (9.0–14.1)	73.1 (58.8–87.4)
**5**	14.5 (12.9–16.1)	24.6 (14.4–37.4)	4.8 (3.4–6.7)	5.9 (5.1–6.8)
**6**	3.0 (3.3–2.7)	14.9 (12.1–18.7)	2.2 (2.0–2.4)	13.1 (10.3–15.9)
**12**	Not active			
**13**	25.4 (22.0–29.4)	38.7 (26.6–57.6)	2.7 (1.9–3.5)	16.3 (12.0–23.2)
**14**	Not active	14.2 (8.7–19.0)	2.8 (1.6–3.9)	5.5 (4.7–7.1)
**15**	Not active	44.0 (35.4–55.5)	No active	21.1 (13.4–29.7)
**16**	Not active	38.8 (32.1–45.4)	25.4 (20.8–31.0)	19.8 (17.0–21.7)
**17**	Not active	69.1 (47.3–105.5)	Not active	85.5 (70.9–107.4)
**18**	Not active	28.3 (26.4–31.3)	Not active	1.0 (0.7–3.9)
**19**	Not active	109.7 (67.3–158.7)	Not active	67.3 (11.5–38.5)
**20**	41.8 (34.6–51.2)	24.5 (17.6–31.4)	4.5 (3.5–5.5)	1.9 (1.0–4.9)
**21**	Not active	263.0 (153.0–308.7)	1.1 (0.1–3.7)	80.0 (60.0–90.0)
**22**	42.2 (36.6–49.2)	30.2 (14.6–50.7)	35.2 (27.3–44.0)	16.6 (13.6–20.5)
**23**	92.9 (74.4–124.0)	45.1 (36.2–56.8)	1.4 (0.1–3.9)	17.6 (9.8–29.4)
**24**	Not active			
Dichlorvos	2.1 (1.5–3.8)	N/A	1.04 (0.1–1.9)	N/A
Cypermethrin	N/A	1.0 (0.1–2.0)	N/A	10.5 (0.1–20.0)

The concentrations evaluated ranged from 2.0 to 200.0 µL/L of air for the fumigation assays and from 1.0 to 200.0 µg/insect for the contact assays. The results are expressed as the mean of five replicates (n = 5) and two repetitions, along with their respective confidence intervals. The annexes present the slope and intercept values, with a confidence interval and a *p*-value < 0.05. N/A: Not applicable; LC_50_: Median lethal concentration; LD_50_: Median lethal dose.

## Data Availability

Data are contained within the article and Supplementary Materials.

## Supplementary Materials

The following supporting information can be downloaded at: https://www.mdpi.com/article/10.3390/ijms26073407/s1.

## Abbreviations

The following abbreviations are used in this manuscript:

δ	Chemical shift
δ_C_	Carbon shift
δ_H_	Hydrogen shift
°C	Degree Celsius
^13^C-NMR	Carbon Nuclear Magnetic Resonance
^1^H-NMR	Proton Nuclear Magnetic Resonance
approx	Approximately
APT	Attached Proton Test
BPO	Benzoyl peroxide
bs	broad singlet
CDCl_3_	Deuterated chloroform
d	Doublet
DCM	Dichloromethane
dd	Double doublet
ddd	double double doublet
EI	Electron impact
EO	Essential oil
EOs	Essentials oils
EtOAc	Ethyl Acetate
eV	Electron volt
Exp	Experimental
Exp LRI	Experimental Linear Retention Index
F_254_	Fluorescence indicator at a wavelength of 254 nm
FC	Flash Chromatography
GC–MS	Gas Chromatography–Mass Spectrometry
GCMS-TQ	Gas Chromatography–Mass Spectrometry with Triple Quadrupole
Hz	Hertz
ICA	Instituto Colombiano Agropecuario
J	Coupling constant
L	Liter
LC_50_	Median Lethal Concentration
LD_50_	Median Dose Concentration
LRI	Linear retention index
m	Multiplet
M	Molar
m/z	Mass/charge
mCPBA	Metachloroperbenzoic acid
MeOH	Methanol
mg	Milligram
MHz	Megahertz
min	Minutes
mL	Milliliter
mmol	Millimole
NBS	N-bromosuccinimide
NMR	Nuclear Magnetic Resonance
NOESY	Nuclear Overhauser Enhancement Spectroscopy
NOESY	Nuclear Overhauser Effect Spectroscopy
Pd/C	Palladium supported on carbon
PEG	Polyethylene glycol
ppm	Parts per million
PTFE	Polytetrafluoroethylene
q	Quartet
r.t.	Room temperature
Ref	Reference
Ref LRI	Reference linear retention index
RH	Relative Humidity
Rt	Time retention
s	Singlet
t	Triplet
td	triple doublet
tdd	triple double doublet
THF	Tetrahydrofuran
TLC	Thin-layer chromatography
UV	Ultraviolet
μL	Microliter
