# Supplementary material for: Insecticidal Activity of Monoterpenoids Against Sitophilus zeamais Motschulsky and Tribolium castaneum Herbst: Preliminary Structure–Activity Relationship Study
Source: Int J Mol Sci. 2025 Apr 5;26(7):3407. doi: 10.3390/ijms26073407 (PMC11989277; doi:10.3390/ijms26073407)
Supplement: Supplementary file 1 [file ijms-26-03407-s001.zip › ijms-3518448-supplementary.pdf]

International Journal of Molecular Sciences

MDPI

Supplementary Data

Table of Contents

1. Chromatographic Profile (TIC) of the GC-MS Analysis (orthogonal polarity) of the Essential Oil of A. graveolens (Figure S1, S2 and Table S1 and S2). 2
2. Chromatographic Profile (TIC) of the GC-MS Analysis (orthogonal polarity) of the Essential Oil of M. mollis (Figure S3 and S4, Table S3 and S4). 4
3. Chromatographic Profile (TIC) of the GC-MS Analysis (orthogonal polarity) of the Essential Oil of S. viminea (Figure S5 and S6, Table S5 and S6). 6
4. Chromatographic Profile (TIC) of the GC-MS Analysis (orthogonal polarity) of the Essential Oil of T. zypaquirensis (Figure S7 and S8, Table S7 and S8). 8
5. Physical and spectroscopic characterization of dill ether (1) (Figure S9 and S10, Table S9).. 10
6. Physical and spectroscopic characterization of Piperitone oxide (5) (Figure S11 and S12, Table S10).. 12
7. Physical and spectroscopic characterization of p-Menth-3-en-8-ol (8) (Figure S13 and S14, Table S11). 14
8. Physical and spectroscopic characterization of Dihydrotagetone (9) (Figure S15 and S16, Table S12).. 16
9. Physical and spectroscopic characterization of Myrcene epoxide (10) (Figure S17 and S18, Table S13). 18
10. Physical and spectroscopic characterization of Carvone epoxide (16) (Figure S19 and S20, Table S14). 20
11. Physical and spectroscopic characterization of Carvone hydrochloride (17) (Figure S21 and S22, Table S15). 22
12. Physical and spectroscopic characterization of Carvomenthone (18) (Figure S23 and S24, Table S16). 24
13. Physical and spectroscopic characterization of 6-Methyl-3-(1-methylethyl)-7-oxabicyclo [4.1.0] heptan-2-ol (19) (Figure S25 and S26, Table S17)................................... 26
14. Physical and spectroscopic characterization of 2,3-dihydroxy-6-isopropyl-3-methylcyclohexan-1-one (20) (Figure S27 and S28, Table S18). 28
15. Physical and spectroscopic characterization of Pulegone oxide (21a) (Figure S29 and S30, Table S19). 30
16. Physical and spectroscopic characterization of Pulegone hydrochloride (22) (Figure S31 and S32, Table S20). 32
17. Physical and spectroscopic characterization of p-menth-4-en-3-one (23) (Figure S33 and S34, Table S21).. 34
18. Physical and spectroscopic characterization of Pulegol (24) (Figure S35 and S36, Table S22).. 36
19. Statistical treatment of insecticidal activity of EOs and isolated, commercial, and synthesized compounds (Table S23 and S24)... 38

International Journal of
Molecular Sciences

MDPI

1. **Chromatographic Profile (TIC) of the GC-MS Analysis (orthogonal polarity) of the Essential Oil of *A. graveolens* (Figure S1, S2 and Table S1 and S2).**

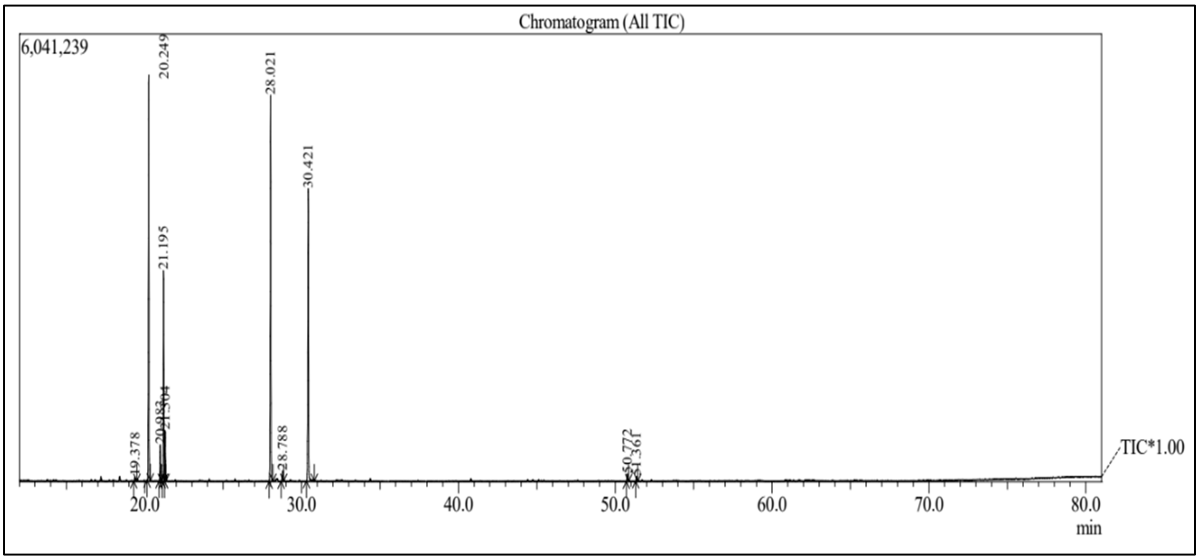

Figure S1. Chromatographic Profile (TIC) of the GC-MS Analysis (DB-5MS) of the Essential Oil of *A. graveolens*.

Table S1. Chemical Composition of the Essential Oil of *A. graveolens* (apolar column DB-5MS).

Compound	Rt	%Área	DB-5MS	
			RI Exp	RI Ref
β-Myrcene	19,378	0,4	989	986-994
α-Phellandrene	20,249	25,78	1012	1005-1032
o-Cimene	20,983	2,51	1028	1026-1036
D-Limonene	21,195	13,77	1031	1031-1039
β-Phellandrene	21,304	3,46	1037	1031-1053
Dill ether	28,021	28,56	1194	1183-1194
Carvone	30,421	23,67	1261	1242-1272

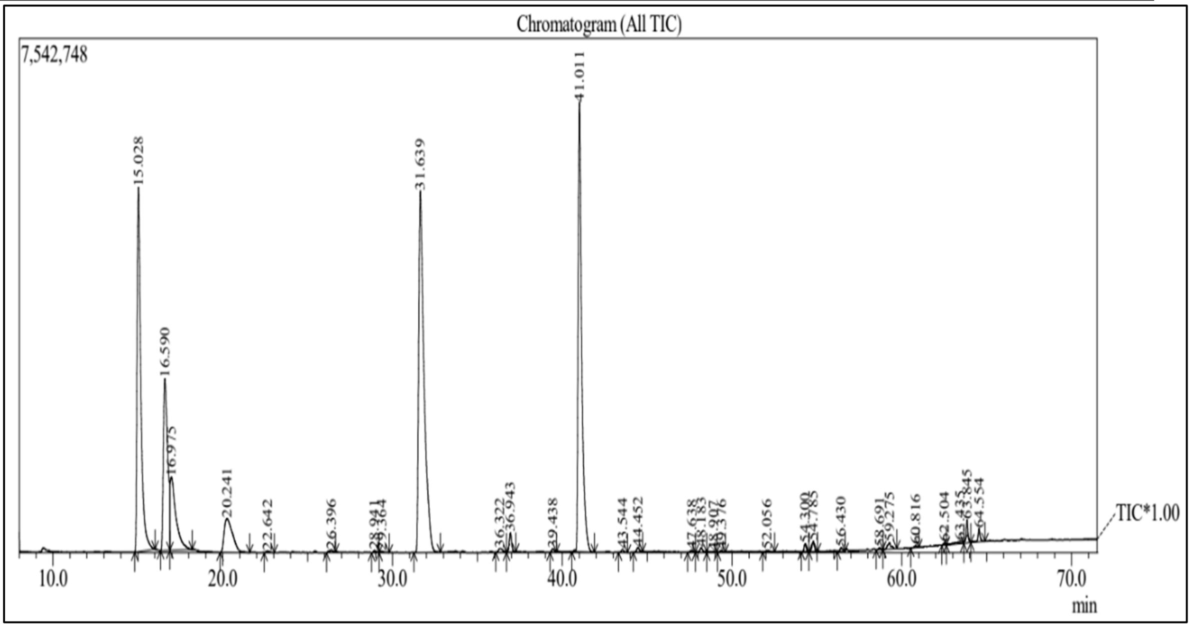

Figure S2. Chromatographic Profile (TIC) of the GC-MS Analysis (HP-INNOWax) of the Essential Oil of *A. graveolens*.

Table S2. Chemical Composition of the Essential Oil of *A. graveolens* (HP-INNOWax column).

Compound	Rt	%Área	HP-INNOWax	
			RI Exp	RI Ref
α-Phellandrene	15,028	20,51	1185	1166-1205
D-Limonene	16,59	11,26	1210	1198-1234
β-Phellandrene	20,241	4,23	1238	1189-1241
Dill ether	31,639	28,89	1489	1484-1529
Carvone	41,011	23,78	1717	1710-1735

International Journal of
Molecular Sciences

MDPI

2. Chromatographic Profile (TIC) of the GC-MS Analysis (orthogonal polarity) of the Essential Oil of *M. mollis* (Figure S3 and S4, Table S3 and S4).

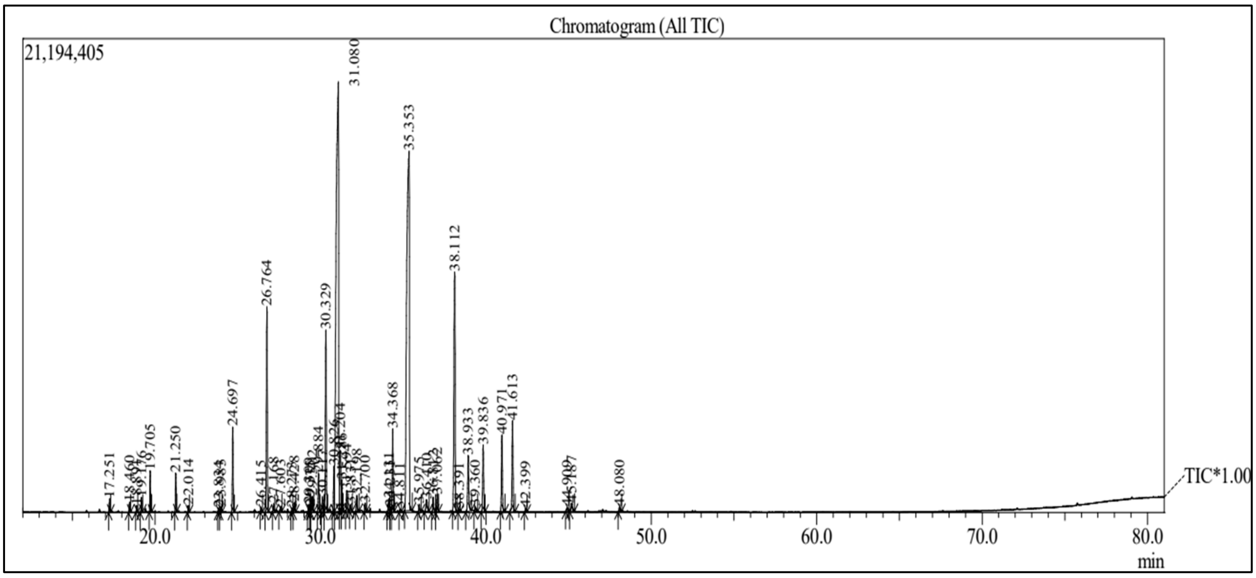

Figure S3. Chromatographic Profile (TIC) of the GC-MS Analysis (DB-5MS) of the Essential Oil of *M. mollis*.

Table S3. Chemical Composition of the Essential Oil of *M. mollis* (apolar column DB-5MS).

Compound	Rt	%Área	DB-5MS	
			RI Exp	RI Ref
α-Pinene	17,251	0,25	526	932-939
Isopropyl tyglate	18,460	0,20	966	959-976
Sabinene	18,990	0,13	979	960-980
β-Pinene	19,176	0,29	526	980-990
3-Octanol	19,705	0,77	996	991-995
D-Limonene	21,250	0,75	1031	1031-1039
Dihydrotagetone	22,014	0,13	1052	1047-1082
Linalool	23,985	0,12	1100	1096-1101
3-Octanyl acetate	24,697	1,79	1116	1102-1123
Menthone	26,764	5,10	1163	1148-1164
Perillyl ketone	29,884	0,88	1236	1230-1248
β-Citral	30,117	0,41	1241	1240-1242
Piperitone	30,329	4,63	1246	1243-12450
Piperitone oxide	31,080	31,70	1264	1230-1251
Isopulegyl acetate	31,204	2,00	1267	1277-1309
E-citral	31,286	0,62	1269	1267-1270
Carvacrol	32,198	0,43	1291	1286-1299
Piperitenone	34,368	2,03	1345	1340-1349
Pulegone	35,353	25,91	1250	1209-1237
α-Copaene	35,975	0,19	1385	1372-1389

β-Bourbunene	36,410	0,50	1396	1387-1401
α-Humulene	39,835	1,87	1469	1446-1464
Germacrene D	40,973	2,07	1493	1485-1519
Bicyclogermacrene	41,613	2,57	1508	1494-1517
14-hydroxy- Caryophyllene	45,187	0,29	1662	1660-1667

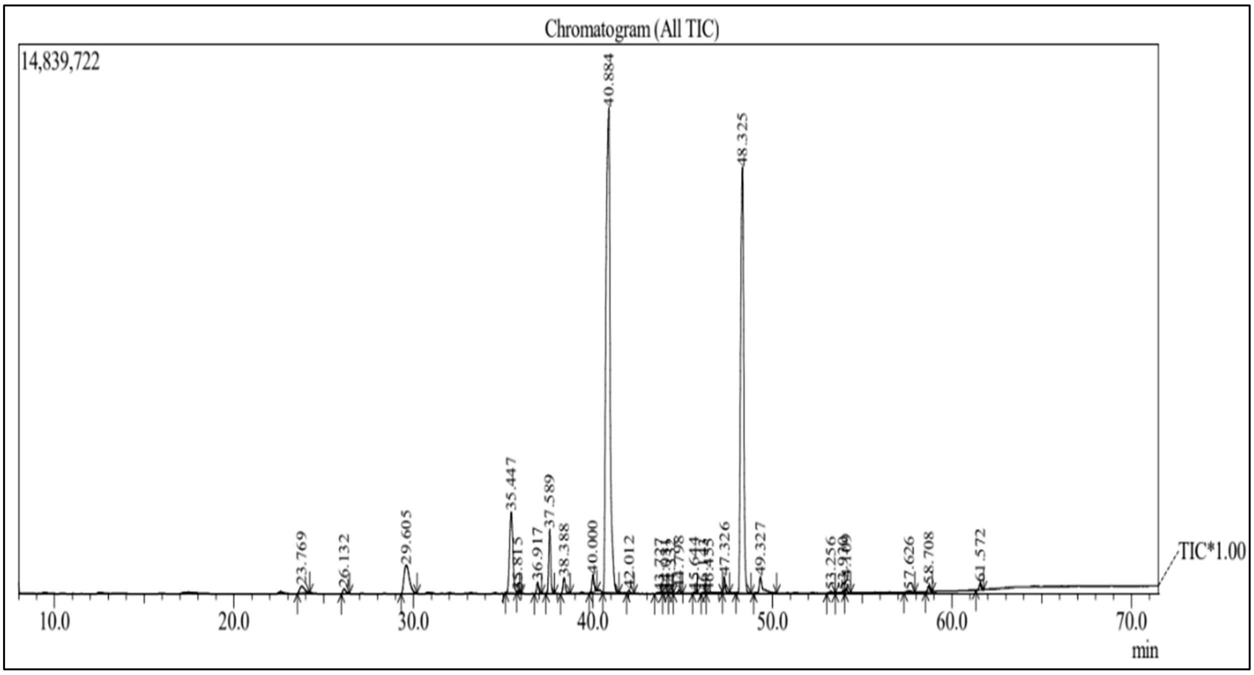

Figure S4. Chromatographic Profile (TIC) of the GC-MS Analysis (HP-INNOWax) of the Essential Oil of *M. mollis*.

Table S4. Chemical Composition of the Essential Oil of *M. mollis* (HP-INNOWax column).

Compound	Rt	%Área	HP-INNOWax	
			RI Exp	RI Ref
D-Limonene	23,769	0,83	1210	1198-1234
3-Octanol	26,132	0,35	1383	1368-1400
Dihydrotagetone	29,605	3,71	1285	1268-1319
Piperitenone	35,447	6,61	1710	1705-1739
Linalool	37,589	3,15	1579	1557-1581
α-Copaene	38,388	0,88	1504	1488-1520
Pulegone	40,884	47,86	1665	1661-1665
Piperitenone oxide	42,012	30,99	1712	1700-1722
α-Pinene	43,725	0,09	1028	1019-1030
Carvacrol	47,326	0,7	2217	2215-2219
3-octanyl acetate	48,325	0,14	1438	1424-1490
β- Caryophyllene	49,327	1,49	1627	1594-1657
E-citral	54,11	0,19	2038	1721-1737
Isopulegyl acetate	57,625	0,12	1695	1581-1608

International Journal of *Molecular Sciences*

MDPI

3. Chromatographic Profile (TIC) of the GC-MS Analysis (orthogonal polarity) of the Essential Oil of *S. viminea* (Figure S5 and S6, Table S5 and S6).

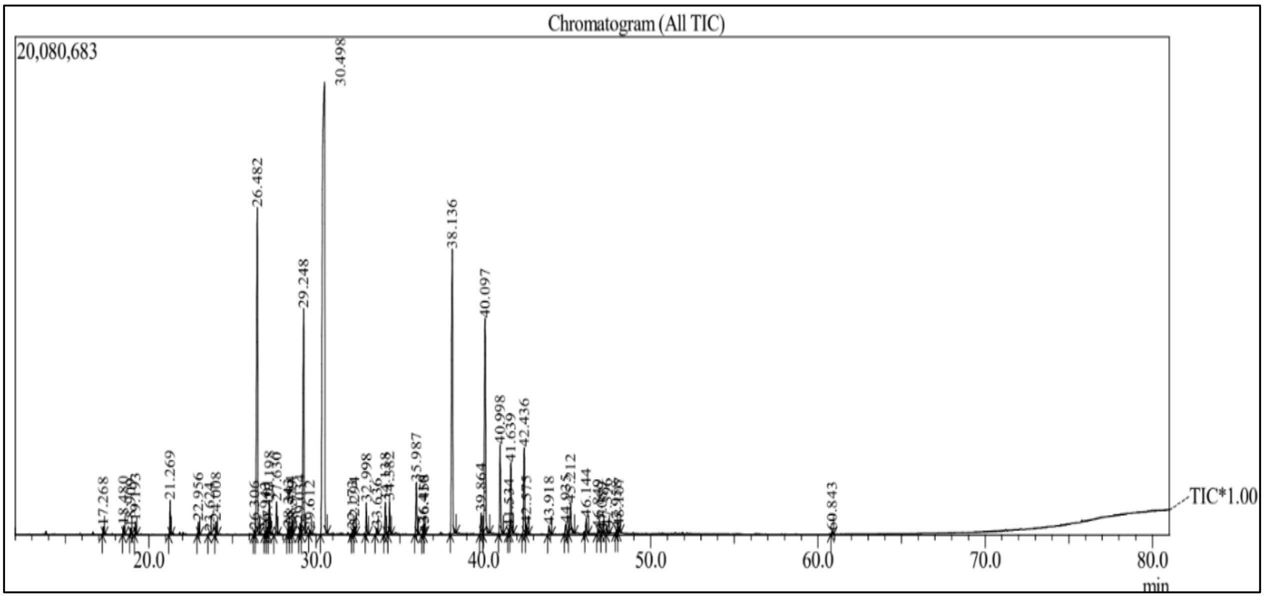

Figure S5. Chromatographic Profile (TIC) of the GC-MS Analysis (DB-5MS) of the Essential Oil of *S. viminea.*

Table S5. Chemical Composition of the Essential Oil of *S. viminea* (apolar column DB-5MS).

Compound	Rt	%Área	DB-5MS	
			RI Exp	RI Ref
α-Pinene	17,268	0,18	526	932-939
Isopropyl Tiglate	18,480	0,21	966	959-976
Sabinene	18,909	0,16	979	960-980
β-Pinene	19,193	0,3	526	980-990
D-Limonene	21,269	0,8	1031	1031-1039
p-Menth-3,8-dieno	22,956	0,3	1075	1070-1076
Linalool	24,008	0,33	1100	1096-1101
p-Menth-3-en-8-ol	26,482	11,83	1056	1147-1149
Isopulegone	26,482	1,13	1182	1157-1179
Trans-Pulegol	27,101	7,79	1220	1214-1221
Menthone	27,630	0,23	1163	1148-1164
Pulegone	30,498	37,4	1250	1209-1237
Dihydrocarvyl acetate	32,998	0,81	1311	1307-1344
Mirtenyl acetate	33,636	0,21	1327	1326-1332
Piperitenone	34,382	0,95	1345	1340-1349
α-Copaene	35,987	1,51	1385	1372-1389
β- Caryophyllene	38,136	11,33	1433	1418-1449
α-Humulene	39,864	0,73	1469	1446-1464
Alloaromadendrene	40,097	8,14	1474	1458-1478

International Journal of
Molecular Sciences
MDPI

Germacrene D	40,998	2,87	1493	1485-1519
Bicyclogermacrene	41,639	2,34	1508	1494-1517
δ-Cadinene	42,436	2,76	1531	1523-1531
Caryophyllene oxide	45,212	1,21	1668	1660-1682

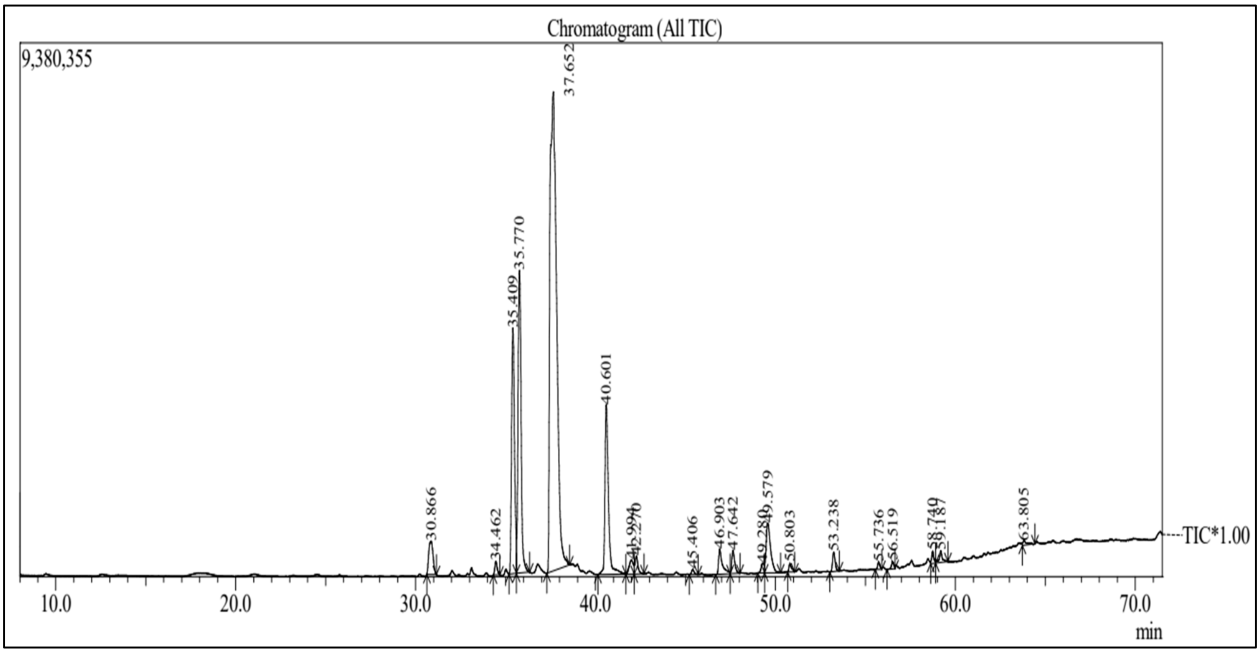

Figure S6. Chromatographic Profile (TIC) of the GC-MS Analysis (HP-INNOWax) of the Essential Oil of *S. viminea*.

Table S6. Chemical Composition of the Essential Oil of *S. viminea* (HP-INNOWax column).

Compound	Tr	%Área	HP-INNOWax	
			RI Exp	RI Ref
α-Copaene	30,866	2,28	1504	1488-1520
Isopulegone	34,460	0,50	1590	1582-1597
β- Caryophyllene	35,409	11,39	1627	1594-1657
p-Menth-3-en-8-ol	35,770	14,60	1613	1600-1621
Pulegone	37,650	50,44	1665	1661-1665
δ-Cadinene	41,995	0,93	1769	1764-1772
Dihydrocarvyl acetate	42,270	0,83	1678	1670-1685
Piperitenone	47,640	1,07	1710	1705-1739
Caryophyllene oxide	49,280	0,47	1969	1966-1989
Germacrene D	49,580	3,46	1722	1716-1724
Alloaromadendrene	53,240	0,77	1634	1616-1662
Bicyclogermacrene	55,735	0,27	1747	1736-1738

4. Chromatographic Profile (TIC) of the GC-MS Analysis (orthogonal polarity) of the Essential Oil of *T. zypaquirensis* (Figure S7 and S8, Table S7 and S8).

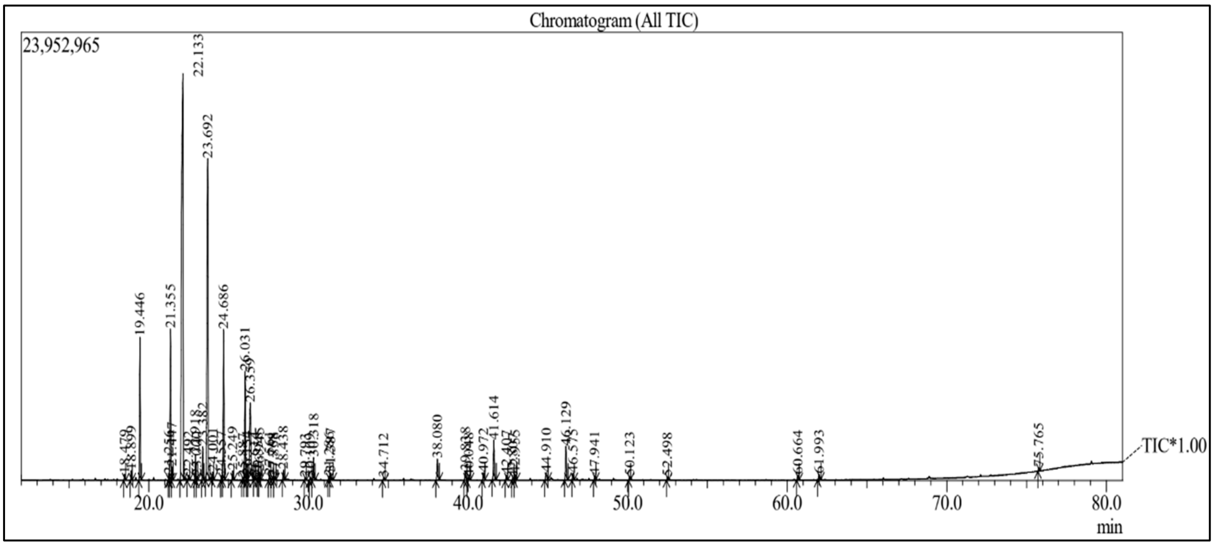

Figure S7. Chromatographic Profile (TIC) of the GC-MS Analysis (DB-5MS) of the Essential Oil of *T. zypaquirensis*.

Table S7. Chemical Composition of the Essential Oil of *T. zypaquirensis* (apolar column DB-5MS).

Compound	Rt	%Área	DB-5MS	
			RI Exp	RI Ref
Isopropyl tiglate	18,479	0,27	966	959-976
Sabinene	18,900	0.35	979	960-980
β-Myrcene	19,446	5,3	989	986-994
D-Limonene	21,256	0,2	1031	1031-1039
Eucalyptol	21,447	0,49	1038	1031-1039
Cis-β-ocimene	21,355	5.83	1036	1037-1043
Dihydrotagetone	22,133	35,39	1052	1047-1082
Ipsenone	23,382	1,26	1085	1083-1086
6,7-epoxymyrcene	23,692	19,64	1093	1092-1096
Linalool	24,001	0,29	1100	1096-1101
4-t-Pentylcyclohexene	24,686	6,49	1116	1100-1128
Z-Epoxyocimene	25,249	0,4	1128	1120-1132
E- Tagetone	26,030	4,74	1146	1144-1149
Z-Tagetone	26,359	6	1149	1147-1152
γ-Terpineol	28,438	0,44	1201	1195-1199
Pulegone	30,318	0,97	1250	1209-1237
Z-Isocitral	31,286	0,18	1169	1164-1184
Ccis-Linalool Oxide	31,387	0,37	1172	1170-1174
β-Caryophyllene	38,080	1,09	1433	1418-1449
α-Humulene	39,838	0,47	1469	1446-1464
Alloaromadendrene	40,048	0,18	1474	1458-1478

Germacrene D	40,970	0,4	1493	1485-1519
δ-Cadinene	42,407	0,25	1531	1523-1531
Bicyclogermacrene	41,615	1,97	1508	1494-1517
No identificated	46,129	1,55	1724	-

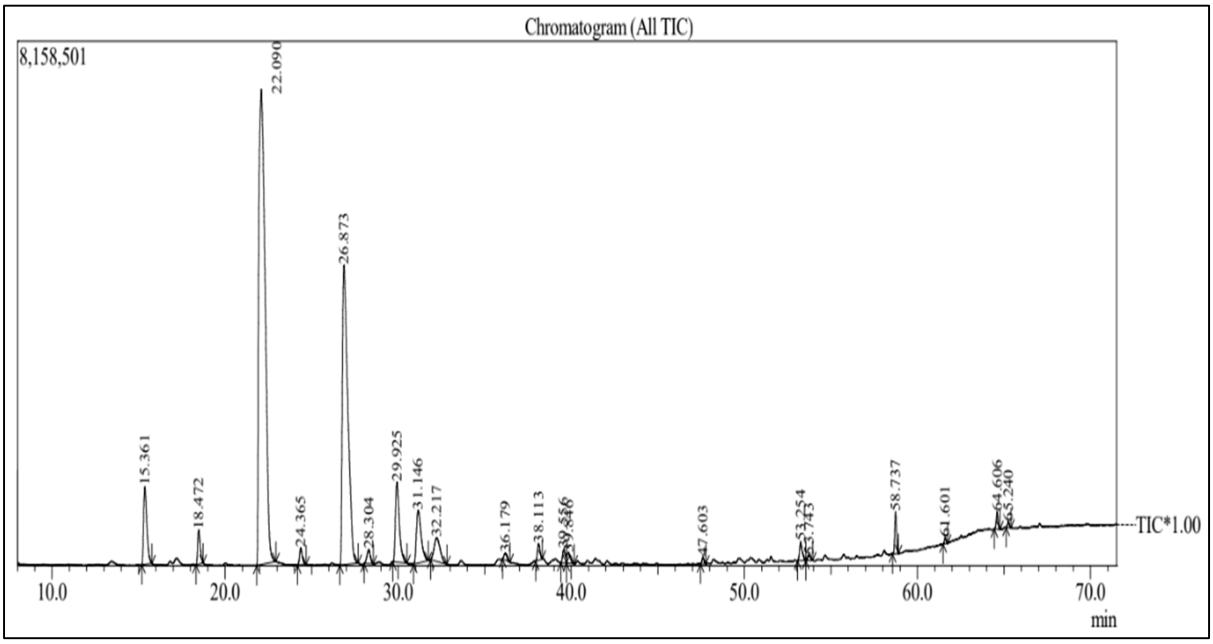

Figure S8. Chromatographic Profile (TIC) of the GC-MS Analysis (HP-INNOWax) of the Essential Oil of *T. zypaquirensis*.

Table S8. Chemical Composition of the Essential Oil of *T. zypaquirensis* (HP-INNOWax column).

Compound	Rt	%Área	HP-INNOWax	
			RI Exp	RI Ref
β-Myrcene	15,361	4,12	1147	1145-1187
Cis-β-ocimeno	18,472	1,44	1270	1242-1270
Dihydrotagetone	22,09	48,71	1285	1268-1319
Isopropyl tiglate	24,365	0,77	1233	1229-1238
6,7-epoxymyrcene	26,873	27,01	1399	1398-1415
Ipsenone	28,304	0,78	1440	1444
4-t-Pentylcyclohexene	29,925	5,19	1724	1720
E- Tagetone	31,146	4,13	1500	1501-1522
Z-Tagetone	32,217	2,33	1500	1500-1517
Linalool	36,18	0,32	1579	1557-1581
Pulegone	38,115	0,72	1665	1661-1665
β-Caryophyllene	39,556	0,59	1627	1594-1657
γ-Terpineol	39,846	0,59	1685	1684-1695
α-Humulene	39,846	0,59	1691	1660-1710
Alloaromadendrene	53,255	0,81	1634	1616-1662
Bicyclogermacrene	58,737	1,22	1747	1736-1738

International Journal of
Molecular Sciences
MDPI

5. Physical and spectroscopic characterization of dill ether (1) (Figure S9 and S10, Table S9).

Table S9. Spectroscopic characterization of dill ether (**1**).

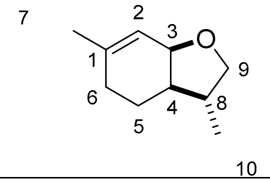	**Name**	Dill ether (**1**)
	**Physical state**	Colorless oil
	**Molecular formula**	$C_{10}H_{16}O$
	**Molecular weight**	152 g/mol
	**Specific rotation**	$[\alpha]_D^{25}$ = +27.7 (c = 1.0, $CHCl_3$)
^{1}H-NMR (400 MHz, $CDCl_3$): δ_H (ppm) 5.52 (dd, *J* = 3.0, 1.4 Hz, 1H, H-2), 4.25 (s, 1H, H-3), 4.07 (dd, *J* = 8.3, 7.3 Hz, 1H, H-9a), 3.31 (dd, *J* = 8.3, 7.0 Hz, 1H, H-9b), 2.08-1.98 (m, 1H, H-8), 1.96-1.84 (m, 2H, H-6), 1.76-1.64 (m, 2H, H-4 y H-5), 1.71 (s, 3H, H-7), 1.55-1.46 (m, 1H, H-5), 1.05 (d, *J* = 6.8 Hz, 3H, H-10).		
^{13}C-NMR (APT) (100 MHz, $CDCl_3$): δ_C (ppm) 139.0 (C-1), 121.0 (C-2), 75.2 (C-3), 74.1 (C-9), 44.0 (C-4), 38.1 (C-8), 28.3 (C-6), 24.2 (C-5), 23.9 (C-7), 17.8 (C-10).		
Bibliography: [75]		

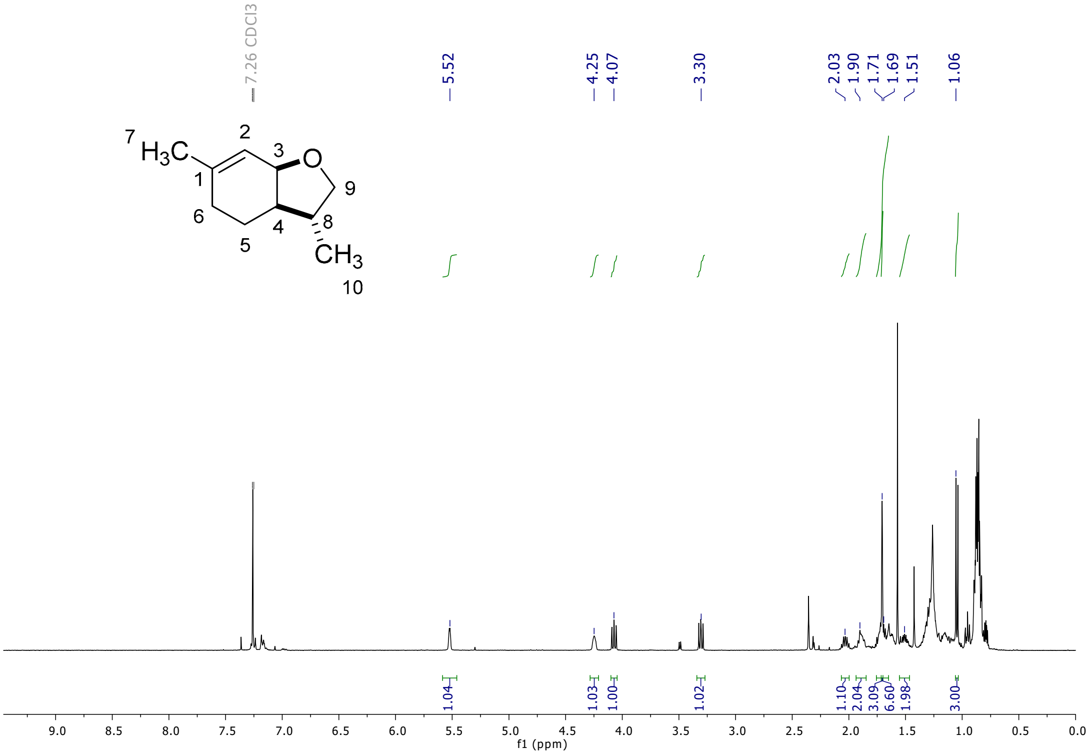

Figure S9. ^{1}H -NMR spectra of Dill ether (**1**).

International Journal of Molecular Sciences

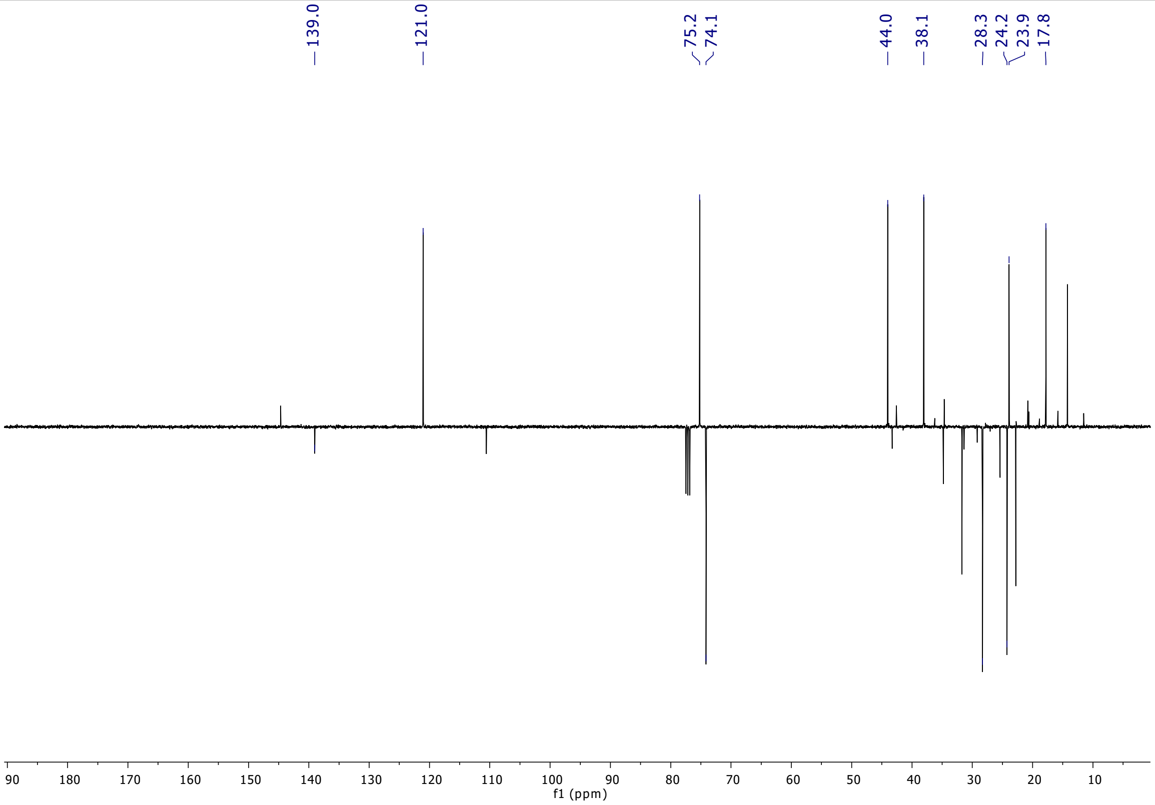

Figure S10. ^{13}C-NMR (APT) spectra of Dill ether (**1**).

International Journal of
Molecular Sciences

6. Physical and spectroscopic characterization of Piperitone oxide (5) (Figure S11 and S12, Table S10).

Table S10. Spectroscopic characterization of Piperitone oxide (**5**).

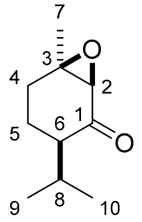	**Name**	Piperitone oxide (**5**)
	**Physical state**	Colorless oil
	**Molecular formula**	$C_{10}H_{16}O$
	**Molecular weight**	152 g/mol
	**Specific rotation**	$[\alpha]_D^{25}$ = -120.0 (c = 1.0, $CHCl_3$)
^{1}H-NMR (400 MHz, $CDCl_3$): δ_H (ppm) 3.06 (s, 1H, H-2), 2.39-2.30 (m, 1H, H-8), 2.17-2.11 (m, 1H, H-6), 1.88-1.69 (m, 4H, H-4, H-5), 1.42 (s, 3H, H-7), 0.90 (d, *J* = 7.0 Hz, 3H, H-9), 0.81 (d, *J* = 6.9 Hz, 3H, H-10).		
^{13}C-NMR (APT) (100 MHz, $CDCl_3$): δ_C (ppm) 208.8 (C-1), 62.6 (C-2), 61.7 (C-3), 52.2 (C-6), 29.0 (C-8), 28.6 (C-4), 22.0 (C-7), 20.2 (C-9), 18.3 (C-10), 16.9 (C-5).		
Bibliography: [76]		

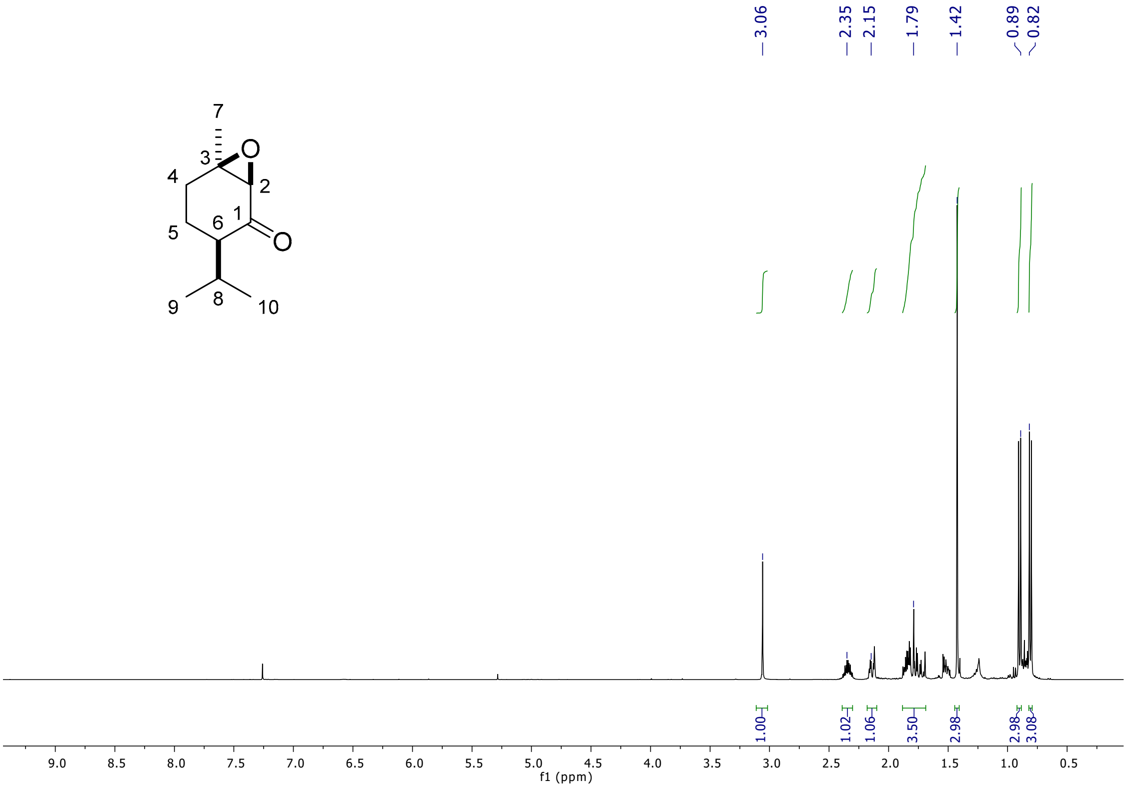

Figure S11. ^{1}H -NMR spectra of piperitone oxide (**5**).

International Journal of
Molecular Sciences

MDPI

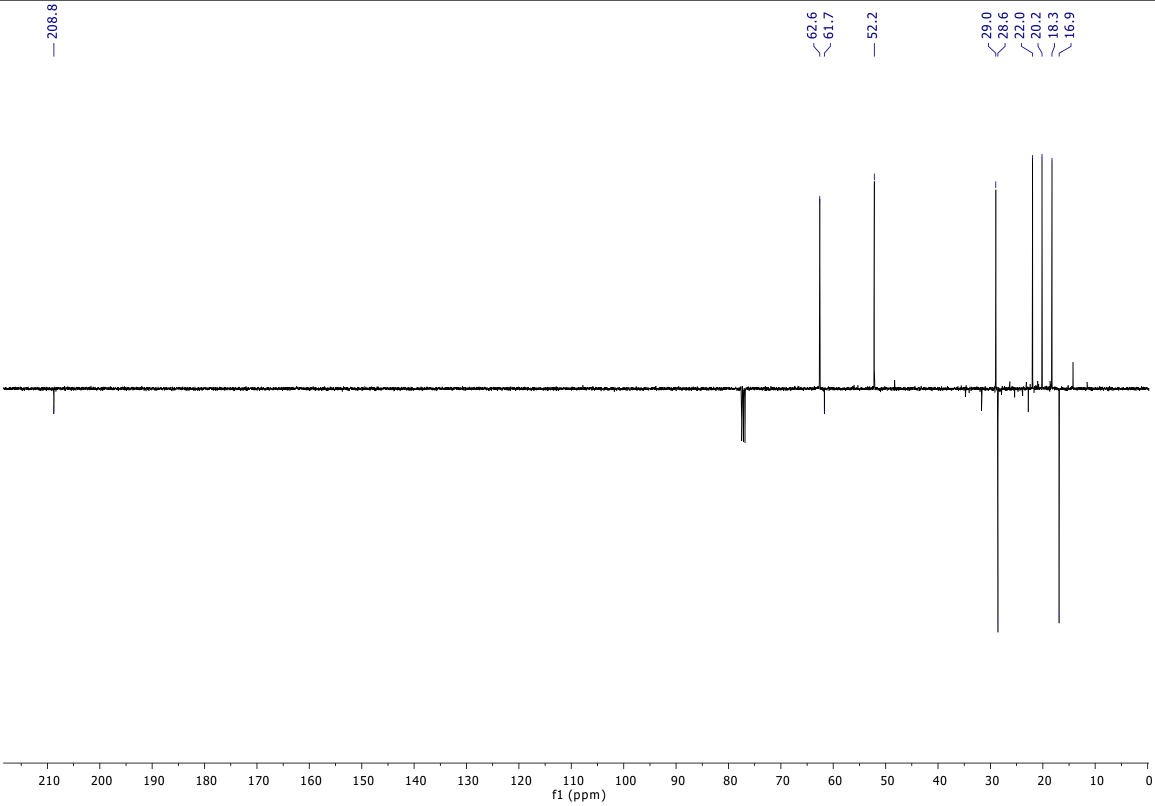

Figure S12. ^{13}C-NMR (APT) spectra of piperitone oxide (**5**).

7. Physical and spectroscopic characterization of p-Menth-3-en-8-ol (8) (Figure S13 and S14, Table S11).

Table S11. Spectroscopic characterization of p-Menth-3-en-8-ol (**8**).

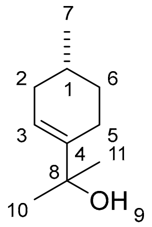	**Name**	p-Menth-3-en-8-ol (**8**)
	**Physical state**	Colorless oil
	**Molecular formula**	$C_{10}H_{16}O$
	**Molecular weight**	152 g/mol
	**Specific rotation**	$[\alpha]_D^{25}$ = +2.1 (c = 1.0, $CHCl_3$)
^{1}H-NMR (400 MHz, $CDCl_3$): δ_H (ppm) 5.73-5.65 (m, 1H, H-3), 2.12-2.04 (m, 2H, H-5), 1.77-1.69 (m, 2H, H-2), 1.62-1.51 (m, 2H, H-6), 1.30 (s, 3H, H-9), 1.29 (s, 3H, H-10), 1.22-1.16 (m, 1H, H-1), 0.93 (d, *J* = 6.3 Hz, 3H, H-7).		
^{13}C-NMR (APT) (100 MHz, $CDCl_3$): δ_C (ppm) 143.6 (C-4), 118.6 (C-3), 73.0 (C-8), 33.9 (C-2), 31.5 (C-6), 29.0 (C-9 y C-10), 28.4 (C-1), 24.6 (C-5), 21.8 (C-7).		
Bibliography: [77]		

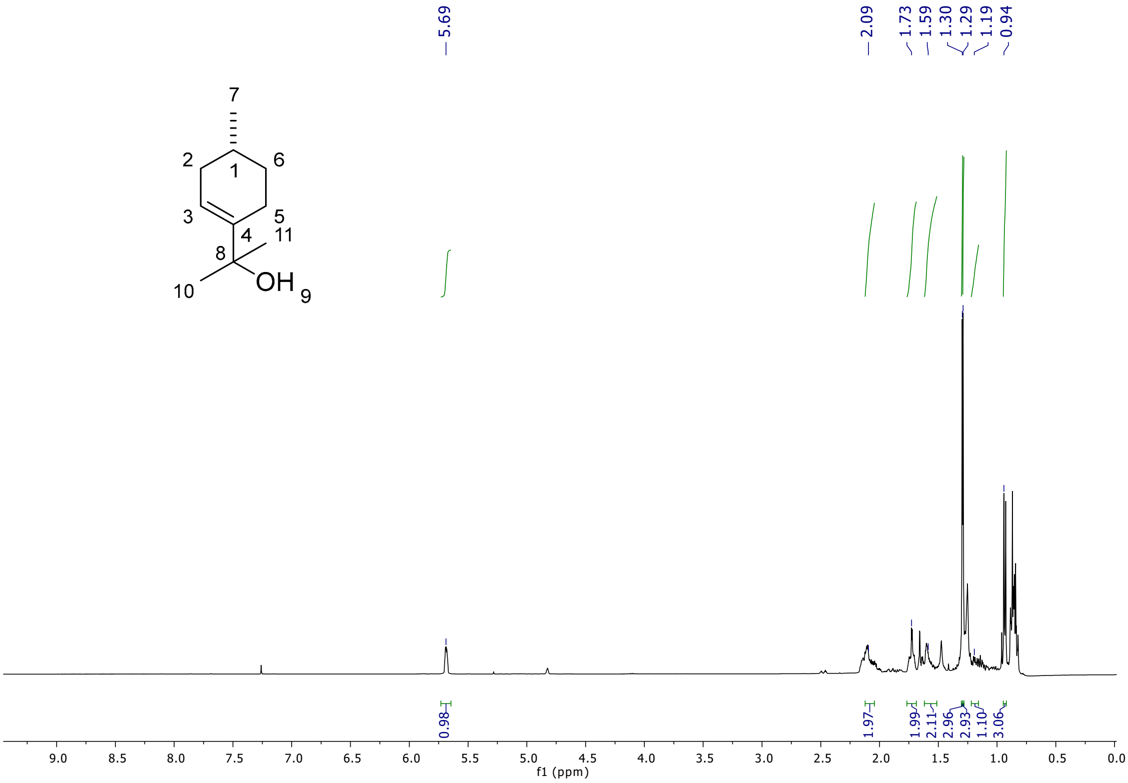

Figure S13. ^{1}H -NMR spectra of p-menth-3-en-8-ol (**8**).

International Journal of
Molecular Sciences

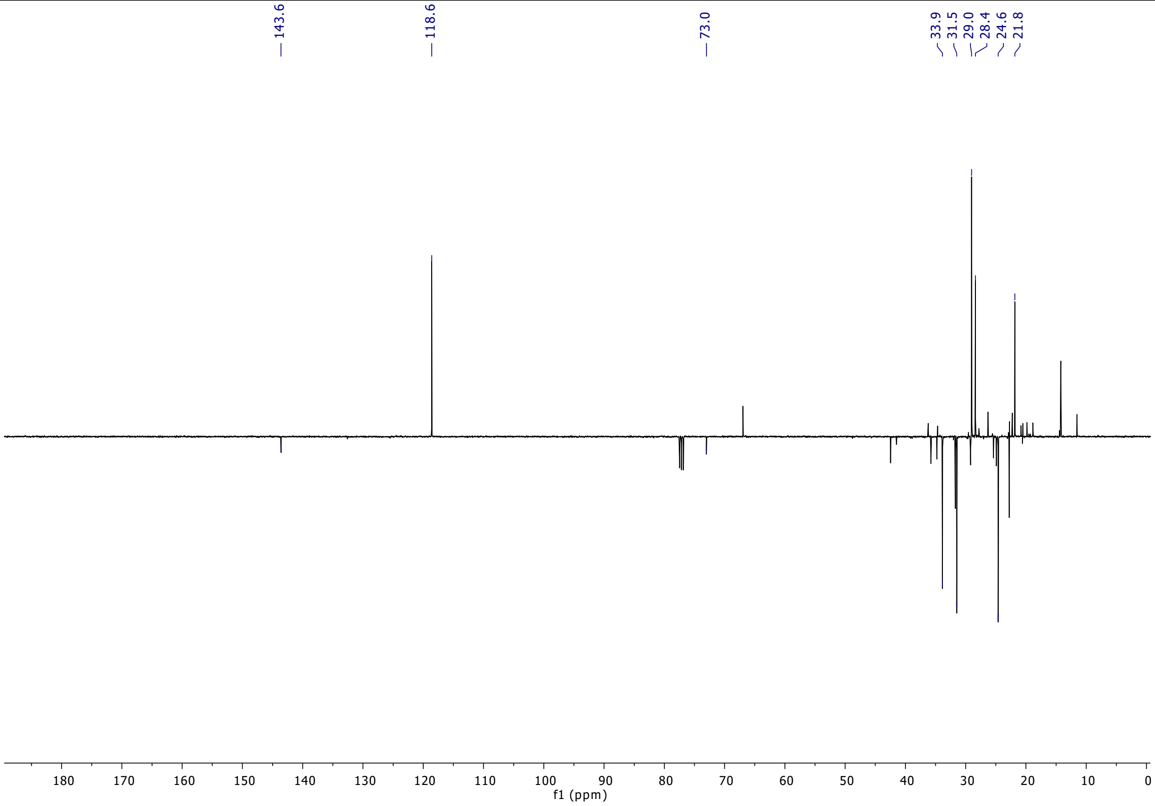

Figure S14. ^{13}C-NMR (APT) spectra of p-menth-3-en-8-ol (**8**).

International Journal of
Molecular Sciences

MDPI

8. Physical and spectroscopic characterization of Dihydrotagetone (9) (Figure S15 and S16, Table S12).

Table S12. Spectroscopic characterization of Dihydrotagetone (**9**).

<table>
<tr><td rowspan="5">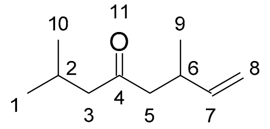</td><td>Name</td><td>Dihydrotagetone (9)</td></tr>
<tr><td>Physical state</td><td>Pale-yellow oil</td></tr>
<tr><td>Molecular formula</td><td>$C_{10}H_{16}O$</td></tr>
<tr><td>Molecular weight</td><td>152 g/mol</td></tr>
<tr><td>Specific rotation</td><td>$[\alpha]_D^{25}$ = +3.0 (c = 1.0, $CHCl_3$)</td></tr>
<tr><td colspan="3">^{1}H-NMR (400 MHz, $CDCl_3$): δ_H (ppm) 5.80-5.68 (m, 1H, H-7), 5.03-4.88 (m, 2H, H-8), 2.78-2.66 (m, 1H, H-6), 2.47-2.27 (m, 2H, H-5), 2.25 (d, J = 6.9 Hz, 2H, H-3), 2.19-2.06 (m, 1H, H-2), 1.00 (d, J = 6.8 Hz, 3H, H-9), 0.90 (d, J = 1.1 Hz, 3H, H-1), 0.89 (d, J = 1.0 Hz, 3H, H-10).</td></tr>
<tr><td colspan="3">^{13}C-NMR (APT) (100 MHz, $CDCl_3$): δ_C (ppm) 210.0 (C-4), 143.0 (C-7), 113.0 (C-8), 52.5 (C-3), 49.9 (C-5), 33.2 (C-6), 24.5 (C-2), 22.6 (C-1), 22.6 (C-10), 19.8 (C-9).</td></tr>
<tr><td colspan="3">Bibliography: [78]</td></tr>
</table>

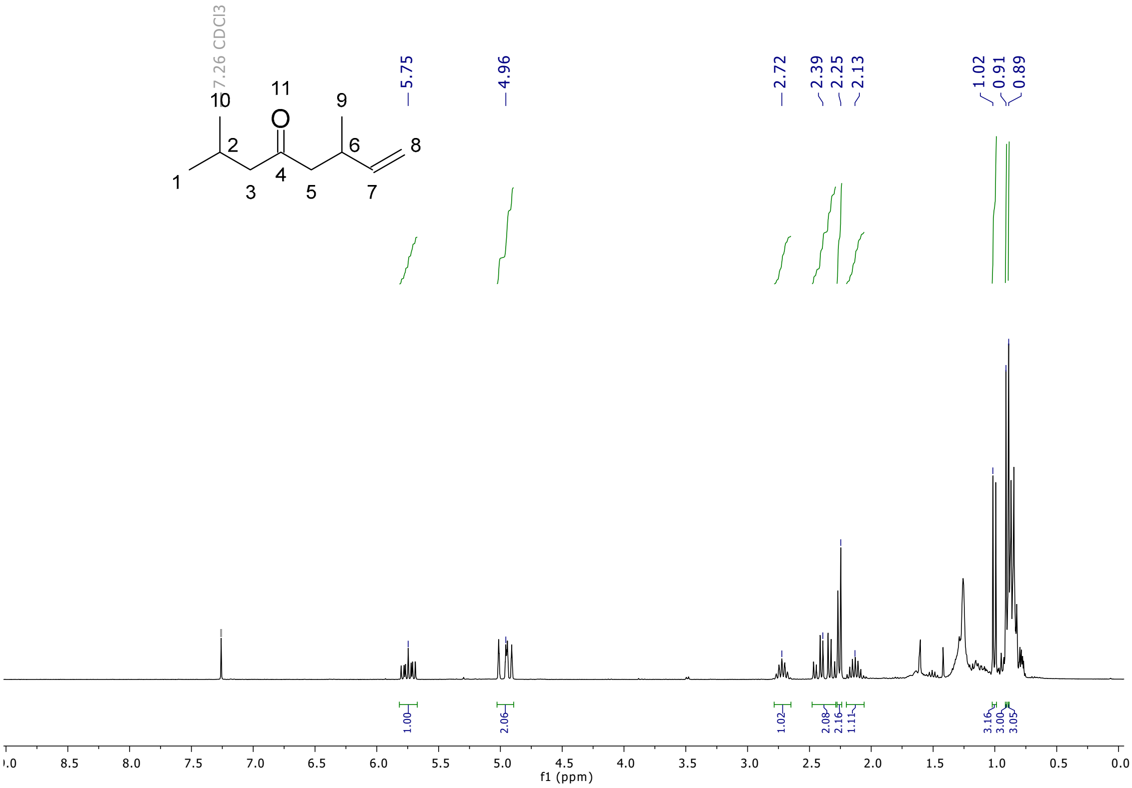

Figure S15. ^{1}H -NMR spectra of dihydrotagetone (**9**).

International Journal of Molecular Sciences
MDPI

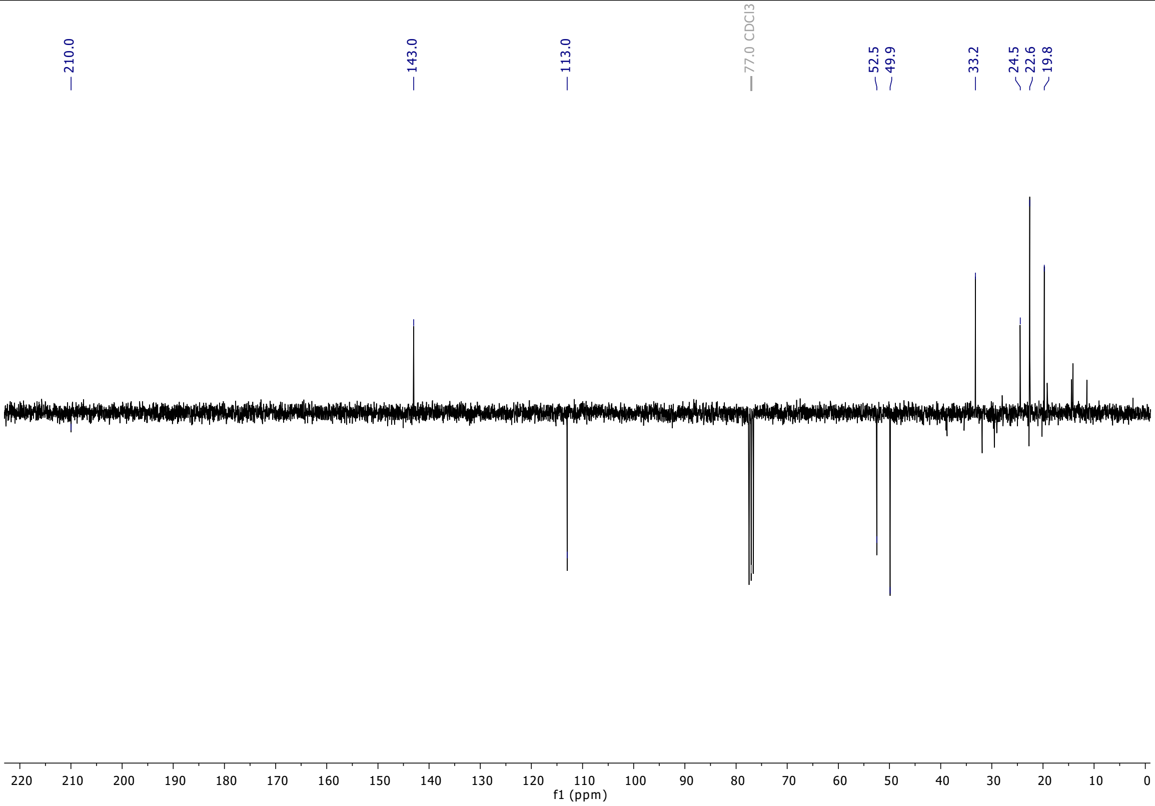

Figure S16. ^{13}C-NMR (APT) spectra of dihydrotagetone (**9**).

9. Physical and spectroscopic characterization of Myrcene epoxide (10) (Figure S17 and S18, Table S13).

Table S13. Spectroscopic characterization of Myrcene epoxide (**10**).

<table>
<tr><td rowspan="5">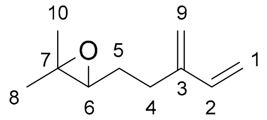</td><td>Name</td><td>Myrcene epoxide (10)</td></tr>
<tr><td>Physical state</td><td>Colorless oil</td></tr>
<tr><td>Molecular formula</td><td>$C_{10}H_{16}O$</td></tr>
<tr><td>Molecular weight</td><td>152 g/mol</td></tr>
<tr><td>Specific rotation</td><td>$[\alpha]_D^{25}$ = +20.0 (c = 1.0, $CHCl_3$)</td></tr>
<tr><td colspan="3">^{1}H-NMR (400 MHz, $CDCl_3$): δ_H (ppm) 6.37 (dd, J = 17.6, 10.8 Hz, 1H, H-2), 5.23 (d, J = 17.6 Hz, 1H, H-1), 5.07 (d, J = 11.0 Hz, 1H, H-1), 5.04 (s, 1H, H-9), 5.02 (s, 1H, H-9), 2.75 (t, J = 6.3 Hz, 1H, H-6), 2.47-2.38 (m, 1H, H-5), 2.35-2.26 (m, 1H, H-5), 1.76-1.68 (m, 2H, H-4), 1.30 (s, 3H, H-10), 1.25 (s, 3H, H-8).</td></tr>
<tr><td colspan="3">^{13}C-NMR (APT) (100 MHz, $CDCl_3$): δ_C (ppm) 145.5 (C-3), 138.7 (C-2), 116.3 (C-9), 113.6 (C-1), 64.2 (C-6), 58.6 (C-7), 28.2 (C-4), 27.7 (C-5), 25.0 (C-10), 18.9 (C-8).</td></tr>
<tr><td colspan="3">Bibliography: [79]</td></tr>
</table>

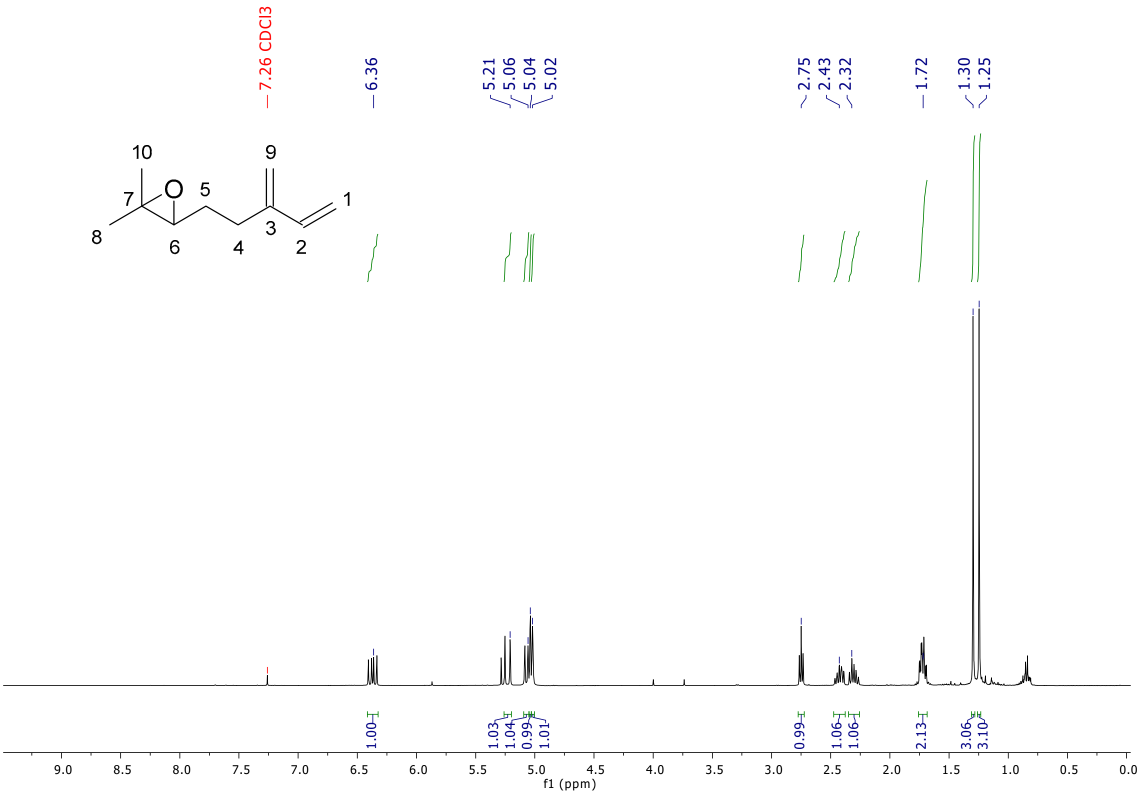

Figure S17. ^{1}H -NMR spectra of myrcene epoxide (**10**).

International Journal of Molecular Sciences MDPI

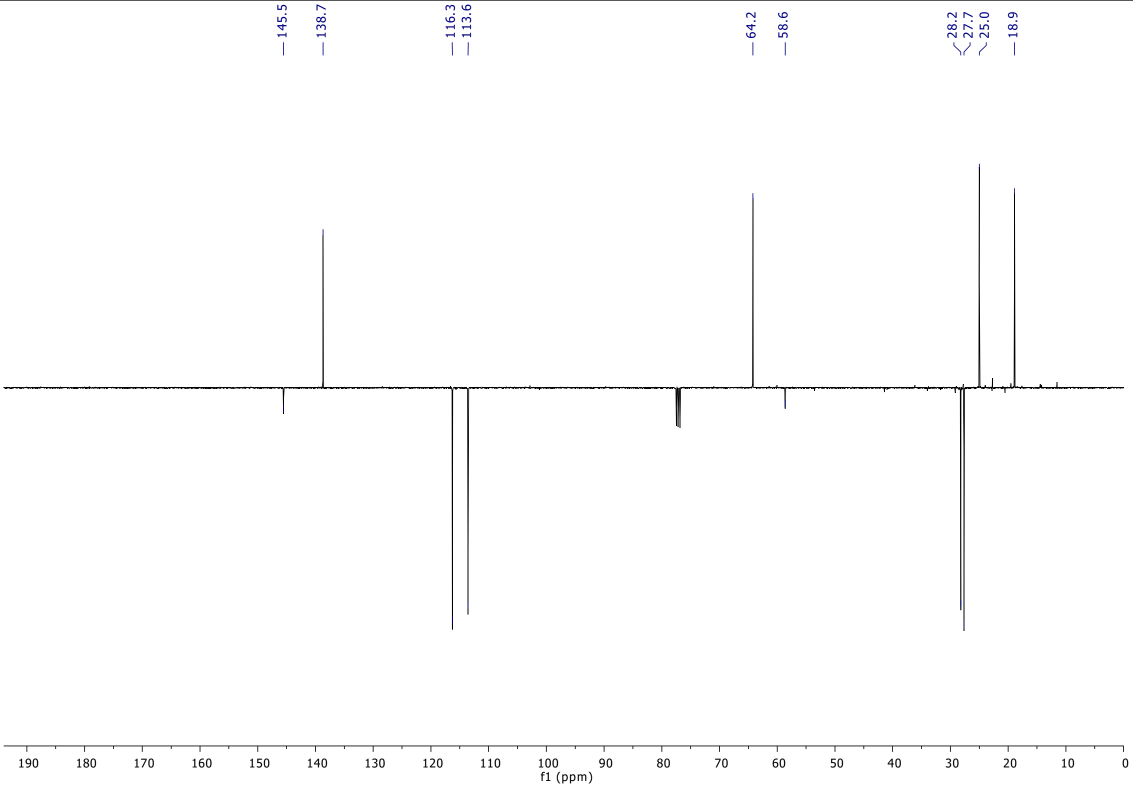

Figure S18. ^{13}C-NMR (APT) spectra of myrcene epoxide (**10**).

10. Physical and spectroscopic characterization of Carvone epoxide (16) (Figure S19 and S20, Table S14).

Table S14. Spectroscopic characterization of Carvone epoxide (**16**).

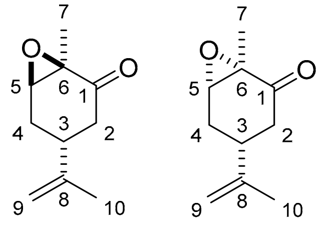 16a 16b	**Name**	Carvone epoxide (**16**)
	**Physical state**	Colorless oil
	**Molecular formula**	$C_{10}H_{14}O_2$
	**Molecular weight**	166 g/mol
	**Specific rotation**	$[\alpha]_D^{25}$ = +30.9 (c = 1.0, $CHCl_3$)
^{1}H-NMR ($CDCl_3$, 400 MHz): δ_H (ppm) 4.78 (t, *J* = 1.5 Hz, 1H, H-9), 4.72-4.69 (m, 1H, H-9), 3.43 (dd, *J* = 3.2, 1.2 Hz, 1H, H-5), 2.76-2.65 (m, 1H, H-3), 2.57 (ddd, *J* = 17.6, 4.7, 1.4 Hz, 1H, H-2), 2.42-2.31 (m, 1H, H-2), 2.01 (dd, *J* = 17.6, 11.6 Hz, 1H, H-4), 1.89 (ddd, *J* = 14.8, 11.1, 1.2 Hz, 1H, H-4), 1.70 (s, *J* = 1.1 Hz, 3H, H-10), 1.40 (s, 3H, H-7).		
^{13}C-NMR (APT) ($CDCl_3$, 100 MHz): δ_C (ppm) 205.6 (C-1), 146.5 (C-8), 110.6 (C-9), 61.5 (C-5), 58.9 (C-6), 41.9 (C-2), 35.2 (C-3), 28.8 (C-4), 20.7 (C-10), 15.4 (C-7).		
Bibliography: [54]		

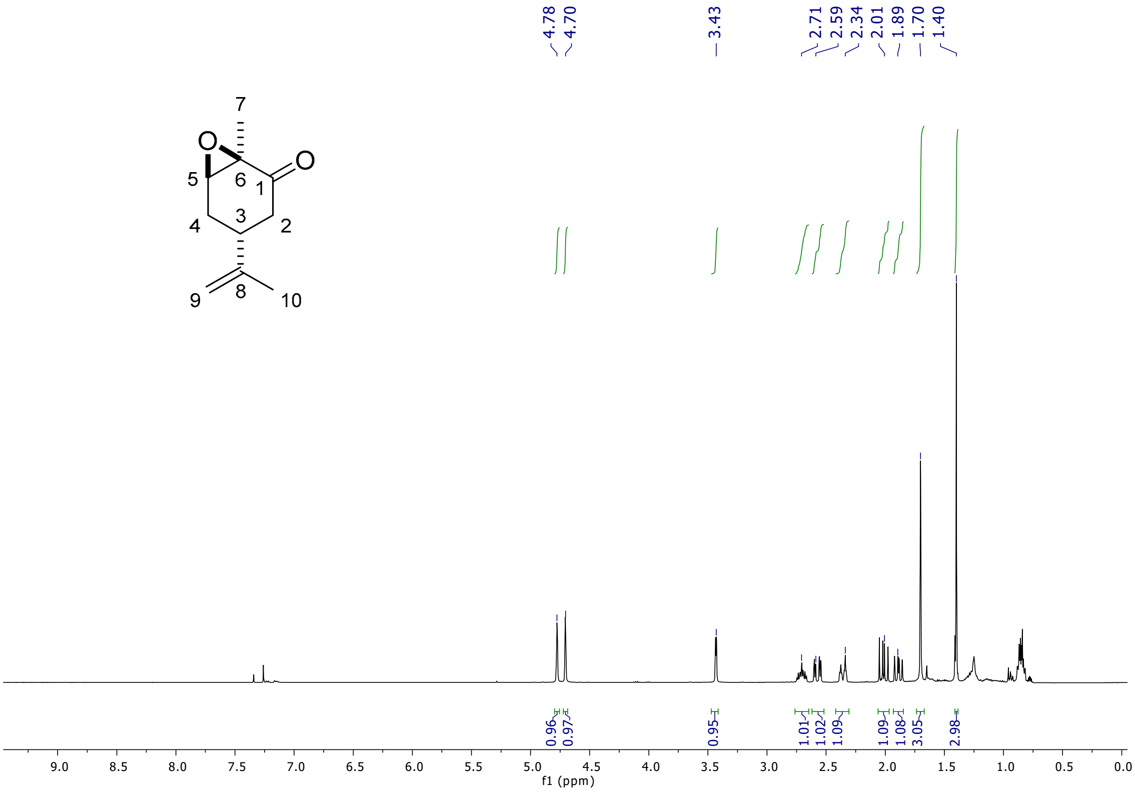

Figure S19. ^{1}H -NMR spectra of carvone epoxide (**16**).

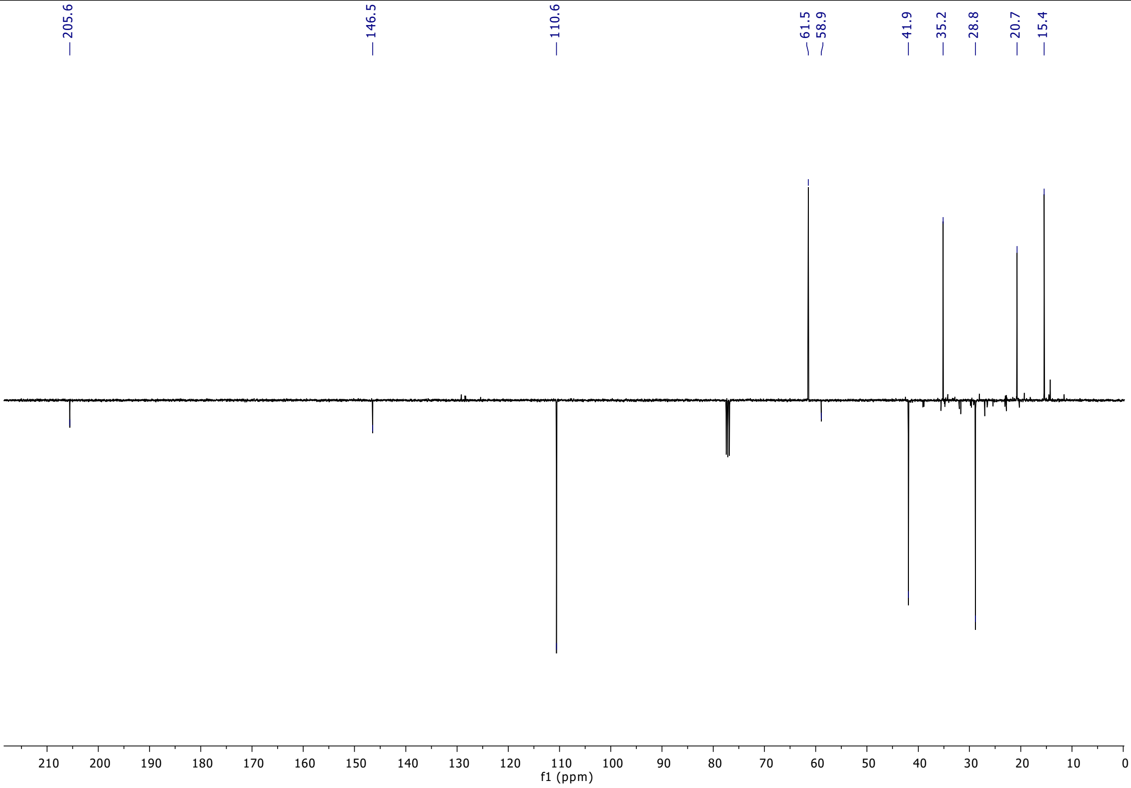

Figure S20. ^{13}C-NMR (APT) spectra of carvone epoxide (**16**).

International Journal of *Molecular Sciences* MDPI

11. Physical and spectroscopic characterization of Carvone hydrochloride (17) (Figure S21 and S22, Table S15).

Table S15. Spectroscopic characterization of Carvone hydrochloride (**17**).

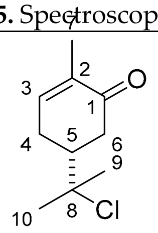	**Name**	Carvone hydrochloride (**17**)
	**Physical state**	Yellow oil.
	**Molecular formula**	$C_{10}H_{15}ClO$
	**Molecular weight**	186 g/mol
	**Specific rotation**	$[\alpha]_D^{25}$ = -27.5 (c = 1.0, $CHCl_3$)
^{1}H-NMR ($CDCl_3$, 400 MHz): δ_H (ppm) 6.77-6.73 (m, 1H, H-3), 2.70 (ddd, *J* = 16.0, 3.5, 1.8 Hz, 1H, H-4), 2.59-2.50 (m, 1H, H-4), 2.43-2.32 (m, 2H, H-6), 2.28-2.19 (m, 1H, H-5), 1.78 (brs, 3H, H-7), 1.59 (s, 3H, H-10), 1.57 (s, 3H, H-10).		
^{13}C-NMR (APT) ($CDCl_3$, 100 MHz): δ_C (ppm) 199.4 (C-1), 144.5 (C-3), 135.4 (C-2), 72.2 (C-8), 47.4 (C-5), 40.2 (C-6), 30.7 (C-10), 30.4 (C-9), 28.0 (C-4), 15.7 (C-7).		
Bibliography: [80]		

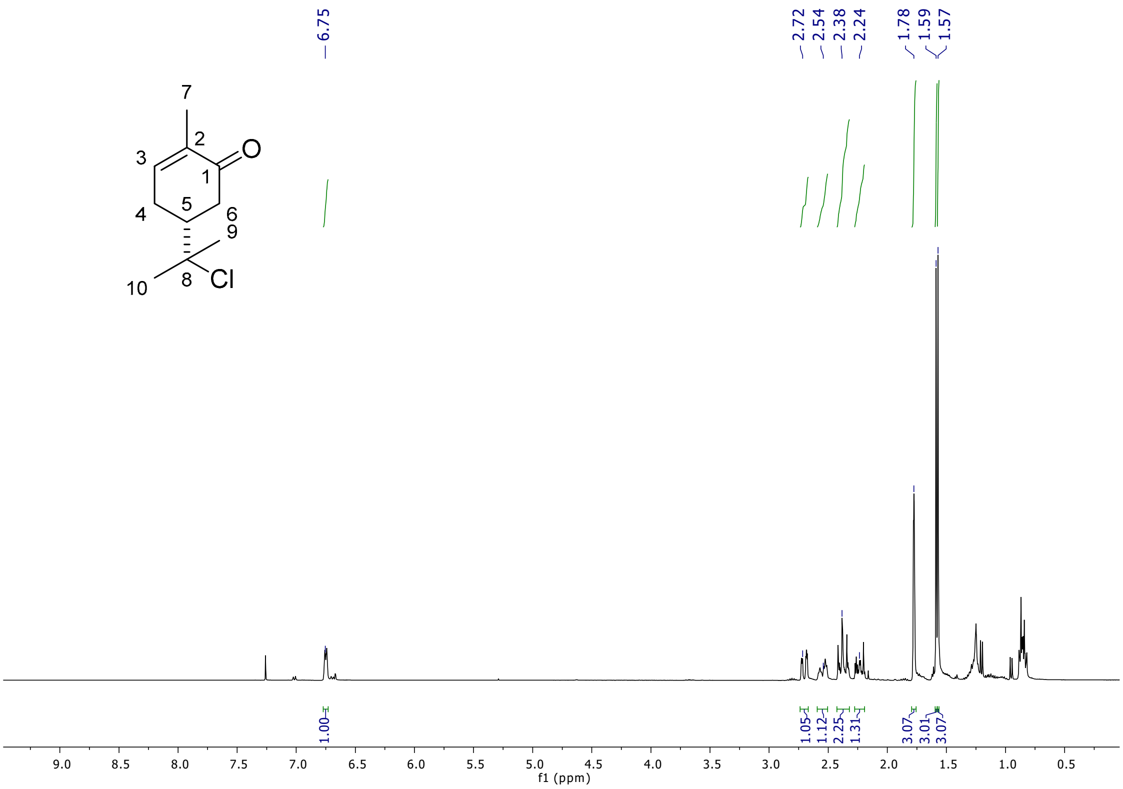

Figure S21. ^{1}H -NMR spectra of carvone hydrochloride (**17**).

International Journal of
Molecular Sciences

MDPI

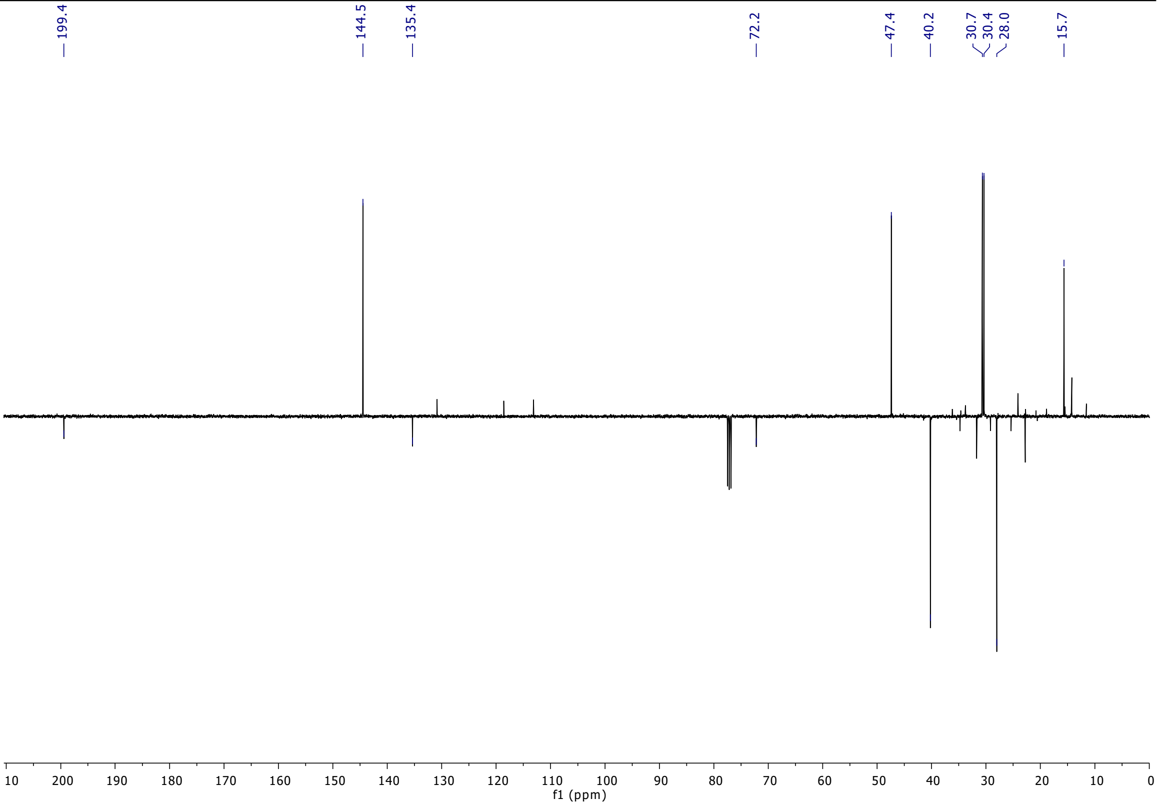

Figure S22. ^{13}C-NMR (APT) spectra of carvone hydrochloride (**17**).

International Journal of *Molecular Sciences* MDPI

12. Physical and spectroscopic characterization of Carvomenthone (18) (Figure S23 and S24, Table S16).

Table S16. Spectroscopic characterization of Carvomenthone (**18**).

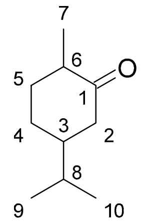	**Name**	Carvomenthone (**18**)
	**Physical state**	Colorless oil
	**Molecular formula**	$C_{10}H_{18}O$
	**Molecular weight**	154 g/mol
	**Specific rotation**	$[\alpha]_D^{25} = 0$ (c = 1.0, $CHCl_3$)
^{1}H-NMR ($CDCl_3$, 400 MHz): δ_H (ppm) 2.40-2.27 (m, 1H, H-6), 2.13-2.00 (m, 2H, H-2), 1.90-1.78 (m, 1H, H-3), 1.74-1.40 (m, 3H, H-5 y H-8), 1.38-1.26 (m, 2H, H-4), 1.00 (d, *J* = 6.5 Hz, 3H, H-7), 0.89 (d, *J* = 4.2 Hz, 3H, H-9), 0.87 (d, *J* = 4.4 Hz, 3H, H-10).		
^{13}C-NMR (APT) ($CDCl_3$, 100 MHz): δ_C (ppm) 213.8 (C-1), 46.7 (C-6), 45.5 (C-3), 45.0 (C-2), 35.2 (C-5), 32.9 (C-8), 29.0 (C-4), 19.7 (C-9), 19.5 (C-10), 14.5 (C-7).		
Bibliography: [63]		

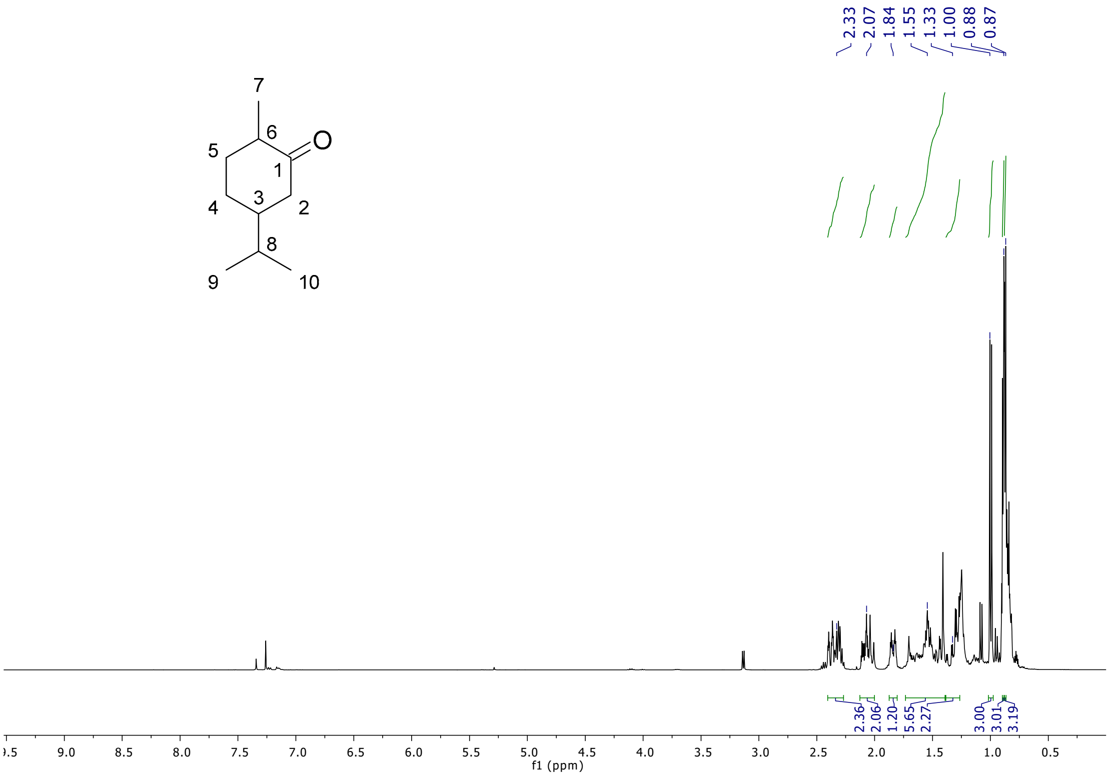

Figure S23. ^{1}H -NMR spectra of carvomenthone (**18**).

International Journal of
Molecular Sciences

MDPI

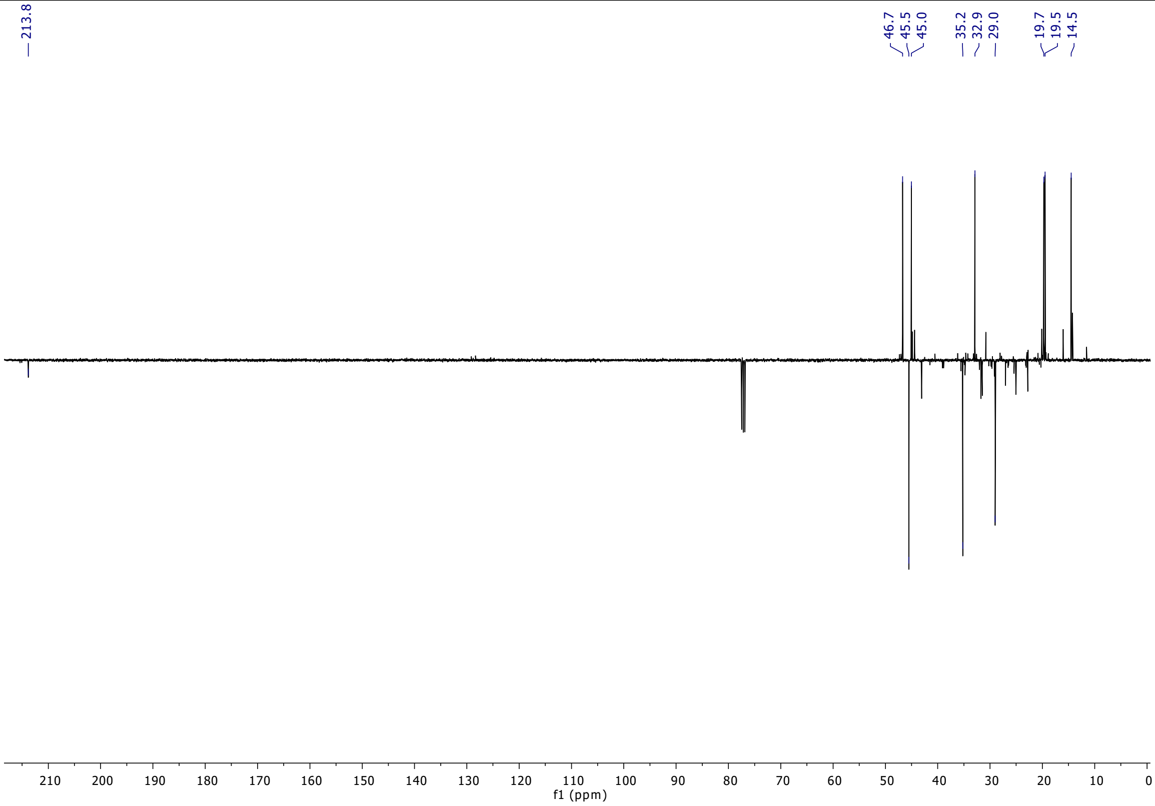

Figure S24. ^{13}C-NMR (APT) spectra of carvomenthone (**18**).

International Journal of *Molecular Sciences*

MDPI

13. Physical and spectroscopic characterization of 6-Methyl-3-(1-methylethyl)-7-oxabicyclo [4.1.0] heptan-2-ol (19) (Figure S25 and S26, Table S17).

Table S17. Spectroscopic characterization of 6-Methyl-3-(1-methylethyl)-7-oxabicyclo [4.1.0] heptan-2-ol (**19**).

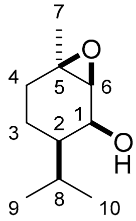	**Name**	6-Methyl-3-(1-methylethyl)-7-oxabicyclo [4.1.0] heptan-2-ol (**19**)
	**Physical state**	Colorless oil
	**Molecular formula**	$C_{10}H_{18}O_2$
	**Molecular weight**	170 g/mol
	**Specific rotation**	$[\alpha]_D^{25}$ = -45.8 (c = 1.0, $CHCl_3$)
^{1}H-NMR ($CDCl_3$, 400 MHz): δ_H (ppm) 4.19-4.11 (m, 1H, H-1), 3.23 (dd, *J* = 5.5, 1.0 Hz, 1H, H-6), 2.09-1.98 (m, 2H, H-8 y OH), 1.69-1.56 (m, 2H, H-4), 1.42-1.33 (m, 2H, H-2 y H-3), 1.34 (s, 3H, H-7), 1.19-1.06 (m, 1H, H-3), 0.98 (d, *J* = 6.6 Hz, H-9), 0.90 (d, *J* = 6.7 Hz, H-10).		
^{13}C-NMR (APT) ($CDCl_3$, 100 MHz): δ_C (ppm) 64.7 (C-1), 62.3 (C-6), 61.5 (C-5), 47.0 (C-2), 31.2 (C-4), 27.9 (C-8), 23.0 (C-7), 21.0 (C-9 y C-10), 17.5 (C-3).		
Bibliography: [59]		

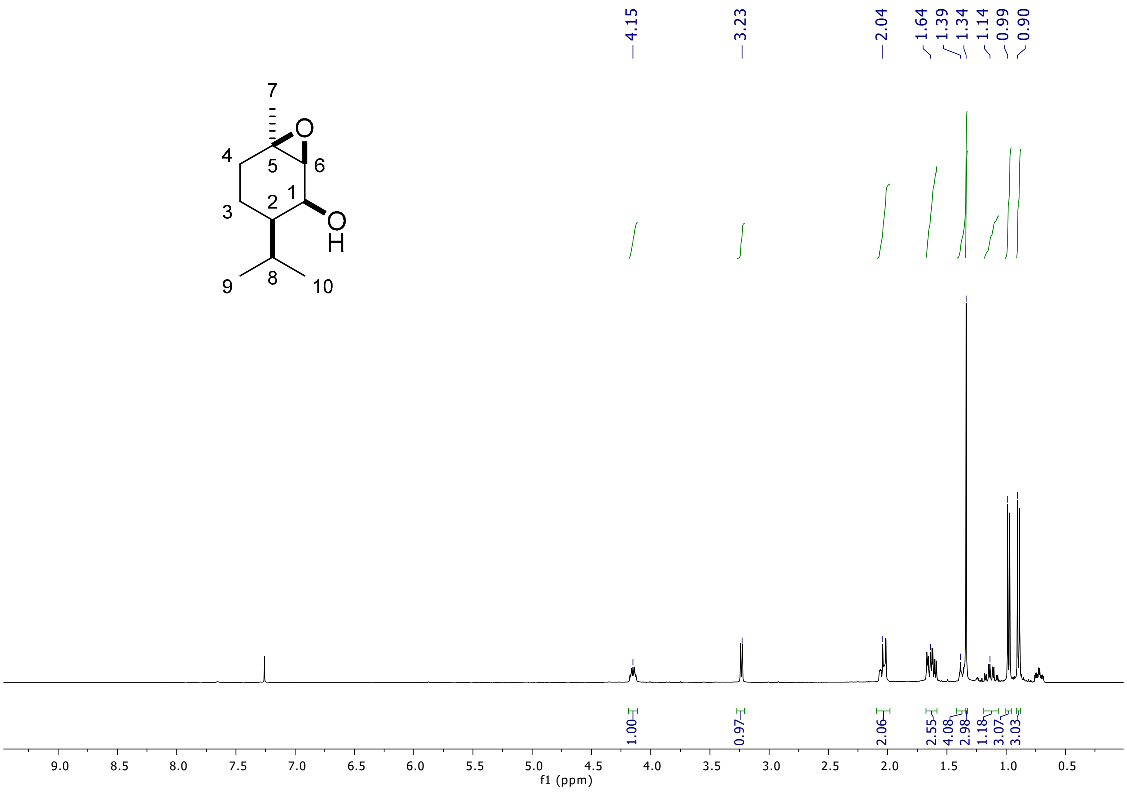

Figure S25. ^{1}H -NMR spectra of 6-Methyl-3-(1-methylethyl)-7-oxabicyclo [4.1.0] heptan-2-ol (**19**)

International Journal of
Molecular Sciences

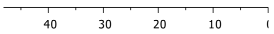

Figure S26. ^{13}C-NMR (APT) spectra of 6-Methyl-3-(1-methylethyl)-7-oxabicyclo [4.1.0] heptan-2-ol (**19**).

International Journal of
Molecular Sciences

MDPI

14. Physical and spectroscopic characterization of 2,3-dihydroxy-6-isopropyl-3-methylcyclohexan-1-one (20) (Figure S27 and S28, Table S18).

Table S18. Spectroscopic characterization of 2,3-dihydroxy-6-isopropyl-3-methylcyclohexan-1-one (**20**).

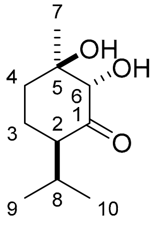	**Name**	2,3-dihydroxy-6-isopropyl-3-methylcyclohexan-1-one (**20**)
	**Physical state**	Colorless oil
	**Molecular formula**	$C_{10}H_{18}O_2$
	**Molecular weight**	170 g/mol
	**Specific rotation**	$[\alpha]_D^{25}$ = -30.0 (c = 1.0, $CHCl_3$)

^{1}H-NMR ($CDCl_3$, 400 MHz): δ_H (ppm) 4.09 (s, 1H, H-6), 2.66-2.57 (m, 1H, H-2), 2.16-2.06 (m, 2H, H-4), 1.86-1.79 (m, 3H, H-3 y H-8), 1.38 (s, 3H, H-7), 0.95 (d, *J* = 4.7 Hz, 3H, H-9), 0.94 (d, *J* = 4.7 Hz, 3H, H-10).
^{13}C-NMR (APT) ($CDCl_3$, 100 MHz): δ_C (ppm) 205.2 (C-1), 76.9 (C-2), 68.8 (C-3), 51.5 (C-6), 32.7 (C-4), 26.6 (C-8), 25.6 (C-7), 22.8 (C-5), 21.1 (C-9), 19.4 (C-10).
Bibliography: [83]

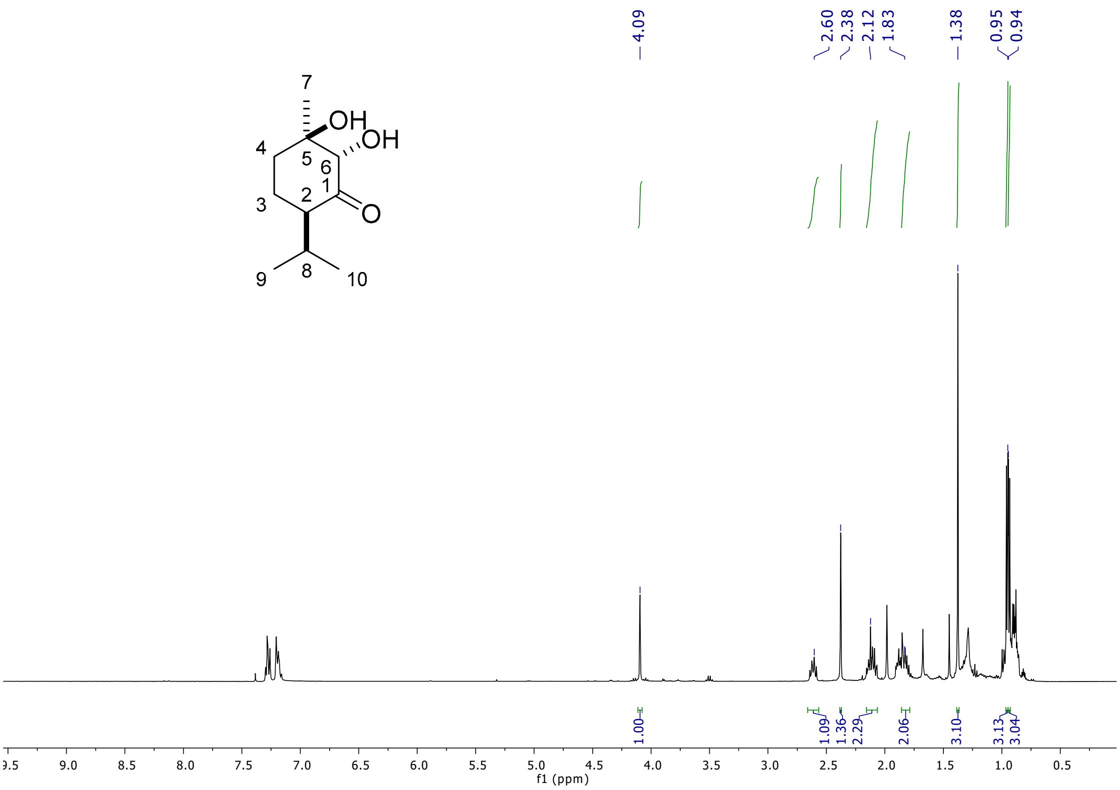

Figure S27. ^{1}H -NMR spectra of 2,3-dihydroxy-6-isopropyl-3-methylcyclohexan-1-one (**20**).

International Journal of
Molecular Sciences

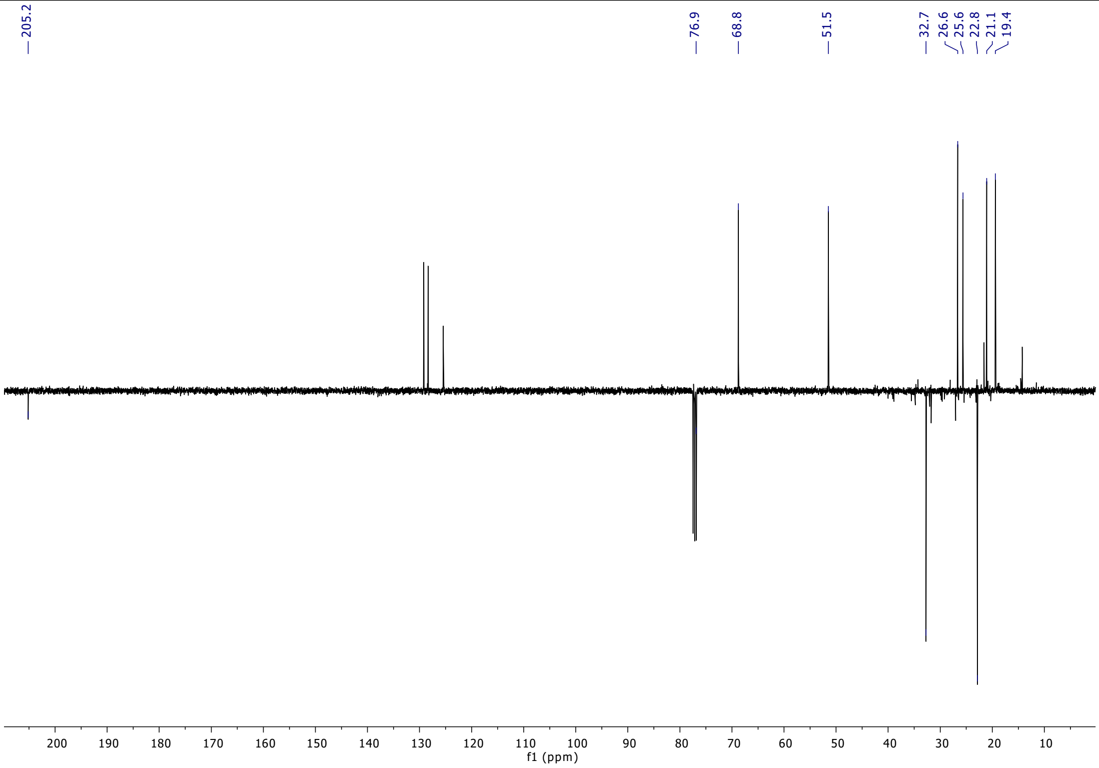

Figure S28. ^{13}C-NMR (APT) spectra of 2,3-dihydroxy-6-isopropyl-3-methylcyclohexan-1-one (**20**).

International Journal of *Molecular Sciences* MDPI

15. Physical and spectroscopic characterization of Pulegone oxide (21a) (Figure S29 and S30, Table S19).

Table S19. Spectroscopic characterization of Pulegone oxide (**21a**).

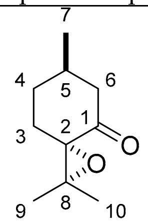	**Name**	Pulegone oxide (**21a**)
	**Physical state**	Colorless oil
	**Molecular formula**	$C_{10}H_{18}O_2$
	**Molecular weight**	168 g/mol
	**Specific rotation**	$[\alpha]_D^{25}$ = -88.3 (c = 1.0, $CHCl_3$)
^{1}H-NMR ($CDCl_3$, 400 MHz): δ_H (ppm) 2.49-2.32 (m, 2H, H-6), 2.30-2.07 (m, 1H, H-5), 2.06-1.93 (m, 2H, H-4), 1.92-1.75 (m, 2H, H-3), 1.42 (s, 3H, H-9), 1.21 (d, *J* = 3.5 Hz, 3H, H-10), 1.06 (d, *J* = 6.0 Hz, 3H, H-7).		
^{13}C-NMR (APT) ($CDCl_3$, 100 MHz): δ_C (ppm) 207.8 (C-1), 70.3 (C-2), 63.6 (C-8), 49.6 (C-6), 30.8 (C-5), 30.3 (C-4), 26.4 (C-3), 20.1 (C-7), 19.8 (C-9), 19.8 (C-10).		
Bibliography: [55]		

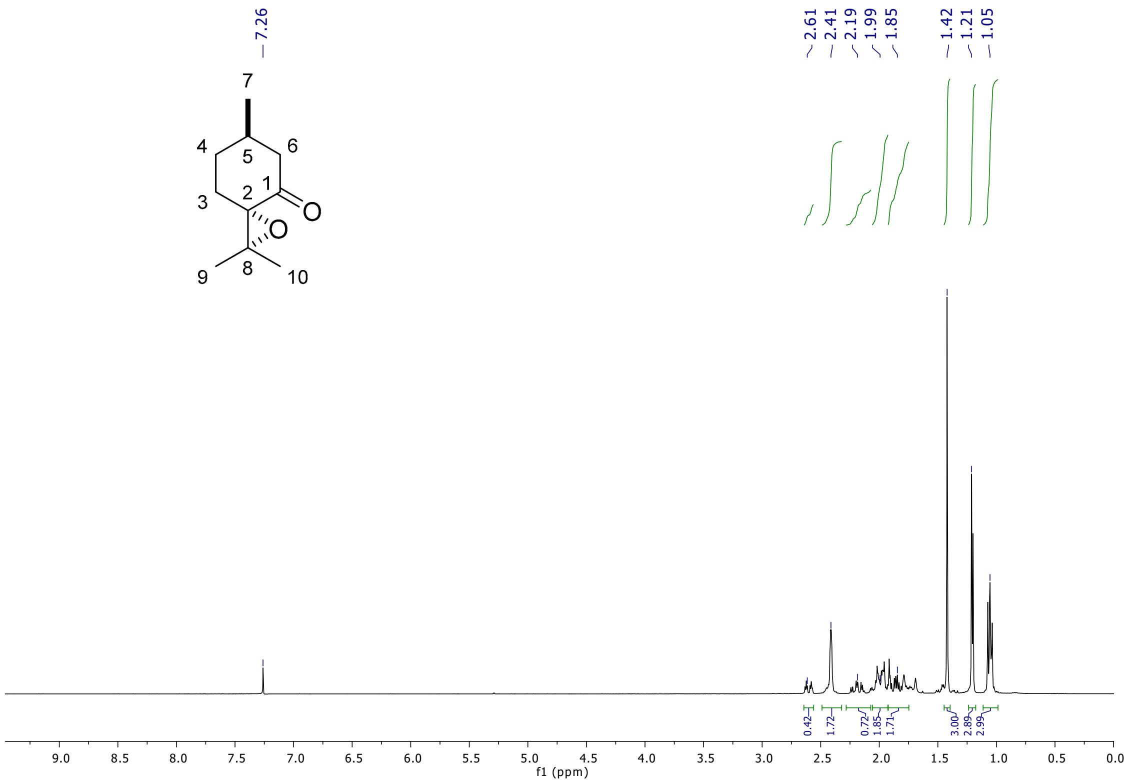

Figure S29. ^{1}H -NMR spectra of pulegone epoxide (**21a**).

International Journal of
Molecular Sciences
MDPI

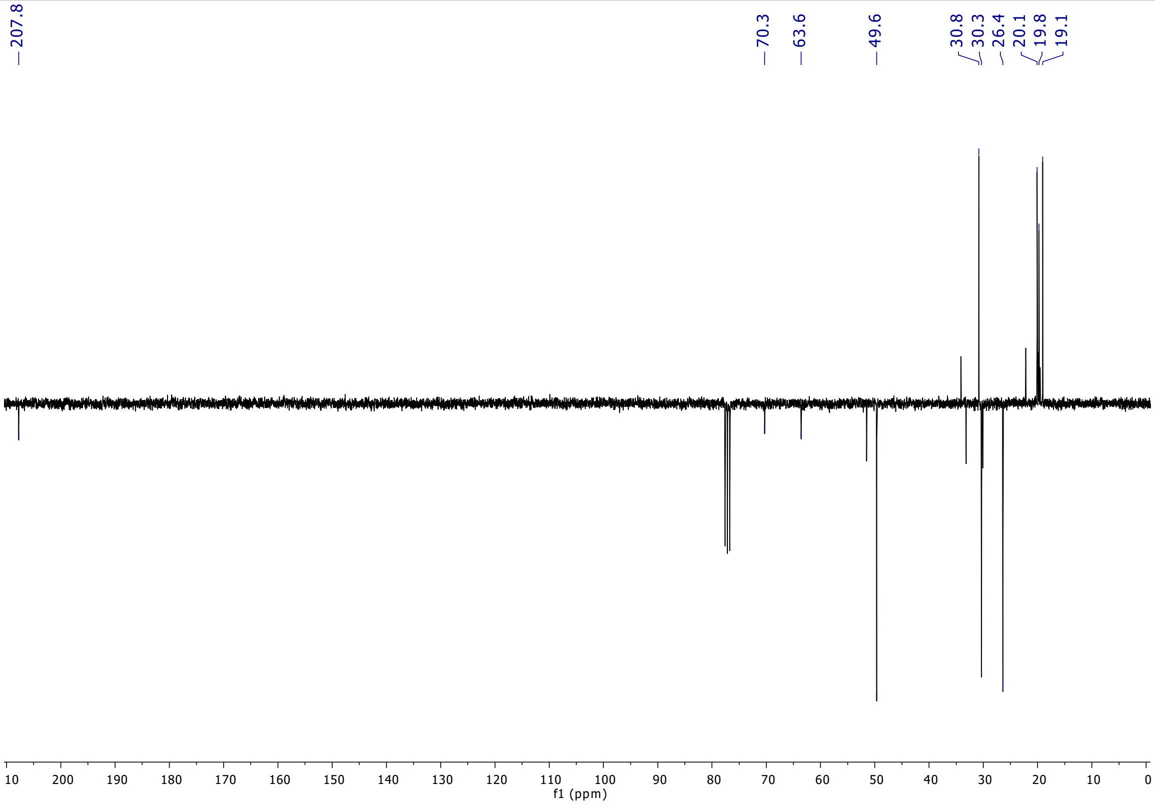

Figure S30. ^{13}C-NMR (APT) spectra of pulegone epoxide (**21a**).

International Journal of
Molecular Sciences
MDPI

16. Physical and spectroscopic characterization of Pulegone hydrochloride (22) (Figure S31 and S32, Table S20).

Table S20. Spectroscopic characterization of Pulegone hydrochloride (**22**).

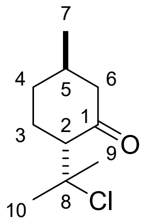	**Name**	Pulegone hydrochloride (**22**)
	**Physical state**	slightly yellow oil
	**Molecular formula**	$C_{10}H_{17}ClO$
	**Molecular weight**	188 g/mol
	**Specific rotation**	$[\alpha]_D^{25}$ = -33.3 (c = 1.0, $CHCl_3$)
^{1}H-NMR ($CDCl_3$, 400 MHz): δ_H (ppm) 2.68 (ddd, *J* = 13.0, 4.6, 1.2 Hz, 1H, H-2), 2.55-2.47 (m, 1H, H-6), 2.27 (ddd, *J* = 12.3, 4.1, 2.2 Hz, 1H, H-6), 2.01 (td, *J* = 12.5, 1.2 Hz, 1H, H-5), 1.92-1.79 (m, 2H, H-3), 1.71 (s, 3H, H-10), 1.61 (s, 3H, H-9), 1.54 (td, *J* = 13.0, 3.2 Hz, 1H, H-4), 1.37 (tdd, *J* = 12.9, 11.3, 3.5 Hz, 1H, H-4), 0.98 (d, *J* = 6.3 Hz, 3H, H-7).		
^{13}C-NMR (APT) ($CDCl_3$, 100 MHz): δ_C (ppm) 209.3 (C-1), 72.3 (C-8), 61.3 (C-2), 51.9 (C-6), 36.6 (C-5), 34.2 (C-4), 32.2 (C-9), 29.6 (C-3), 27.9 (C-10), 22.2 (C-7).		
Bibliography: [56]		

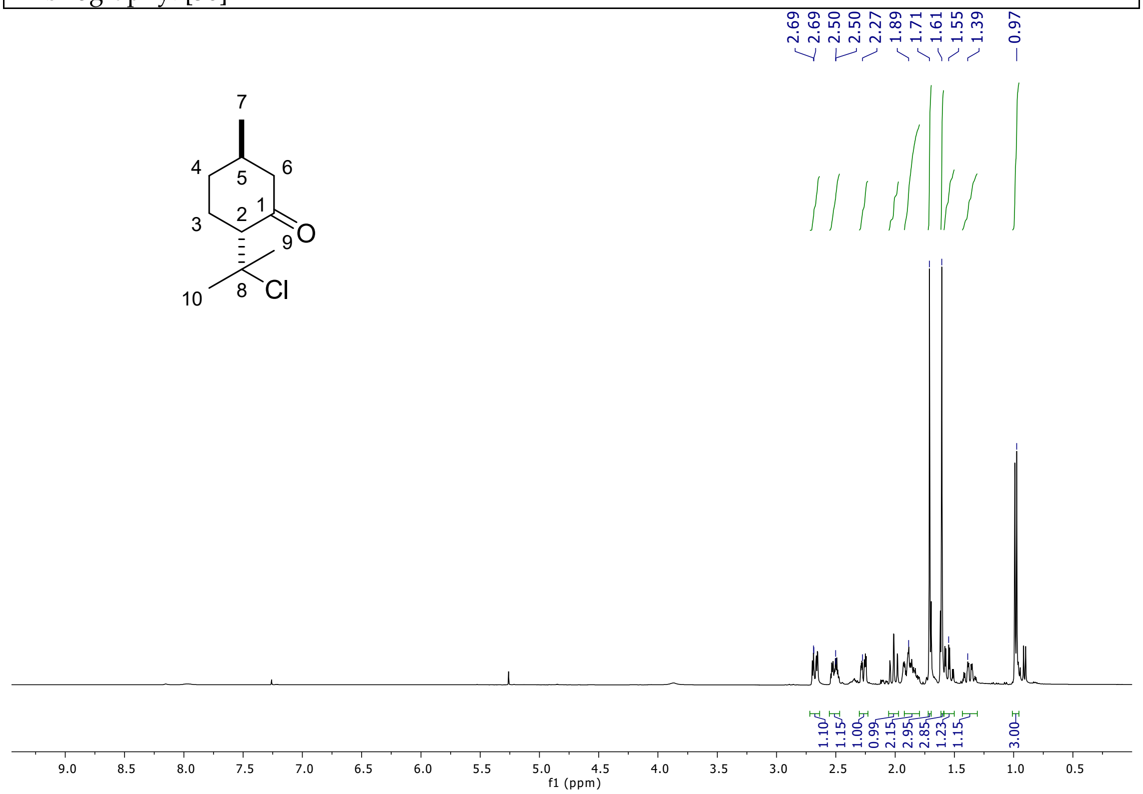

Figure S31. ^{1}H -NMR spectra of pulegone hydrochloride (**22**).

International Journal of
Molecular Sciences

MDPI

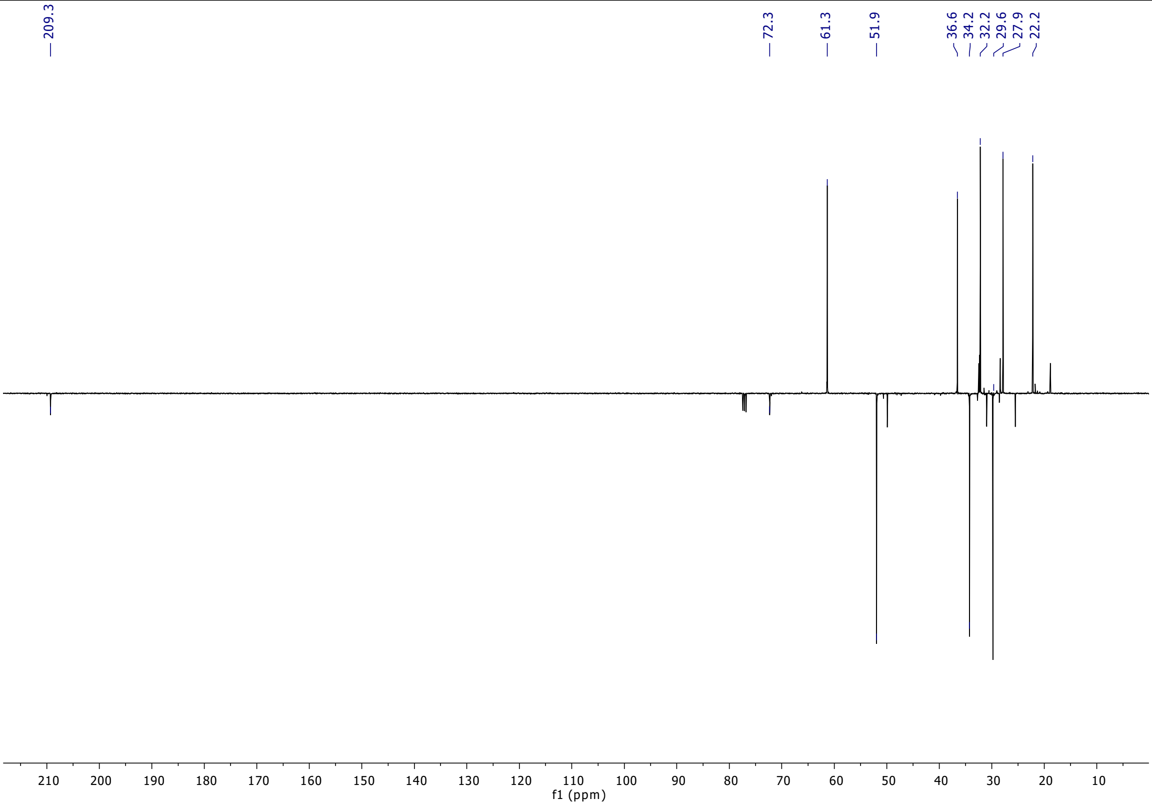

Figure S32. ^{13}C-NMR (APT) spectra of pulegone hydrochloride (**22**).

International Journal of *Molecular Sciences* MDPI

17. Physical and spectroscopic characterization of p-menth-4-en-3-one (23) (Figure S33 and S34, Table S21).

Table S21. Spectroscopic characterization of p-menth-4-en-3-one (**23**).

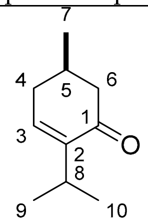	**Name**	p-menth-4-en-3-one (**23**)
	**Physical state**	Colorless oil
	**Molecular formula**	$C_{10}H_{16}O$
	**Molecular weight**	152 g/mol
	**Specific rotation**	N/A
^{1}H-NMR ($CDCl_3$, 400 MHz): δ_H (ppm) 6.62 (ddd, J = 5.7, 2.7, 1.1 Hz, 1H, H-3), 2.88-2.79 (m, 1H, H-8), 2.50-2.35 (m, 2H, H-6), 2.12-1.96 (m, 3H, H-5 and H-4), 1.01 (d, J = 6.2 Hz, 3H, H-7), 0.98 (d, J = 2.6 Hz, 3H, H-9), 0.97 (d, J = 2.6 Hz, 3H, H-10).		
^{13}C-NMR (APT) ($CDCl_3$, 100 MHz): δ_C (ppm) 199.4 (C-1), 145.4 (C-2), 141.5 (C-3), 47.1 (C-6), 34.4 (C-4), 30.5 (C-5), 26.3 (C-8), 22.1 (C-9), 21.9 (C-10), 21.3 (C-7).		
Bibliography: [58]		

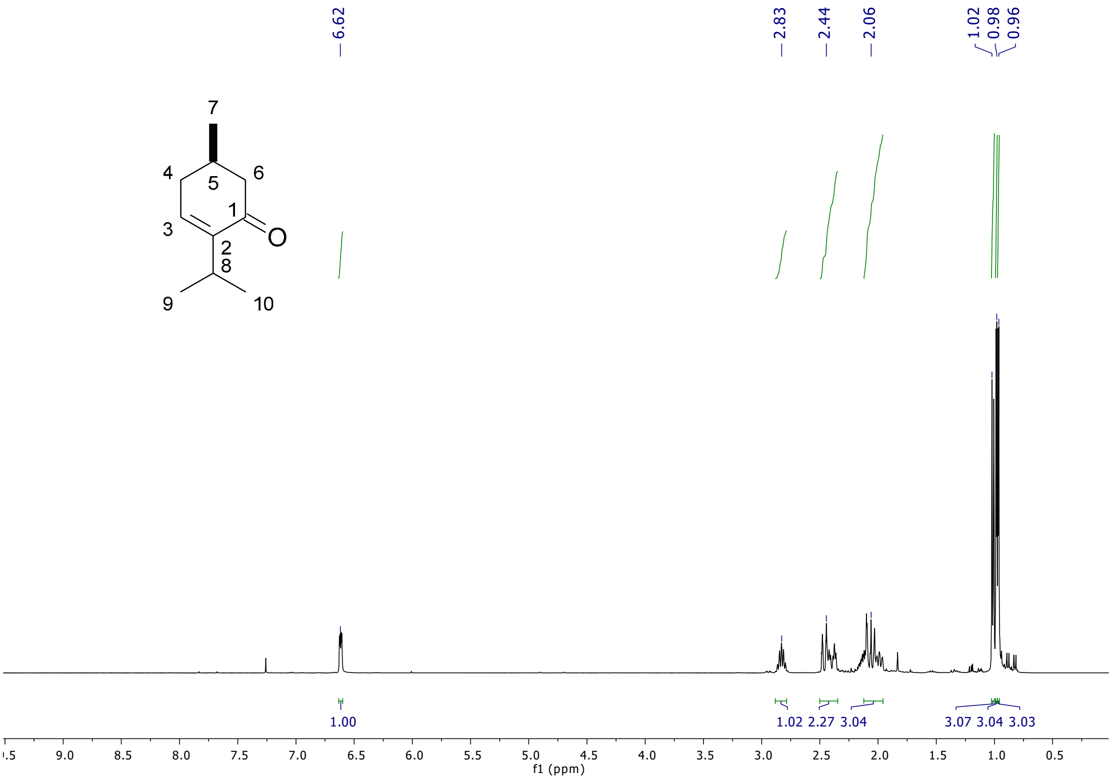

Figure S33. ^{1}H -NMR spectra of p-menth-4-en-3-one (**23**).

International Journal of
Molecular Sciences

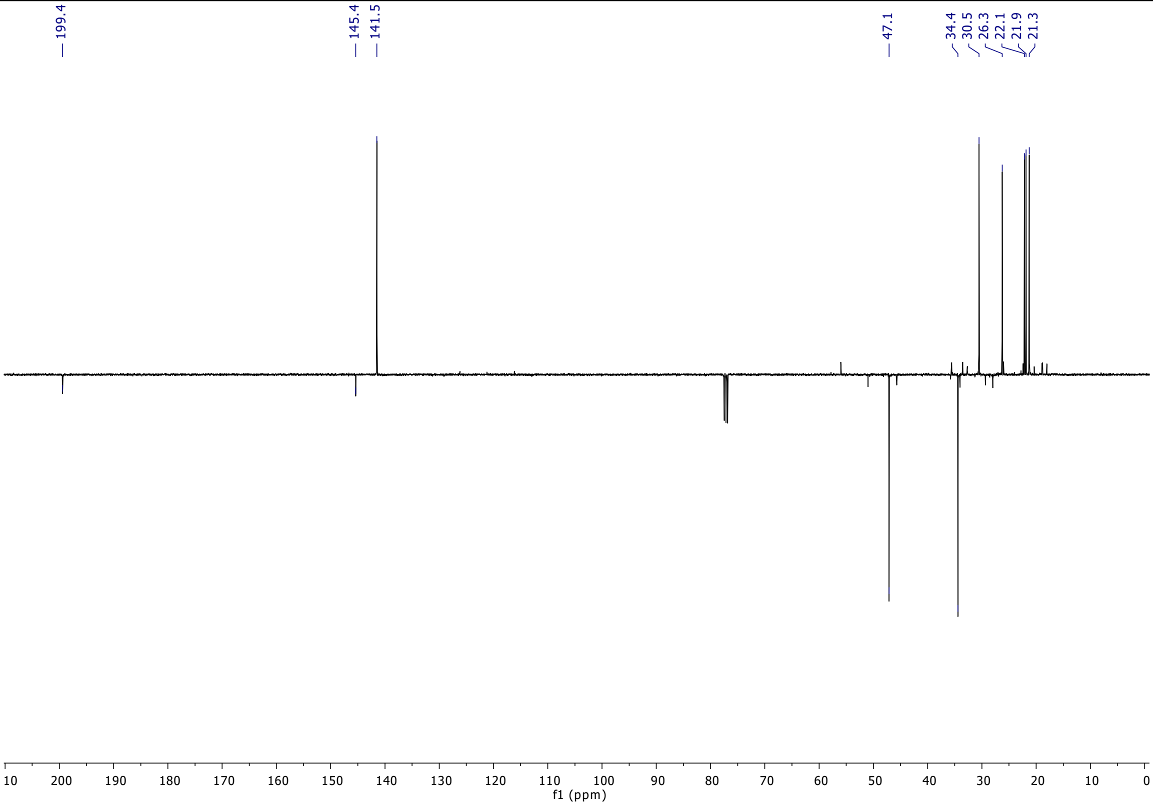

Figure S34. ^{13}C-NMR (APT) spectra of p-menth-4-en-3-one (**23**).

International Journal of
Molecular Sciences

MDPI

18. Physical and spectroscopic characterization of Pulegol (24) (Figure S35 and S36, Table S22).

Table S22. Spectroscopic characterization of Pulegol (**24**).

<table>
<tr><td rowspan="5">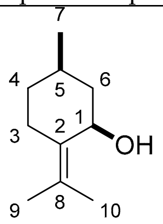</td><td>Name</td><td>Pulegol (24)</td></tr>
<tr><td>Physical state</td><td>Colorless oil</td></tr>
<tr><td>Molecular formula</td><td>$C_{10}H_{16}O$</td></tr>
<tr><td>Molecular weight</td><td>154 g/mol</td></tr>
<tr><td>Specific rotation</td><td>$[\alpha]_D^{25}$ = -85.9 (c = 1.0, $CHCl_3$)</td></tr>
<tr><td colspan="3">^{1}H-NMR ($CDCl_3$, 400 MHz): δ_H (ppm) 4.73 (t, J = 4.8 Hz, 1H, H-1), 2.35-2.19 (m, 2H, H-3), 1.80 (s, 3H, H-9), 1.70 (s, 3H, H-10), 1.66-1.53 (m, 3H, H-5 y H-6), 1.52-1.36 (m, 2H, H-4), 1.13 (d, J = 6.8 Hz, 3H, H-7).</td></tr>
<tr><td colspan="3">^{13}C-NMR (APT) ($CDCl_3$, 100 MHz): δ_C (ppm) 132.7 (C-2), 126.7 (C-8), 68.3 (C-1), 39.5 (C-6), 31.9 (C-4), 26.7 (C-5), 22.2 (C-3), 21.6 (C-7), 20.6 (C-9), 19.9 (C-10).</td></tr>
<tr><td colspan="3">Bibliography: [60]</td></tr>
</table>

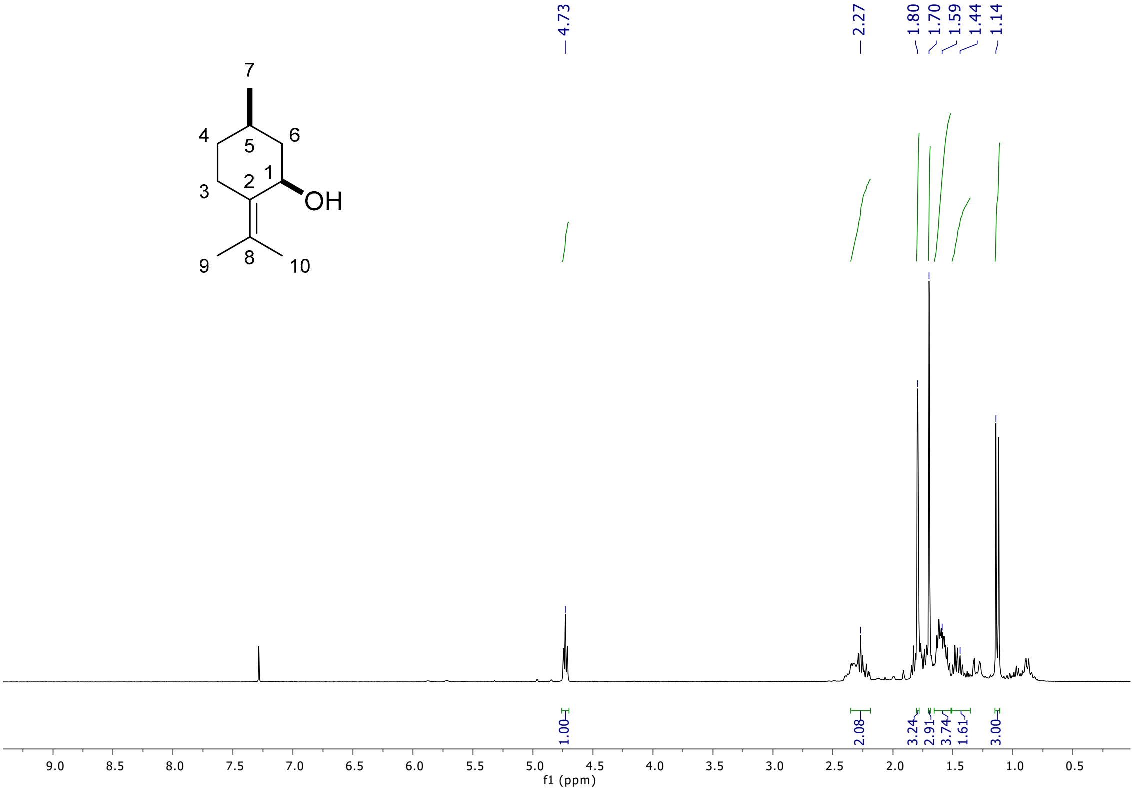

Figure S35. ^{1}H -NMR spectra of pulegol (**24**).

International Journal of
Molecular Sciences

MDPI

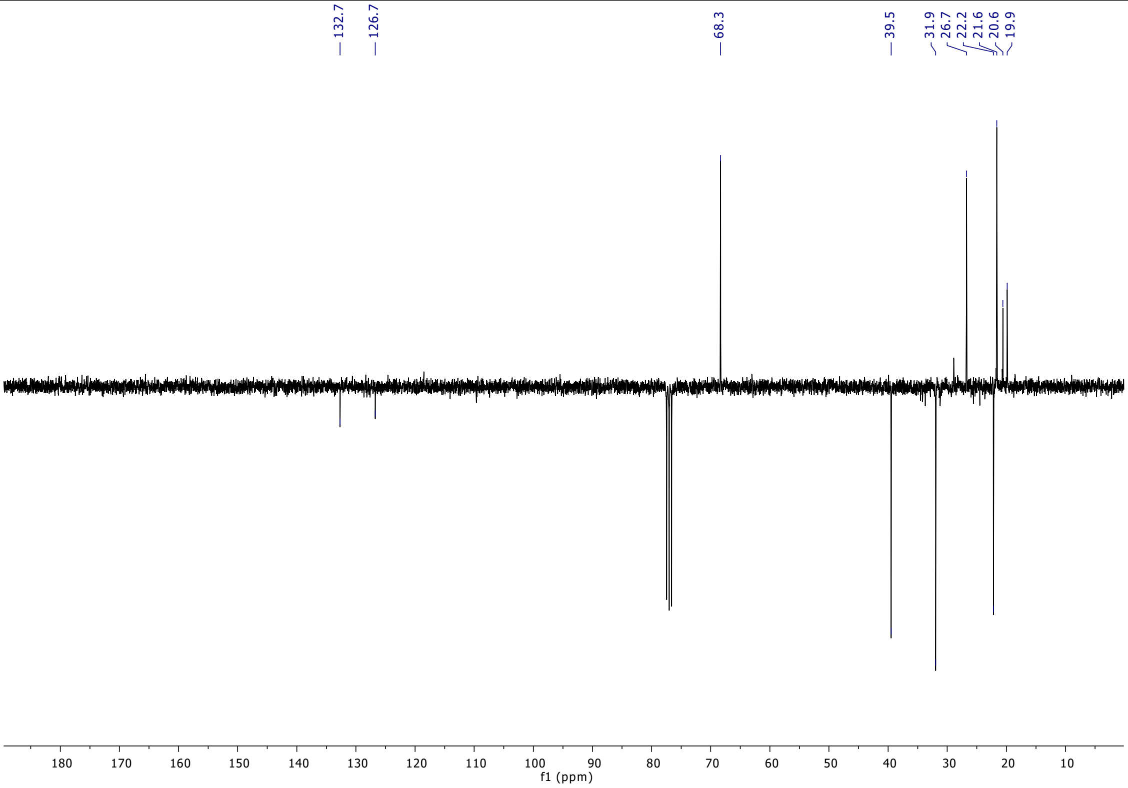

Figure S36. ^{13}C-NMR (APT) spectra of pulegol (**24**).

19. Statistical treatment of insecticidal activity of EOs and isolated, commercial, and synthesized compounds (Table S23 and S24).

Table S23. Statistical analysis of insecticidal activity through fumigant toxicity of essential oils (EOs) and compounds.

Substance	*S zeamais*				*T. castaneum*			
	LC_{50}	slop	intercept	p value	LC_{50}	slop	intercept	p value
T. zypaquirensis	23.1(12.7-33.3)	3.829	-8.633	<0.05	104.4 (74.4-143.2)	8.607	-17.292	<0.05
S. viminea	6.4 (3.9-9.7)	0,055	-1.238	<0.05	20.6 (12.2-31.9)	0.903	-1.598	<0.05
M. mollis	4.8 (3.3-6.8)	0.485	-2.216	<0.05	7.0 (6.1-8.0)	0.236	-1.333	<0.05
A. graveolens	15.5 (13.1 - 18.3)	0.235	-3.723	<0.05	40.1(35.4 - 46.1)	0,113	-4.614	<0.05
1	88.8 (81.9-95.0)	6.636	-12.929	<0.05	13.5 (12.1-13.0)	6.425	-7.260	<0.05
2	42.4 (28.7-63.3)	2.016	-3.280	<0.05	4.3 (1.3-7.6)	1.416	-0,899	<0.05
3	91.8 (87.0-99.7)	14.326	-28.122	<0.05	11.3 (14.1-9.0)	3.133	-3.296	<0.05
4	92.0 (84.9-107.8)	11.897	-23.366	<0.05	24.0 (14.2-31.1)	0.063	-1.512	<0.05
5	14.5 (12.9-16.1)	5.571	-6.476	<0.05	4.8 (3.4-6.7)	2.009	-1.375	<0.05
6	3.0 (3.3-2.7)	5.524	-2.633	<0.05	2.2 (2.4-2.0)	7.927	-2.708	<0.05
7	NA	NA	NA	NA	NA	NA	NA	NA
8	NA	NA	NA	NA	NA	NA	NA	NA
9	180.4(161.1-208.2)	2.649	-5.976	<0.05	42.1 (46.6-38.3)	0.284	-11.964	<0.05
10	37.1 (32.5-42.3)	7.499	-11.770	<0.05	16.9 (9.9-23.4)	1.897	-2.333	<0.05
11	104.0 (88.6-122.9)	3.757	-7.579	<0.05	75.1 (57.9-111.0)	3.579	-6.715	<0.05
12	NA	NA	NA	NA	NA	NA	NA	NA
13	25.4 (22.0-29.4)	3.425	-4.813		2.7 (1.9-3.5)	2.464	-1.074	<0.05
14	NA	NA	NA	NA	2.8 (1.6-3.9)	3.294	-1.483	<0.05
15	NA	NA	NA	NA	NA	NA	NA	NA
16	NA	NA	NA	NA	25.4 (20.8-31.0)	0.103	-2756	<0.05
17	NA	NA	NA	NA	NA	NA	NA	NA
18	NA	NA	NA	NA	NA	NA	NA	NA
19	NA	NA	NA	NA	NA	NA	NA	NA
20	41.8 (34.6-51.2)	0.081	-3.474	<0.05	4.5 (3.5-5.5)	0.586	-2.730	<0.05
21	NA	NA	NA	NA	1.1 (0.1-3.7)	0.802	-0,022	<0.05
22	42.2 (36.6-49.2)	3.488	-5.669	<0.05	35.2 (27.3-44.0)	0.084	-2.998	<0.05
23	92.9 (74.4-124.0)	0.028	-2.696	<0.05	1.4 (0.1-3.9)	0.362	-0,661	<0.05
24	NA	NA	NA	NA	NA	NA	NA	NA
Nuvan 50 ®	2.1 (1.5-3.8)	0.250	-1.045	<0.05	1.0 (0.1-2.0)	0.842	-1.131	<0.05

International Journal of Molecular Sciences

MDPI

Table S24. Statistical analysis of insecticidal activity through contact toxicity of essential oils (EOs) and compounds.

Substance	*S zeamais*				*T. castaneum*			
	LD_{50}	slop	intercept	p value	LD_{50}	slop	intercept	p value
T. zypaquirensis	91.1 (71.7-111.4)	0,039	-3.520	<0.05	66.6 (49.8-87.7)	0.037	-2.502	<0.05
S. viminea	24.6 (12.8-38.3)	0.039	-1.016	<0.05	16.4 (10.9-24.9)	0.089	-1.567	<0.05
M. mollis	15.8 (9,3-24,2)	0,081	-1.367	<0.05	6.5 (4.6-9.3)	0.282	-1.934	<0.05
A. graveolens	140.3 (124.5 - 160.6)	0,035	-4.966	<0.05	86.1 (75.1 - 99.3)	0,058	-5.022	<0.05
1	66.7 (54.0-87.7)	0.040	-2.704	<0.05	9.7 (8.2-18.0)	0.124	-1.416	<0.05
2	16.3 (14.4-18.2)	0.391	-6.250	<0.05	4.8 (2.9-6.7)	0.372	-1.729	<0.05
3	NA	NA	NA	NA	73.1 (58.8-87.4)	0.049	-3.558	<0.05
4	NA	NA	NA	NA	27.0 (12.7-44.8)	0,044	-1	<0.05
5	24.6 (14.4-37.4)	0.055	-1.357	<0.05	5.9 (5.1-6.8)	0.832	-5	<0.05
6	14.9 (12.1-18.7)	0.310	-5.077	<0.05	13.1 (10.3-15.9)	0.253	-3.251	<0.05
7	NA	NA	NA	NA	NA	NA	NA	NA
8	75.6 (52.1-120.9)	0.018	-1.496	<0.05	78.6 (52.9-123.2)	0.020	-1.608	<0.05
9	NA	NA	NA	NA	88.1 (65.1-120.3)	0.024	-2.164	<0.05
10	50.8 (43.6-59.0)	0.083	-4.214,000	<0.05	49.0 (37.2-61.7)	0,055	-2.675	<0.05
11	NA	NA	NA	NA	105.2 (86.3-131.8)	0.032	-3.416	<0.05
12	NA	NA	NA	NA	NA	NA	NA	NA
13	38.7 (26.6-57.6)	0.041	-1.592	<0.05	16.3 (12.0-23.2)	0.131	-2.164	<0.05
14	14.2(8.7-19.0)	0.125	-1.673	<0.05	5.5 (4.7-7.1)	0.602	-3.383	<0.05
15	44.0 (35.4-55.5)	0.057	-2.529	<0.05	21.1 (13.4-29.7)	0.091	-1.867	<0.05
16	38.8 (32.1-45.4)	0.064	-2.809	<0.05	19.8 (17.0-21.7)	0.309	-5.950	<0.05
17	69.1 (47.3-105.5)	0.019	-1.417	<0.05	85.5 (70.9-107.4)	0.040	-3.465	<0.05
18	28.3 (26.4-31.3)	0.246	-6.998	<0.05	1.0 (0.7-3.9)	0.369	-0,519	<0.05
19	109.7 (67.3-158.7)	0.013	-1.459	<0.05	67.3 (61.5-78.5)	0.070	-4.919	<0.05
20	24.5 (17.6-31.4)	0.089	-2.140	<0.05	1.9 (1.0-4.9)	0.322	-0,785	<0.05
21	263.0 (153.0-308.7)	0.007	-1.769	<0.05	80.0 (60.0-90.0)	0.049	-3.788	<0.05
22	30.2 (14.6-50.7)	0.034	-1,034	<0.05	16.6 (13.6-20.5)	0.224	-3.816	<0.05
23	45.1 (36.2-56.8)	0.062	-2.808	<0.05	17.6 (9.8-29.4)	0.078	-1.514	<0.05
24	NA	NA	NA	NA	NA	NA	NA	NA
Cypermethrin	1.0 (0.1-2.0)	0.250	-1.045	<0.05	10.5 (0.1-20.0)	0.105	-1.416	<0.05
